# Enhanced lysosomal degradation maintains the quiescent state of neural stem cells

**DOI:** 10.1038/s41467-019-13203-4

**Published:** 2019-11-29

**Authors:** Taeko Kobayashi, Wenhui Piao, Toshiya Takamura, Hiroshi Kori, Hitoshi Miyachi, Satsuki Kitano, Yumiko Iwamoto, Mayumi Yamada, Itaru Imayoshi, Seiji Shioda, Andrea Ballabio, Ryoichiro Kageyama

**Affiliations:** 1 Institute for Frontier Life and Medical Sciences, Kyoto University, Kyoto, 606-8507 Japan; 2 Graduate School of Medicine, Kyoto University, Kyoto, 606-8501 Japan; 3 Graduate School of Biostudies, Kyoto University, Kyoto, 606-8501 Japan; 4 Department of Complexity Science and Engineering, University of Tokyo, Tokyo, 277-8561 Japan; 5 Institute for Integrated Cell-Material Sciences (iCeMS), Kyoto University, Kyoto, 606-8501 Japan; 6 Peptide Drug Innovation, Global Research Center for Innovative Life Science (GRIL), Hoshi University, 2-4-41 Ebara, Shinagawa-ku, Tokyo, 142-8501 Japan; 7 Telethon Institute of Genetics and Medicine, Via Campi Flegrei 34, 80078 Pozzuoli, NA Italy

**Keywords:** Molecular neuroscience, Stem cells in the nervous system, Neural stem cells, Quiescence

## Abstract

Quiescence is important for sustaining neural stem cells (NSCs) in the adult brain over the lifespan. Lysosomes are digestive organelles that degrade membrane receptors after they undergo endolysosomal membrane trafficking. Enlarged lysosomes are present in quiescent NSCs (qNSCs) in the subventricular zone of the mouse brain, but it remains largely unknown how lysosomal function is involved in the quiescence. Here we show that qNSCs exhibit higher lysosomal activity and degrade activated EGF receptor by endolysosomal degradation more rapidly than proliferating NSCs. Chemical inhibition of lysosomal degradation in qNSCs prevents degradation of signaling receptors resulting in exit from quiescence. Furthermore, conditional knockout of TFEB, a lysosomal master regulator, delays NSCs quiescence in vitro and increases NSC proliferation in the dentate gyrus of mice. Taken together, our results demonstrate that enhanced lysosomal degradation is an important regulator of qNSC maintenance.

## Abstract (web summary)

It remains unclear why quiescent neural stem cells (qNSCs) in the subventricular zone of the mouse brain have enlarged lysosomes. Here, authors demonstrate that qNSCs exhibit higher lysosomal activity and degrade activated EGF receptor by endolysosomal degradation more rapidly than proliferating NSCs, which prevents the NSC exit from quiescence.

## Introduction

Somatic stem cells in adult tissues maintain the capacity to self-renew and produce progeny through the lifespan. As with other somatic stem cells, the majority of neural stem cells (NSCs) in the adult mammalian brain exist in a quiescent state, which is essential for maintenance of stem cell pools over long periods of time^1,2^. In the adult mouse brain, NSCs primarily reside in two regions, the hippocampal dentate gyrus (DG) and the subventricular zone (SVZ) of the lateral ventricle. Quiescent NSCs (qNSCs) remain in the G0 phase and re-enter the cell cycle after receiving activation signals; once activated, they generate proliferating NSCs (active NSCs; aNSCs), which differentiate into transit-amplifying cells and ultimately into mature neurons^3^. Extensive studies have identified the factors, signaling pathways, and metabolic states underlying the transition between NSC proliferation and quiescence in vivo and in vitro^4–7^. However, the role of proteostasis in the regulation of this process remains poorly understood.

Proteolysis, mediated primarily by the ubiquitin–proteasome and autophagy–lysosome pathways, is essential for cellular proteostasis^8^. Elevated proteasomal activity is crucial for stem cell identity, and prevents differentiation and senescence in human embryonic stem cells^9^. Autophagic activity decreases over the course of aging in muscle stem cells, resulting in loss of stemness in geriatric satellite cells^10^. These observations suggest that global proteolysis preserves stemness by governing basal proteostasis. Notably, qNSCs in the DG express abundant functional membrane receptors, which are required for aNSCs, despite remaining in a resting state^11^, implying that specifically regulated proteolysis is required to maintain quiescence in these cells. Recent work showed that lysosomes are enriched in qNSCs, compared to aNSCs in the SVZ^12^, in which aged qNSCs accumulate more protein aggregates in lysosomes while a decrease in the abundance of aggregates causes qNSCs to rejuvenate. However, the functional role of lysosomes in qNSCs, especially at the transition between aNSCs and qNSCs, is not fully understood. In this study, we show that lysosomal activity is elevated in qNSCs, and that inhibition of this activity leads to exit from quiescence via a process dependent on activation of epidermal growth factor (EGF) and Notch signaling. Conversely, lysosomal activation in aNSCs promotes exit from proliferation. In vitro, activated EGF receptor (EGFR) is more rapidly degraded in qNSCs than in aNSCs. Furthermore, knockout of *Tfeb*, a master regulator of lysosomal biogenesis, in NSCs delays not only the reduction in EGF and Notch signaling but also the entry into quiescence in vitro. Importantly, in vivo, conditional knockout of *Tfeb* in adult NSCs increases the number of proliferating NSCs, along with the levels of activated EGFR and Notch1 in the DG of the hippocampus. These findings demonstrate that enhanced lysosomal activity enables NSCs to remain poised in the quiescent state by rapidly removing unnecessary or undesirable cellular signals.

## Results

### NSCs increase lysosomal activity when they enter quiescence

**Fig. 1:**
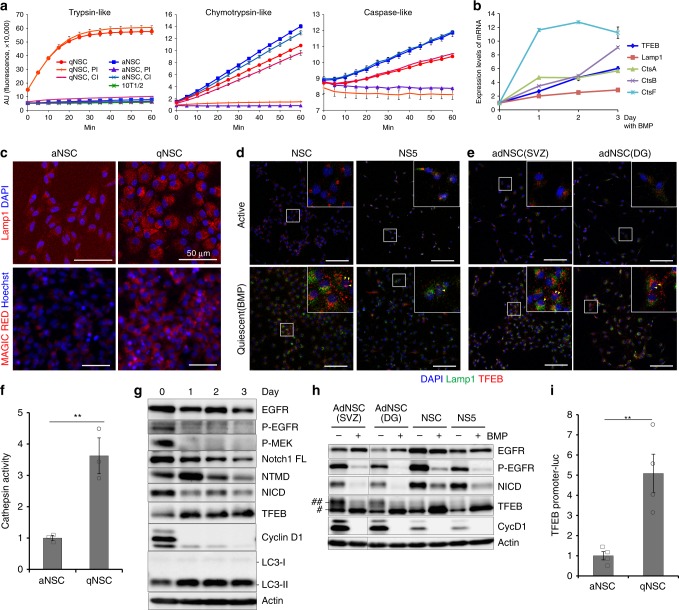
Increased lysosomal activity in qNSCs in vitro. **a** Peptidase activities in NSCs. Trypsin-like, chymotrypsin-like, and caspase-like activities in NSC lysate were continuously measured every 5 min for 1 h, with or without proteasome inhibitor (PI) or cathepsin inhibitor (CI); *n* = 2. **b** qPCR of the lysosomal genes: Lamp1 and cathepsins (CtsA, CtsB, and CtsF), and the transcriptional regulator TFEB, after BMP exposure. **c** Immunocytochemistry of Lamp1 (red) with DAPI (blue) (upper panels). Staining of Magic Red cathepsin L (red) and Hoechst 33342 (blue) (lower panels). Scale bars, 50 µm. **d**, **e** Immunocytochemistry of Lamp1 and TFEB in the active and quiescent states induced by BMP in NSCs, NS5 cells (**d**) and adult NSCs (**e**). Selected regions indicated by white squares are enlarged. Yellow arrowheads in enlarged squares indicate nuclear localization of TFEB in the quiescent state. Scale bars, 100 µm. **f** Lysosomal cathepsin L activity in NSCs, as determined using Magid Rred cathepsin L; *n* = 3. Corresponding data points were plotted. **g** Immunoblots of signaling molecules in NSCs after BMP exposure. β-Actin was used as a loading control. **h** Immunoblotting of several NSC lines in the active and quiescent states. Phosphorylated (##) and dephosphorylated forms of TFEB (#) were detected. **i** Reporter assay of TFEB-promoter–driven luciferase; *n* = 4. Data represent means ± s.e.m. (**P < 0.01; Student’s *t-*test). Source data of immunoblots are provided as a Source Data file.

We first investigated whether proteasomal activity differs significantly between quiescent and proliferating NSCs (Fig. 1a). For these experiments, we used an in vitro culture model of NSCs, an NSC line established in our previous study^13^ (see Methods) as well as other NSCs including NS5, ES cell-derived NSCs, and NSCs from adult mouse brain (adNSC)^14^ (Supplementary Fig. 1a), in which quiescence could be induced by exposure to BMP4 for 3 days^14^ (Supplementary Fig. 1b). To measure proteolysis in NSCs in vitro, we monitored three types of proteasomal peptidase activities (chymotrypsin-, trypsin-, and caspase-like) in whole-cell lysates prepared from proliferating and quiescent NSCs using three kinds of peptide substrates (Fig. 1a)^15^. In comparison with BMP-treated qNSCs, aNSCs exhibited small increases in the activities of chymotrypsin- and caspase-like proteases (Fig. 1a), which were completely inhibited by epoxomicin, a highly specific inhibitor of the proteasome (PI, Fig. 1a). Notably, qNSCs exhibited much higher trypsin-like activity than aNSCs and fibroblasts (C3H10T1/2 cells); this activity was not affected by epoxomicin. Unexpectedly, the trypsin-like activity was completely inhibited by cathepsin inhibitor I (CI, Fig. 1a), which is a specific inhibitor of the lysosomal proteases: papain and cathepsins B, L, and S. This result suggests that lysosomal activity was elevated in qNSCs. Consistent with this result, mRNA levels of lysosomal factors including cathepsins (CtsA, CtsB, and CtsF) and Lamp1, a lysosomal membrane protein, increased in NSCs upon entry into the quiescent state (Fig. 1b). Immunostaining of Lamp1 revealed that qNSCs contained more lysosomes in the cytoplasm than aNSCs (Fig. 1c), which was also detected in other NSCs, NS5 cells (Fig. 1d), and adult NSCs from the SVZ and DG (Fig. 1e). Furthermore, cathepsin activity measured by Magic Red staining was significantly higher in qNSCs than in aNSCs (Fig. 1c, f). These results suggest that elevated lysosomal activity might be important for proteolysis in qNSCs.

Next we investigated the transcription factor TFEB, a master regulator of lysosomal biogenesis^16^ in BMP-treated qNSCs. TFEB was expressed at higher levels after BMP exposure, concomitant with loss of cyclin D1, a G1/S cyclin (Fig. 1b, g, h). TFEB localized in the perinuclear region in the cytoplasm in all types of aNSCs, but increased in abundance and expanded its localization within qNSCs, in which some dots of TFEB were detected in nuclei (Fig. 1d, e, arrowheads). Western blotting revealed a decrease in the abundance of phosphorylated TFEB, an inactive form, and an increase in the level of active TFEB protein in qNSCs after BMP exposure (Fig. 1h). Consistent with this observation, monitoring of TFEB transcriptional activity using a luciferase reporter driven by the TFEB promoter^17^ revealed that luciferase expression was higher in qNSCs than in aNSCs (Fig. 1i). We next examined autophagic activity, because TFEB is involved in expression of numerous autophagy genes^18^. Consistent with this, NSCs expressed higher levels of LC3-II protein, a key component of autophagosomes and a marker of autophagy activation, after BMP exposure (Fig. 1g). Moreover, an LC3 turnover assay revealed higher levels of LC3-II in qNSCs than in aNSCs, even after lysosomal inhibition (Supplementary Fig. 1c), indicating that the increase in LC3-II was due not to reduced lysosomal activity, but rather to enhanced autophagy in qNSCs^19,20^. Together, these results indicate that autophagy/lysosomal degradation is strongly induced when aNSCs enter the quiescent state under TFEB activation after BMP exposure in vitro.

### Lysosomal inhibition allows qNSCs to proliferate

**Fig. 2:**
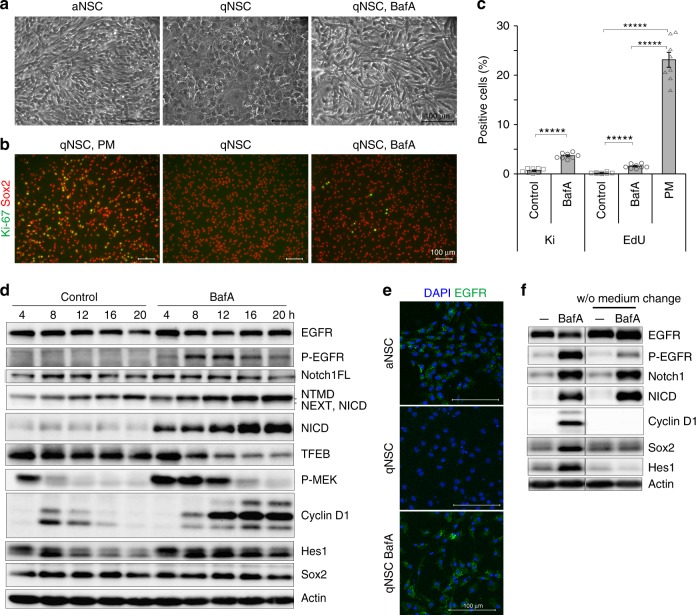
Lysosomal inhibition to qNSCs. **a** Bright field view of NSCs; bipolar proliferating NSCs (aNSC), flat qNSCs (qNSC), and bipolar qNSCs treated with 20 nM bafilomycin A (qNSC, BafA). **b** Immunocytochemistry of Ki-67. Representative view of NSCs immunostained for Ki-67 (green) and Sox2 (red). qNSCs were incubated in proliferation medium containing EGF (qNSC, PM), quiescence medium (qNSC), or quiescence medium containing BafA (qNSC, BafA) for 1 day, and then analyzed. **c** Percentage of Ki-67–positive (Ki) or EdU-incorporated (EdU) cells. Results are averages of eight samples of qNSCs (control), BafA-treated qNSCs (BafA), or qNSCs after incubation with proliferation medium (PM); *n* = 8. **d** Immunoblotting of qNSCs after incubation with qNSC medium (control) or qNSC medium containing BafA (20 nM) (BafA) for 4–20 h. **e** Immunocytochemistry of EGFR. BafA-treated qNSCs accumulated EGFR (green) in the cytoplasm. EGFR on the cell surface was not detected by immunostaining following membrane permeabilization. This might be responsible for the differences between the results of immunostainings and western blotting. **f** Effect of medium change. Samples on the left sides of blots were incubated in fresh quiescence medium, with or without BafA. In the w/o medium change samples, the medium in culture dishes was partially removed and added back with or without addition of BafA. Scale bars, 100 µm. Data represent means ± s.e.m. (*****P < 0.0001; Student’s *t*-test). Source data of immunoblots are provided as a Source Data file.

To evaluate the role of lysosomal degradation in qNSCs, we inhibited lysosomal function with bafilomycin A (BafA), a specific inhibitor of the vacuolar-type H(+)-ATPase (V-ATPase), which inhibits lysosomal enzymes and lysosomal membrane flux by increasing pH^21^. Long-term BafA treatment was toxic to NSCs, but upon treatment with a low concentration (20 nM) for 1 day, qNSCs converted their morphology from a flat to a bipolar shape, similar to that of aNSCs (Fig. 2a). We monitored cell proliferation by measuring expression of Ki-67, a proliferation marker, and incorporation of the thymidine analog EdU over 4 h. qNSCs did not express Ki-67, but replacement of quiescence medium with the proliferation medium (PM) containing EGF increased the proportion of Ki-67–positive cells, as previously reported^14^ (Fig. 2b). We detected some NSCs expressing Ki-67 after culture in BafA-containing quiescence medium (Fig. 2b, Supplementary Fig. 2a). The percentages of Ki-67– and EdU-positive cells significantly increased relative to those in control qNSCs (Fig. 2c). The increase in the proportion of EdU-positive cells after BafA treatment peaked at 20 nM BafA (Supplementary Fig. 2b). The elevation in the proportion of Ki-67–positive cells also appeared at a different cell density in the presence of BafA (Supplementary Fig. 2c).

Next, we analyzed the effects of BafA treatment on the EGF and Notch pathways, which play essential roles in the activation of NSCs. In qNSCs, the levels of EGFR, phospho-(P-)EGFR (the activated form), P-MEK (the activated form of MEK, a downstream target of EGFR signaling), and various forms of the Notch1 receptor (full-length precursor [Notch1 FL], processed mature form [NTMD], and activated form [NICD]; Supplementary Fig. 3a) were reduced when aNSCs entered a quiescent state in quiescence-inducing medium containing BMP (Fig. 1g). On the other hand, BafA-treated qNSCs continuously expressed P-EGFR and NICD from 4 h and cyclin D1 from 8 h onward after BafA treatment, and expressed P-MEK at a higher level for longer than the control (Fig. 2d, Supplementary Fig. 2d). Although medium change at time zero induced P-MEK at 4 h and caused a slight induction of cyclin D1 at 8 h in the control cells (Fig. 2d, Supplementary Fig. 2d), these inductions were not maintained in control cells at later time points, suggesting that sustained activation of MEK and cyclin D1 is specific to BafA treatment. Immunostaining for EGFR revealed that accumulation of cytoplasmic EGFR, which was not detected in qNSCs, persisted in BafA-treated cells even in the absence of EGF, as well as in aNSCs in EGF-containing proliferation medium (Fig. 2e). Accumulation of P-EGFR and NICD plateaued under treatment with 20 nM BafA for 4 h (Supplementary Fig. 2e), the same BafA concentration that was associated with the peak level of EdU-positive cells (Supplementary Fig. 2b). These data suggest that BafA induced accumulation of activated EGFR (P-EGFR) and activated NotchR (NICD), which in turn allowed cell-cycle reentry of qNSCs. Consistent with this idea, microarray analysis to monitor mRNA expression at 4 h after BafA treatment revealed upregulation of genes related to cell proliferation and elevated receptor activity in BafA-treated cells (Supplementary Fig. 2f). Expression of Notch and EGF signaling-related genes increased at 4 and 24 h after BafA treatment (Supplementary Data 1 and 2).

To assess the involvement of autophagy, we analyzed SAR405, a specific inhibitor for VPS34 and autophagosome formation^22^; however, SAR405 did not affect the levels of cyclin D1, P-EGFR, or NICD (Supplementary Fig. 2g). Recent studies demonstrated that V-ATPase is involved in endocytosis^23^ as well as endolysosomal trafficking^24^, and that BafA may inhibit the endoplasmic reticulum (ER) calcium ATPase^25^. However, inhibitors of clathrin-mediated endocytosis (Dynasore), the ER calcium ATPase (thapsigargin [Tg]), and microtubule organization (nocodazole [Noc]) had weaker effects on cyclin D1 and P-EGFR than BafA (Supplementary Fig. 2h), and thapsigargin induced abnormal cleavage of Notch1 receptor. Together, these results indicate that the lysosomal defect, but not autophagy- or endocytosis-related defect, is responsible for BafA treatment induced aberrant increases in the levels of both P-EGFR and NICD in qNSCs, which are prone to induce cyclin D1 expression and undergo the cell-cycle reactivation. We also analyzed the effect of changing the medium. BafA treatment without changing the medium dramatically decreased the levels of cyclin D1 and P-EGFR, but did not change the level of NICD (Fig. 2f), suggesting that both lysosomal inhibition by BafA treatment and EGFR activation by new medium are crucial for reactivation of qNSCs.

### Regulation of EGFR and Notch signaling in qNSCs

**Fig. 3:**
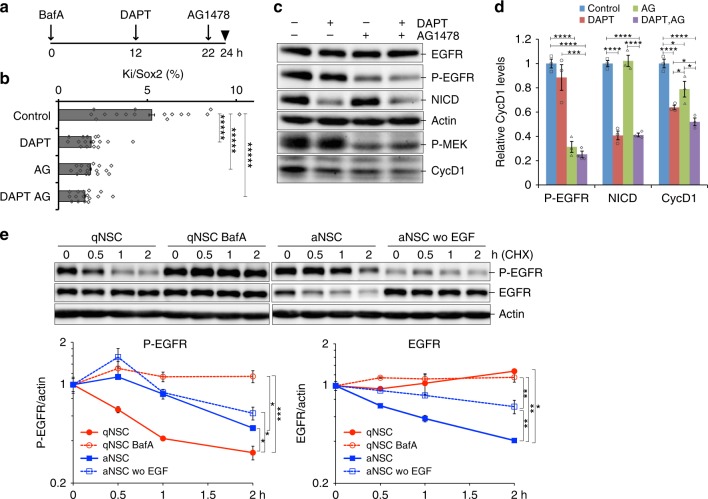
Regulation of EGF and Notch signaling in qNSCs with defects in lysosomal function. **a** Cells were incubated for the indicated times with inhibitors of Notch (DAPT) and EGF signaling (AG1478) after BafA treatment at *t* = 0 h. qNSCs were cultured with 10 µM DAPT from *t* = 12 h (arrow), 5 µM AG1478 from *t* = 22 h (arrow), or both inhibitors in the presence of 20 nM BafA, and then analyzed at *t* = 24 h (arrowhead). DAPT required a longer incubation time than AG1478 to reduce the NICD level. **b** Percentage of Ki-67–positive cells in Sox2-positive cells. **c** Representative immunoblots of qNSCs after incubation with inhibitors in the presence of BafA. **d** Relative protein levels of P-EGFR, NICD, and CycD1 in (**c**), normalized against β-actin. **e** Protein stability of EGFR (*n* = 2). BafA was added 4 h before all assays in lysosomal-inhibited samples. Activated EGFR (P-EGFR) and total EGFR (EGFR) in immunoblots were quantified and plotted (lower panels). Left panel: qNSCs degrade P-EGFR (red closed circles) more rapidly than aNSCs (blue closed squares), but the protein was stabilized in the presence of BafA (red open circles). Total EGFR degradation depended on the presence of EGF (blue squares). Data represent means ± s.e.m. (*P *<* 0.05, **P *<* 0.01, ***P *<* 0.005, ****P *<* 0.001, ***** *P* *<* 0.0001; Student’s *t*-test). Source data of immunoblots are provided as a Source Data file.

To determine whether activation of both EGFR and Notch signaling is required to induce qNSC reactivation under BafA treatment, we treated cells with DAPT, a gamma-secretase inhibitor, to block Notch signaling, as well as with AG1478, an EGFR tyrosine kinase inhibitor (Fig. 3a). We monitored cell proliferation by Ki-67 and cyclin D1 expression following inhibitor treatment, and detected a significant decrease in the levels of these proliferation markers; comparable effects were observed upon treatment with DAPT, AG1478, or the combination of both inhibitors (Fig. 3b–d). These results indicate that both EGFR and Notch signaling pathways are indispensable for reactivation of qNSCs after lysosomal inhibition. Previous in vivo studies revealed that Notch activation is required for maintenance of the quiescent state of NSCs: proliferating NSCs and progenitors are transiently upregulated and then exhausted by inhibition of Notch signaling^26^, whereas continuous NICD expression promotes NSC proliferation^27^. These opposing functions of Notch might be dependent on expression mode or environmental conditions. In the case of cultured NSCs, Notch signaling might promote NSC proliferation: NICD was highly upregulated in proliferating aNSCs (Fig. 1g), and inhibition of Notch prevented the reactivation of qNSCs after BafA treatment (Fig. 3b–d).

We next investigated how NSCs regulate the stability of these receptors in the quiescent and proliferating states by measuring protein stability following treatment with the translational inhibitor cycloheximide. In the case of BafA-treated samples, we collected samples 4 h after BafA addition, because longer incubation with BafA can cause effects independent of lysosomal inhibition^28^. The results revealed that activated EGFR (P-EGFR) was degraded more rapidly in qNSCs than in aNSCs (Fig. 3e). Removal of EGF from the culture medium of aNSCs did not significantly alter the rate of degradation (Fig. 3e). BafA dramatically stabilized P-EGFR in qNSCs (Fig. 3e), although the level of P-EGFR was lower in qNSCs than in aNSCs (Supplementary Fig. 2i, j). Total EGFR was degraded more rapidly in aNSCs in the presence of EGF than in qNSCs (Fig. 3e). Together, these results lead to several conclusions. First, overall degradation of EGFR is limited in the absence of EGF; second, consequently, EGFR is activatable even in qNSCs at a low level (mathematically simulated in Supplementary Notes). Third, activated EGFR in qNSCs is rapidly degraded via the lysosomal degradation pathway.

Next, we examined the protein stability of Notch1 receptor, and found that NICD was degraded at the same rate in proliferating and quiescent NSCs (Supplementary Fig. 3b). Mature (NTMD) and full-length Notch1 receptors were degraded at similar rates in aNSCs and qNSCs (Supplementary Fig. 3c, d). BafA completely stabilized both NICD and NTMD but did not affect the cleavage of full-length Notch1 (Supplementary Fig. 3b–d). These results demonstrate that lysosomal inhibition stabilizes membrane-anchored NTMD, potentially resulting in the abnormal increase of NICD in BafA-treated cells.

### Enhanced lysosomes maintain qNSCs in brain

**Fig. 4:**
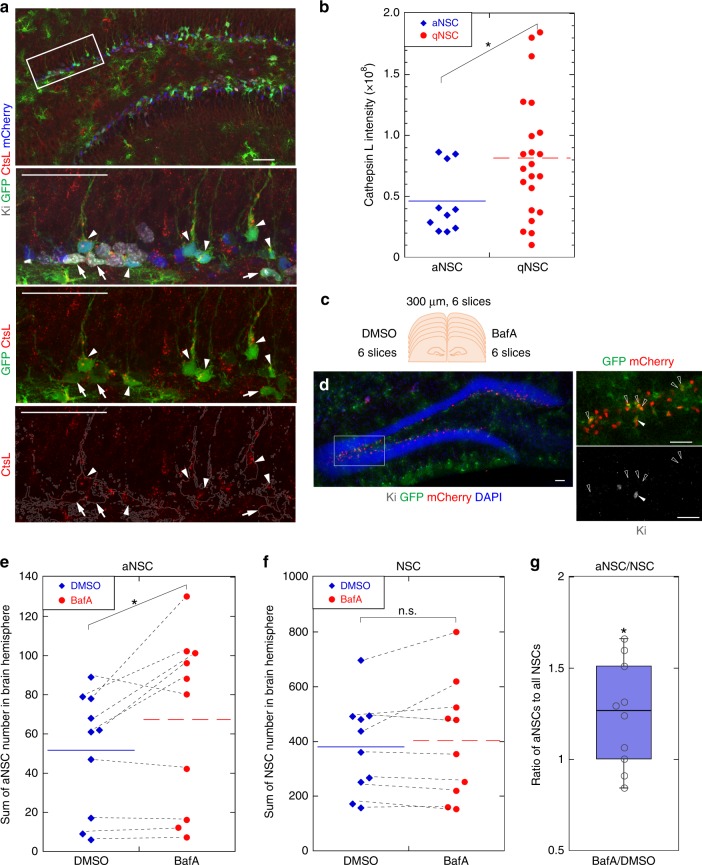
Increased lysosome abundance in quiescent NSCs and lysosomal inhibition in the tissue. **a** Immunohistochemistry of cathepsin L in the DG of GFAP-GFP;Nestin-NLS-mCherry mice. Proliferating NSCs expressed Ki-67 (gray), GFP (green), and mCherry (blue), but qNSCs were Ki-67–negative. The selected region indicated by a white square is enlarged below. Proliferating NSCs (arrows) exhibited lower cathepsin L (CtsL) staining than qNSCs (arrowheads). Cell shapes are indicated by gray lines in the lower panel. **b** Quantified values of cathepsin L intensity in individual NSCs in the DG (*n* = 32 in total). **c** Experimental design of brain slice cultures. **d** Representative images of the DG stained with DAPI (blue), GFP (green), mCherry (red), and Ki (gray) at a single plane of the brain slices after the slice culture. Open and closed arrowheads indicate qNSCs and aNSCs, respectively. **e**, **f** Numbers of Ki-67, GFP, and mCherry triple-positive cells (aNSCs (**e**)), and GFP and mCherry double-positive cells (NSCs (**f**)) in the SGZ of brain slices from 6-month-old mice cultured with DMSO (vehicle) or BafA. Dot plots show the sums of numbers of aNSCs (**e**) and NSCs (**f**) in individual hemispheres. Average values in each data set are shown by cross bars, and dotted lines link results from the same individuals. **g** Box plot showing the proportion of aNSCs in NSCs (center line, median; box limits, upper and lower quartiles; whiskers, minimum and maximum). Corresponding data points was plotted as dots. BafA increased the proportion of aNSCs to total NSCs in the DG. Scale bars, 50 µm. Data represent means ± s.e.m. (*P *<* 0.05; n.s., not significant; Student’s *t*-test, *n* = 10 for **e**–**g**).

We next tried to address lysosomal functions in NSCs in the juvenile and adult mouse brain. We monitored in vivo expression of the lysosomal proteins cathepsin B, cathepsin L, and Lamp1 by immunohistochemistry of the DG and the SVZ of the lateral ventricle. To identify NSCs in brain tissues, we used GFAP-GFP;Nestin-NLS-mCherry double-transgenic mice^29–32^. In this transgenic mouse line, NSCs were labeled with GFP and nuclear-localized mCherry signals expressed under the control of mouse GFAP and Nestin promoters, respectively, and aNSCs were distinguished by Ki-67 staining. We compared the immunostaining of lysosomal enzymes between qNSCs and aNSCs in the DG and SVZ in young and adult mice (2-week-old and 6-month-old). Cathepsins and Lamp1 were stained as cytoplasmic dots in both types of NSCs. The dots were more dense and abundant in qNSCs (arrowheads) than in aNSCs (arrows) in both the DG and SVZ of 2-week-old mice (Fig. 4a, Supplementary Fig. 4a–d). In 6-month-old mice, the morphology of qNSCs in the DG was different from that in 2-week-old mice, but lysosomes were distributed similarly to those in young mice and condensed in qNSCs (Supplementary Fig. 4e), especially in the basal region of long processes of qNSCs at both ages (Fig. 4a, Supplementary Fig. 4a–c, e). Lysosomes were significantly more abundant in qNSCs than in aNSCs (Fig. 4b). These observations are consistent with our data obtained in vitro (Fig. 1c–f).

Acute BafA injection into the DG of mouse brain resulted in severe lysosomal damage around the injected regions. Therefore, in order to analyze the effect of lysosomal inhibition by BafA in the brain tissue, we pursued ex vivo approach using slice cultures of GFAP-GFP;Nestin-NLS-mCherry double-transgenic mice. Slices, including the whole DG from individual hemispheres, were incubated in BafA-containing or control culture medium for 15 h (Fig. 4c). Total NSCs (GFP^+^ mCherry^+^ [double-positive] cells) and aNSCs (GFP^+^ mCherry^+^ Ki-67^+^ [triple-positive] cells) were counted in the SGZ of all brain slices (Fig. 4d). For quantitative analyses, we eliminated cells with sparse GFP expression, which might include GFAP-negative intermediate progenitors, reported previously^33^. BafA-treated slices contained significantly higher numbers of aNSCs than controls (Fig. 4e). BafA treatment did not affect the total number of NSCs over the course of the experimental period (Fig. 4f), but it did significantly increase the proportion of aNSCs in total NSCs relative to the control (Fig. 4g). Culturing slices upregulated genes associated with acute injury responses, but this effect was comparable between control and BafA-treated slices after culture (Supplementary Fig. 4f). These results indicate that lysosomes are enriched in qNSCs in vivo as well as in vitro, and that inhibition of lysosomal function can reactivate NSCs in the SGZ.

### Role of lysosomal enhancement by TFEB activation in NSCs

**Fig. 5:**
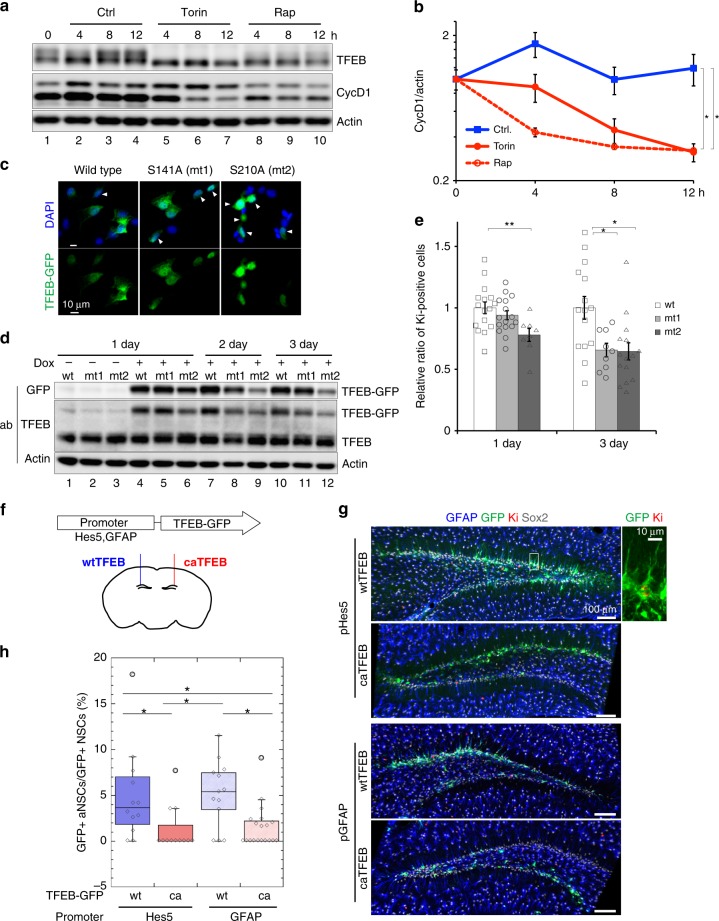
Effects of activated lysosomes on aNSC proliferation. **a** Pharmacological activation of lysosomes in aNSCs. Representative immunoblots for TFEB, cyclin D1, and actin after incubation with DMSO (Ctrl), 1 nM Torin-1 (Torin), or 10 µM rapamycin (Rap) for 0, 4, 8, or 12 h. **b** Quantification of cyclin D1 shown in **a**. **c** Immunocytochemistory of TFEB-GFP (green) in the wild type or cells expressing two constitutive-active mutants (mt1: S141A; mt2: S210A) with DAPI (blue) under the control of the tet-on inducible system on 1 day. White arrowheads indicate nuclear localization of TFEB-GFP. **d** Representative immunoblots for GFP, TFEB, and actin in the presence or absence of doxycycline (dox). **e** Proportions of Ki-67–positive cells in TFEB-GFP–expressing cells on 1 day and 3 day. Values are shown relative to wild-type TFEB-GFP. Scale bars, 10 µm. Data represent means ± s.e.m. (*P < 0.05 and **P < 0.01; Student’s *t-*test for **b** and **e**). **f** Schematic diagram of the delivery of lentiviral vectors encoding TFEB-GFP driven by the *Hes5* or *GFAP* promoter into the DG. **g** Representative images from 50-µm-thick FF-IHC sections. Mice were fixed 3 days after virus injection. For counting, every sixth slice through the whole DG was immunostained with GFAP, GFP, Ki-67, and Sox2 antibodies. **h** Percentages of Ki-67^+^ aNSCs among total GFP^+^ NSCs (box plot: center line, median; box limits, upper and lower quartiles; whiskers, minimum and maximum). Corresponding data points were plotted as open diamonds. Cells were counted in the SGZ of around six slices for each condition per mouse, using the Imaris software. Data represent means ± s.e.m. (*P < 0.05; Tukey’s test. *n* = 5). Source data of immunoblots are provided as a Source Data file.

To investigate the role of lysosomal activation in NSC proliferation, we activated lysosomes by two methods: first, pharmacologically, and second, with constitutively active mutants of TFEB. TFEB is inactivated by phosphorylation mediated by target of rapamycin complex 1 (mTORC1), a conserved serine/threonine kinase complex^34^. We analyzed cell proliferation after treatment with the mTORC1 inhibitors rapamycin and Torin-1. Phosphorylated TFEB, the inactive form, disappeared by 4 h (Fig. 5a, lanes 5 and 8), and the levels of cyclin D1 were reduced by incubation with the inhibitors (Fig. 5a, lanes 6–10, and Fig. 5b). mTORC1 is involved in downstream EGF signaling in neural progenitor cells^35,36^. To verify the involvement of TFEB in cell proliferation, we analyzed phospho-deficient mutants of TFEB, TFEB S141A (mt1), and TFEB S210A (mt2), which are constitutively active. Ser141 and Ser210 in mouse TFEB are conserved residues previously identified as mTORC1 targets^34^. The mutants, as well as wild-type TFEB, were expressed as GFP fusions. We first continuously expressed these mutants under the control of the EF promoter in a construct delivered by a lentiviral vector, but the number of mutant TFEB-GFP–expressing cells was dramatically decreased in culture after several passages. Thus, these TFEB-GFPs were expressed under the control of a doxycycline (dox)-inducible promoter after transduction with lentiviral vectors (Fig. 5c–e, Supplementary Fig. 5). TFEB S141A and S210A mutants localized in the nucleus to greater extent than wild-type TFEB (Fig. 5c) as reported previously^34^. Expression levels of mutant TFEB-GFPs were comparable to those of wild-type TFEB-GFP and endogenous TFEB on 1 day (Fig. 5d, lanes 4–6), but gradually decreased relative to the wild type on days 2 and 3 (Fig. 5d, lanes 7–12). This result suggests that the number of cells expressing mutant TFEB-GFPs relative to that of non-infected cells decreased in culture, similar to the observation using the EF promoter as mentioned above. Consistent with this observation, the proportion of TFEB-GFP–expressing cells that were Ki-67–positive decreased in TFEB mutants (Fig. 5e). Live-cell imaging confirmed significant decreases in cell division in NSCs expressing the TFEB S210A mutant (Supplementary Fig. 5a, b, Supplementary Movies 1 and 2). These results indicate that TFEB activation decreases NSC proliferation in vitro.

To activate lysosomes via TFEB activation in NSCs of the adult brain, we stereotactically injected lentiviral vectors encoding wild type and constitutively active mutant (S210A) TFEB (wtTFEB and caTFEB, respectively) under the control of the *Hes5* and *Gfap* promoters into the DG of the adult mouse brain^37^ (Fig. 5f–h). Both promoters enabled expression of TFEB-GFP in the SGZ (Fig. 5g). NSCs expressing TFEB-GFP were identified as GFAP-, GFP-, and Sox2-triple–positive cells in the SGZ; TFEB-GFP–positive aNSCs were identified as GFAP-, GFP-, Ki-67–, and Sox2-quadruple–positive cells (Fig. 5g). caTFEB significantly decreased the number of Ki-67–positive aNSCs in the DG (Fig. 5h). These observations indicate that TFEB activation decreases proliferation and induces quiescence in NSCs of the SGZ in the adult brain.

### TFEB-knockout increases the abundance of active NSCs

**Fig. 6:**
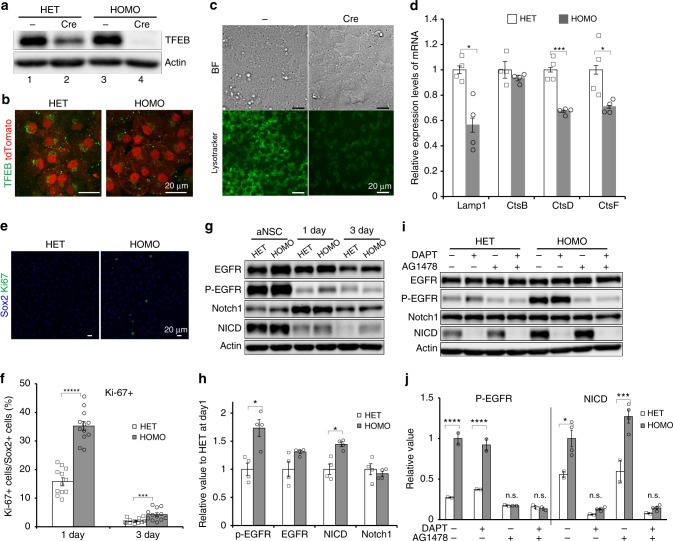
Reduction in lysosomal activity and slow induction of quiescence by *Tfeb*-knockout. **a** Immunoblot of TFEB in *Tfeb*-KO NSCs (HOMO) and control NSCs (HET) in the quiescent state in vitro. The parental cells (without Cre excision) and tdTomato-positive cells (purified by cell sorting after Cre excision) were analyzed after culture in quiescence medium. **b** Immunocytochemistry of TFEB (green) in *Tfeb*-KO qNSCs and control qNSCs. Cre-excised cells were labeled with tdTomato (red). **c** Bright field and LysoTracker-green staining in *Tfeb*-KO and parental control NSCs in the quiescent state. **d** mRNA expression of lysosomal genes in *Tfeb*-KO NSCs (gray bars) and controls (white bars) in the quiescent state (*n* = 4). **e** Representative immunostaining 3 days after BMP exposure. **f** Proliferation of *Tfeb*-KO (gray bars) and control NSCs (white bars) in quiescence medium. Cells were fixed, stained with Sox2 (blue) and Ki-67 (green) at 1 and 3 days following BMP exposure, and analyzed (*n* = 12). **g** Representative immunoblot for EGFR, Notch1, and their active forms in *Tfeb*-KO and control in proliferation medium (aNSC) and on 1 and 3 days in quiescence medium. **h** Relative quantification of P-EGFR, EGFR, NICD, and Notch1 on 1 day after BMP exposure in **g** (*n* = 4). **i** Representative immunoblots after incubation with Notch (DAPT) and EGFR inhibitors (AG1478) on 1 day after BMP exposure. Inhibitors were incubated same as Fig. 3a. **j** Relative quantification of P-EGFR and NICD in **i** (*n* = 2, 4). Data represent means ± s.e.m. (*P *<* 0.05, ***P *<* 0.005, ****P < 0.001, *****P < 0.0001; Student’s *t*-test for **d** and **f**, Tukey’s test for **h** and **j**, n.s. not significant). Scale bars, 20  µm. Source data of immunoblots are provided as a Source Data file.

To confirm the role of TFEB in qNSCs in vitro, we deleted the *Tfeb* gene in NSCs derived from *Tfeb*-flox mice^34^. For this purpose, we used the Rosa-CAG–based tdTomato reporter line (Ai14), in which NSCs and their progeny are labeled with tdTomato following Cre excision. We generated NSCs from embryos after crossing *Tfeb*(flox/flox) and *Tfeb*(flox/+);Ai14(+/+) mice, yielding heterozygote [*Tfeb*(flox/+);Ai14] and homozygote [*Tfeb*(flox/flox);Ai14] NSCs. The *Tfeb-flox* gene was excised by transient Cre expression under control of the CAG promoter, introduced by lipofection, and then tdTomato-positive cells from homozygotes (HOMO) and heterozygotes (HET) (used as a control) were purified by cell sorting. These cells were cultured in quiescence-inducing medium to analyze the effect of *Tfeb*-knockout (KO) on the quiescent state. TFEB protein was clearly depleted in *Tfeb*-KO NSCs (HOMO) relative to the parental cells (Fig. 6a, lanes 3 and 4) and control NSCs (HET) (Fig. 6a, lanes 2 and 4, and Fig. 6b). *Tfeb*-KO NSCs stained with LysoTracker exhibited weaker signals than the parental control (Fig. 6c), indicating that *Tfeb*-KO reduced lysosomes in qNSCs. Expression of multiple lysosomal genes was significantly reduced in *Tfeb*-KO NSCs (Fig. 6d). *Tfeb*-KO increased the number of Ki-67–positive NSCs compared to the control after the quiescence induction (Fig. 6e, f), indicating that *Tfeb*-KO NSCs entered quiescence more slowly than the control. Consistent with this, active forms of EGFR and Notch1, which accumulated upon lysosomal inhibition by BafA in qNSCs (Fig. 2d), were present at higher levels in *Tfeb*-KO NSCs than in controls (Fig. 6g, h). These increases in the levels of P-EGFR and NICD in *Tfeb*-KO were abolished by inhibitors of EGFR and Notch, respectively (Fig. 6i, j). These results indicate that the reduction in lysosomal activity due to *Tfeb* deletion exhibited slower adaptation of NSCs to be quiescent in vitro.

**Fig. 7:**
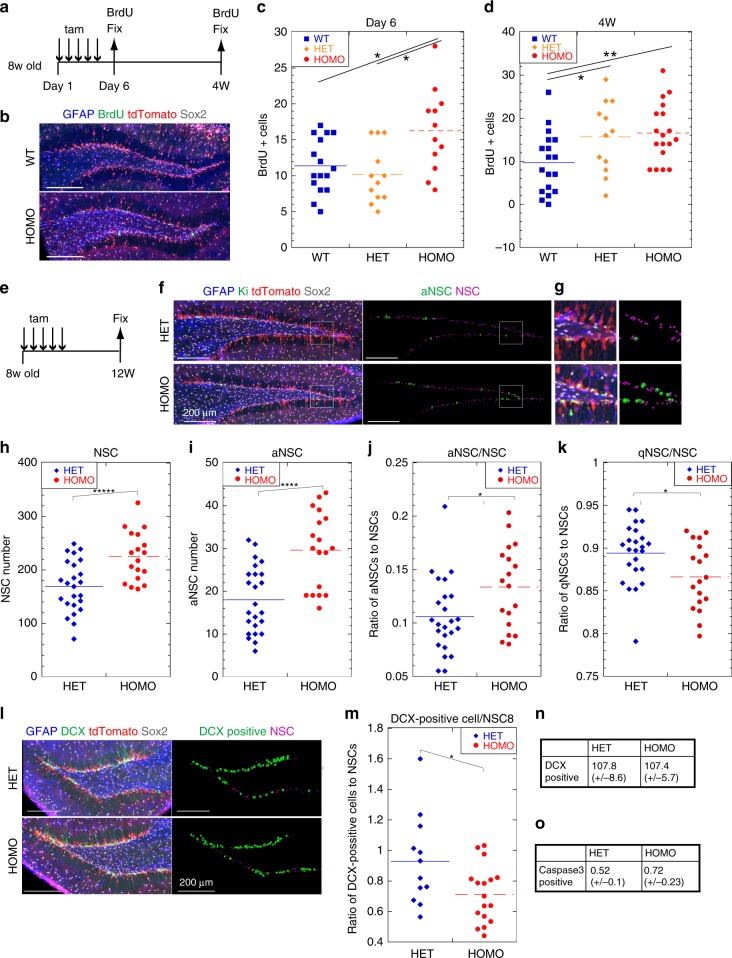
Elevated number of proliferating NSCs in the SGZ of *Tfeb*-cKO mice. **a** aNSCs in *Tfeb*-cKO mice and control mice were labeled with BrdU for 2 h before fixation on day 6 or at 4 weeks (4 W) after tamoxifen administration. **b** Representative images of the DG in wild type (WT) and cKO mice (HOMO) at 4 weeks, immunostained with GFAP (blue), BrdU (green), tdTomat (red), and Sox2 (gray). **c**, **d** Dot plots showing the number of BrdU-positive aNSCs in the DG of wild type (WT), heterozygote (HET), and homozygote *Tfeb*-cKO (HOMO) mice. Cells were counted in each image from 50-µm-thick FF-IHC sections throughout the DG. Four and six slices from each brain were counted on day 6 (**c**) and at 4 weeks (**d**), respectively (*n* = 4 [WT], 3 [HET], and 3 [HOMO] for day 6; *n* = 3 [WT], 2 [HET], and 3 [HOMO] for 4 weeks). **e** *Tfeb*-cKO mice (HOMO) and control mice (HET) were analyzed 12 weeks after tamoxifen administration. **f** Representative images of the DG from the control (HET) and *Tfeb*-cKO mice (HOMO), immunostained for GFAP (blue), Ki-67 (green), and Sox2 (gray) and processed using the Imaris software (right panels). Processed images were used to count NSCs (magenta spots) and aNSCs (green spots) using the Imaris software. **g** Enlarged view of white squares in **f**. **h**, **i** Dot plots showing the numbers of total NSCs and aNSCs in slices. Six slices from each brain were counted (*n* = 4 [HET] and 3 [HOMO]). **j**, **k** Proportions of aNSCs to NSCs (**j**) and qNSCs to NSCs (**k**), calculated from (**h**) and (**i**). **l** Representative images of the DG, immunostained as indicated (left panels) and processed (right panels) to count DCX-positive cells (green spots) and NSCs (magenta spots). **m** Ratio of DCX-positive cells to NSCs. Six slices from each brain were counted (*n* = 2 [HET] and 3 [HOMO]). **n**, **o** Average number of cells double-positive for tdTomato and DCX (**n**) or tdTomato and cleaved-caspase-3 (**o**) in the DG in slices. Data represent means ± s.e.m. (*P *<* 0.05, **P *<* 0.01, ****P *<* 0.001, *****P < 0.0001; Student’s *t*-test). Scale bars, 200 µm.

Next, we investigated the conditional knockout of *Tfeb* (*Tfeb* cKO) in vivo using GLAST-Cre-ERT2 mice to induce *Tfeb* deletion specifically in adult NSCs after tamoxifen administration^38,39^. We injected tamoxifen into 8-week-old mice and analyzed tdTomato-positive cells in the DG of *Tfeb*-cKO and the control mice on day 6, and at 4 weeks and 12 weeks after the injection (Fig. 7a, e). In the SGZ of *Tfeb*-cKO mice, TFEB was depleted in tdTomato-positive cells after tamoxifen administration. In brain slices throughout the DG along the rostrocaudal axis, we identified tdTomato-positive NSCs in the 3D reconstitution by immunostaining for Sox2 and GFAP. To monitor proliferating cells, proliferating NSCs were labeled by BrdU for 2 h before fixation, and then counted in brain slices in wild type (WT), heterozygote (HET), and homozygote mice (HOMO) on day 6 or at 4 weeks (Fig. 7b). BrdU-labeled aNSCs were significantly higher in *Tfeb*-cKO HOMO than in controls on day 6 (Fig. 7c). HET mice, as well as *Tfeb*-cKO HOMO mice, had more aNSCs than wild type at 4 weeks (Fig. 7d), suggesting that reduced TFEB amount might gradually affect NSC activation between day 6 and 4 weeks in vivo. At 12 weeks after tamoxifen administration, both NSCs and aNSCs, identified as Ki-positive NSCs, were significantly more abundant in *Tfeb*-cKO HOMO mice than in control HET mice (Fig. 7f, g, h, i). Importantly, among all NSCs, *Tfeb*-cKO HOMO mice had a higher proportion of aNSCs and a lower proportion of qNSCs than control HET mice (Fig. 7j, k). To determine whether TFEB depletion in NSCs increased the number of newly generated neurons, we counted tdTomato and DCX double-positive cells in the DG (Fig. 7l). However, DCX-positive cells were comparably abundant in *Tfeb*-cKO HOMO and control HET mice (Fig. 7n), suggesting that activation of NSCs did not increase neurogenesis in *Tfeb*-cKO mice. The proportion of DCX-positive cells to NSCs was significantly lower in cKO HOMO mice than in control HET mice (Fig. 7m). Cells positive for cleaved-caspase-3, a marker of apoptosis, were scarce in all samples (Fig. 7o). Consistent with this in vivo result, neuronal differentiation of *Tfeb*-KO HOMO NSCs was slightly delayed in vitro (Supplementary Fig. 6a, b). During differentiation, nuclear localization of TFEB was detected after 1 day of culture in differentiation medium (Supplementary Fig. 6c), implying another role of TFEB in the early neuronal differentiation of NSCs. To analyze the effect of membrane receptor after TFEB depletion in vivo, we assessed the levels of the Notch1 receptor by immunostaining (Supplementary Fig. 7a–c). Levels of the Notch1 receptor were slightly elevated in the SGZ of adult *Tfeb*-cKO mice; however, there was a wide variety relative to wild type (Supplementary Fig. 7c). Alternatively, we analyzed postnatal *Tfeb*-cKO mice at P0 stage (Supplementary Fig. 7d, e, see Methods). In P0 mice, levels of the Notch1 protein intensity significantly increased in the DG of *Tfeb*-cKO mice than in wild-type mice (Supplementary Fig. 7f). To address the expression profiles of Notch1 and EGFR, we examined these receptors of the DG in *Tfeb*-cKO and the control adult mice by western blotting. The results revealed significant increases in the active forms of EGFR and Notch1 in *Tfeb*-cKO mice relative to control mice (Supplementary Fig. 8a, b). Taken together, our results show that the reduction in lysosomal activity due to TFEB depletion increases activation of NSCs in the DG along with accumulation of activated membrane receptors, resulting in an increase in the number of aNSCs, but does not enhance their differentiation.

## Discussion

Our findings demonstrate that lysosomal activity is higher in qNSCs than in aNSCs, and that lysosomal inhibition by both chemical and genetic approaches increases the proportion of aNSCs among all NSCs both in the DG of mouse brain, as well as in cultured qNSCs with higher levels of membrane receptors (Supplementary Fig. 9). These results suggest that enhanced lysosomal function is important for adaptation and maintenance of qNSCs in the adult mouse brain. Given that activated EGFR was rapidly degraded in qNSCs in vitro, enrichment of lysosomes might serve to increase the chance that endosomal vesicles containing membrane receptors will fuse to lysosomes, thereby reducing the time required for signal transduction from the endosome via endolysosomal fusion. Other regulatory events, such as the initiation of endocytosis, endosomal traffic, and recycling, may also contribute to the selective reduction of activated EGFR in qNSCs. EGFR expression persisted at low levels in qNSCs^14,40^, potentially enabling these cells to remain in a poised state capable of responding to activation signals. On the other hand, degradation of Notch1 receptor was similar in qNSCs and aNSCs, implying that the degradation rate of Notch receptor is independent of the enhanced lysosomal activity in NSCs. Medium change was another crucial inducer of qNSCs reactivation, suggesting that cooperative functions of BafA and a factor in fresh medium are required for qNSC reactivation in vitro. Because the levels of phosphorylated FGF receptor 1 (FGFR1), as well as P-EGFR, increased by BafA in new quiescent medium, FGF is a strong candidate for such a factor.

In addition, we detected TFEB activation in qNSCs (Supplementary Fig. 9). *Tfeb* gene has been identified as a downstream targets of p-SMAD1/5 in the BMP signaling pathway in hair follicle stem cells^41^, implying that BMP signaling might directly activate TFEB transcription. In this study, we observed that TFEB protein was dephosphorylated after BMP exposure in adult NSCs, suggesting that post-translational regulation of TFEB, such as dephosphorylation by inhibition of mTOR complex 1 (mTORC1), might lead to TFEB activation in qNSCs^34^. Once activated, TFEB can increase transcription of TFEB itself via an auto-regulatory loop, as well as the expression of most lysosomal genes under the coordinated lysosomal expression and regulation (CLEAR) gene network. This idea is consistent with our findings that TFEB activation by chemical activatiors and consitiutively active mutants prevented aNSC proliferation in vitro (Fig. 5a–e). Regarding cell differentiation, roles of the microphthalmia family (MiTF, TFEB, TFE3, and TFEC) of transcription factors have been reported for TFEB in osteoclast differentiation^42^ and TFE3 in macrophage differentiation^43^. Recent studies also showed that TFE3, like TFEB, regulates lysosomal gene expression^44^, suggesting that TFE3 might cooperate in maintenance of qNSCs. For quality control in NSCs, autophagy might contribute to removal of abnormal compartments such as damaged protein aggregates and mitochondria, which are sources of oxidative stresses; this has been reported in mice harboring a conditional knockout of the autophagy-related gene FIP200 under the control of the hGFAP-Cre driver^45,46^. After reactivation of qNSCs, the abundant lysosomes might be diluted after cell division or secreted along with their lysosomal contents to the cell surface, thereby inducing environmental changes that further contribute to NSC activation.

Our results obtained from *Tfeb*-KO NSCs in vitro revealed that aNSCs exhibited slower induction of the quiescent state than the control (Fig. 6), and that *Tfeb* deletion in NSCs increased the number of aNSCs in the DG in vivo (Fig. 7). These results indicate that reduced lysosomal activity increases the abundance of aNSCs in vitro and in vivo. Especially, in vivo, the increases in the abundance of aNSCs due to TFEB depletion might progress slowly, along with some unknown compensation machinery in qNSCs. On the other hand, given that the ratio of DCX-positive cells to NSCs was reduced in *Tfeb*-cKO mice in vivo and *Tfeb*-KO NSCs in vitro, TFEB might also be involved in differentiation from NSCs. Further analyses of different time periods following *Tfeb* deletion from the embryonic stage or earlier adulthood might reveal the role of lysosomes in NSC differentiation and brain formation. Moreover, samples from the later time period, after *Tfeb* deletion, could reveal the role of lysosome in aged brain. Together, our findings demonstrate that lysosomal function contributes to maintenance of NSC quiescence.

## Methods

### Mice

*Tfeb*-flox mice^34^ were crossed with Ai14 reporter mice (Rosa-CAG-LSL-tdTomato-WPRE, stock 007908, The Jackson Laboratory) or GLAST-CreERT2 mice (a kind gift from M. Götz^38,39^, and the offspring were crossed to obtain GLAST-CreERT2;Ai14;*Tfeb*(flox/flox), GLAST-CreERT2;Ai14;*Tfeb*(flox/+) and GLAST-CreERT2;Ai14 mice. Two milligrams tamoxifen (Tam; Sigma, 20 mg/ml in corn oil) was intraperitoneally injected into adult mice (8-week-old) by twice injections a day (or once injection a day for BrdU experiments) for five consecutive days to induce *Tfeb* deletion and tdTomato expression. Tam-injected mice were housed in individual cages and sacrificed at the times indicated in each experiment. For the BrdU incorporation assay, 200 mg/kg BrdU (BrdU; Sigma, 10 mg/ml in a saline solution) was intraperitoneally injected 2 h before fixation. GFAP-GFP;Nestin-NLS-mCherry double-transgenic mice were obtained by crossing GFAP-GFP reporter mice^29^ with Nestin-NLS-mCherry mice^32^. Nestin-CreERT2 mice^47^ were crossed with *Tfeb*-flox mice (Supplementary Methods). All mice were of the C57BL/6 background. Mice were maintained in our animal facility and housed in a 12:12 hour light–dark cycle. Animal care and experiments were conducted in accordance with the guidelines of the animal experiment committee of Kyoto University. We have complied with all relevant ethical regulations for animal testing and research.

### Cell culture and induction of quiescent state

C3H10T1/2 mouse fibroblast cells (ATCC) were grown in Dulbecco’s modified Eagle’s medium (DMEM; Nacalai Tesque) supplemented with 10% fetal bovine serum (FBS) and penicillin–streptomycin (P/S; Nacalai Tesque)^48^. NSCs were passaged in proliferation medium [20 ng/ml EGF (Wako), 20 ng/ml bFGF (Wako), P/S, and N-2 Plus media supplement (R&D Systems) in DMEM/F-12 (Gibco)] with the addition of 2 µg/ml laminin (Wako)^13,14^. We used cultured NSCs passaged about 10 times and frozen several times after dissociation from the ventral telencephalon of ICR mouse embryos at E14.5 (ref. ^13^). This NSC line expressing Nestin as well as Sox2 was clearly induced to enter quiescence after BMP exposure and induced back to active state after BMP withdrawal, as in the NS5 cell line (a kind gift from A. Smith^49^; Supplementary Fig. 1a, b). Adult NSCs were derived from the SVZ and DG of 7-week-old C57BL/6 mice. The DG and SVZ were surgically isolated from the three mice, dissociated by enzymes, purified in 20% Percoll by centrifugation, and cultured as adherent primary cell cultures in proliferation medium [20 ng/ml EGF, 20 ng/ml bFGF, P/S, B27 supplement (Gibco), 1× Glutamax (Gibco), 2 mg/ml heparin in Neurobasal medium (Gibco)]^50^. To induce quiescence, we plated 1 × 10^6^ cells in six-well dishes or 2 × 10^5^ cells (or 1 × 10^5^ cells only for Supplementary Fig. 2c) in 24-well dishes. The next day, we replaced the proliferation medium with quiescence medium [50 ng/ml BMP4 (R&D Systems) in medium of the same composition as proliferation medium except lacking EGF] after three washes with phosphate-buffered saline (PBS), and then cultured the NSCs in quiescence medium for 3 days^14^.

### Preparation of *Tfeb*-KO NSCs

*Tfeb*(flox/flox) and *Tfeb*(flox/+);Ai14 mice were crossed, and embryos were harvested at E14.5. After dissociation from the ventral telencephalon, cells were seeded on poly-l-ornithine (Sigma)– and fibronectin (R&D Systems)-coated dishes, and then cultured in proliferation medium as described in the Cell Culture section. To delete the floxed sequences in vitro, pCAG-Cre was transiently transfected by lipofection using ViaFect (Promega), and tdTomato-positive cells were sorted using a FACS AriaII SORP (BD).

### Proliferation assays and inhibitor treatments to NSCs

To monitor NSC proliferation, we analyzed NSCs by immunostaining of mouse anti–Ki-67 antibody (BD Pharmingen) and Click-IT EdU detection (Invitrogen) after 4 h exposure to EdU, and co-stained with rabbit anti-Sox2 antibody (EMD Millipore) or DAPI (Sigma) to count all cells. Cell images were obtained on AF6000 (Leica) and BZ-X (Keyence). Cell counting was performed using the ImageJ software for images captured on the AF6000, and Hybrid cell count (Keyence), an algorithm for cell counting, for images captured on the BZ-X. Six to eight images were collected under each condition. Inhibitors used for cell culture were obtained from the indicated suppliers: bafilomycin A1 (Sigma), SAR405 (Cayman Chemical), Dynasore (Adipogen), thapsigargin (Adipogen), nocodazole (Adipogen), DAPT (Calbiochem), AG1478 (Cell Signaling Technology), Torin-1 (Cayman Chemical), and rapamycin (Wako). To measure protein stability, cells were incubated with 10 µg/ml cycloheximide (Sigma) to block protein synthesis.

### Measurement of proteasomal and lysosomal activity

For measurement of 26S proteasome activities ^9,15^, cell lysate was extracted in proteasome activity assay buffer (50 mM Tris-HCl [pH 7.5], 250 mM sucrose, 5 mM MgCl_2_, 0.5 mM EDTA, 2 mM ATP, 1 mM DTT). Six micrograms of total protein extract was transferred to a 96-well plate, and substrate was added. The fluorogenic substrates Suc-Leu-Leu-Val-Tyr-MCA (Peptide Institute), Boc-Leu-Arg-Arg-MCA (Peptide Institute), and Ac-Nle-Pro-Nle-Asp-AMC (Enzo), all at 100 µM working concentration, were used to measure the chymotrypsin-like, trypsin-like, and caspase-like activities of the proteasome, respectively. Fluorescence was monitored every 5 min for 1 h at 37 °C on an ARVO X3 microplate reader (PerkinElmer) using a 355-nm excitation filter and a 460-nm emission filter. Protein concentration was determined using the DC protein assay (Bio-Rad). Inhibitors specific for the proteasome and cathepsins were epoxomicin (Peptide Institute) and cathepsin inhibitor I (Calbiochem), respectively, both used at a working concentration of 20 µM. Measurement of lysosomal cathepsin activity was performed by using a Magic Red cathepsin L assay kit (Immunochemistry). NSCs were incubated with a substrate to visualize lysosome activity in living cells for 30 min and then incubated with Hoechst 33342 for 5 min according to the manufacture’s instructions. Cells were fixed with 4% PFA and observed on an AF6000 microscope. Image intensities were analyzed using the ImageJ software.

### Luciferase reporter assay

The reporter plasmid encoding TFEB-promoter–driven luciferase was transfected into aNSCs with pCMV-Renilla to normalize transfection efficiency. Transfected NSCs were cultured in quiescent or proliferation medium for 3 days, and then analyzed using the PicaGene Dual Sea Pansy Luminescence Kit (Toyo Ink). The TFEB-promoter–driven luciferase reporter was a gift from Albert La Spada (Addgene plasmid # 66801).

### Plasmids and lentivirus for TFEB mutants

Mouse *Tfeb* (NM_001161722) was cloned from cDNA of mouse ES cells and subcloned into pCW57.1, a gift from David Root (Addgene plasmid #41393) after fusion of GFP to the C-terminal end of TFEB and site-directed mutagenesis to generate the constitutive-active mutants S141A and S210A. Ser141 and Ser210 were identified as phosphorylation sites in mouse TFEB based on sequence conservation with human TFEB. Lentiviral particles were produced by co-transfection with the packaging plasmids^51^ into Hek 293T cells, concentrated by centrifugation of the supernatants, and transducted into NSCs. Transducted NSCs were established after selection in 0.5 µg/ml puromycin, and then cultured in 3 µg/ml doxycycline to induce expression of TFEB-GFP.

### Lentivirus injection into the DG

For specific expression in NSCs in vivo, the mouse *Gfap* promoter^29^ or *Hes5* promoter^37^ was subcloned into vector CSII^51^. Lentiviral particles were produced in HEK 293T cells cultured in OptiPRO serum-free medium (Gibco) and concentrated by centrifugation after washing with PBS. Viruses were delivered stereotactically into the DG of 6-week-old C57BL/6 male mice using the following coordinates: 1.9 mm posterior to bregma, 1.4 mm lateral to the midline, and 1.75 mm below the dura.

### Western blot and qPCR

For western blotting, cells were lysed in lysis buffer (50 mM Tris-HCl [pH 8.0], 100 mM NaCl, 5 mM MgCl_2_, 0.5% Nonidet P-40, Complete™ protease inhibitor cocktail (Roche), 1 mM phenylmethylsulfonyl fluoride, 250 U/ml Benzonase (Sigma), 10 mM β-glycerophosphate, 1 mM sodium orthovanadate, 1 mM NaF, and 1 mM sodium pyrophosphate) on ice for 30 min, and then boiled after the addition of 1% SDS. Protein concentration of total cell lysate was measured by the DC protein assay, and 20 µg protein from each sample was loaded for SDS-PAGE. Western blots were visualized by chemiluminescence using Amersham ECL (GE) or ECL prime (GE), quantified on an LAS3000 image analyzer (Fujifilm), and normalized against the corresponding intensity of β-actin. The following primary antibodies were used for western blotting: rabbit anti-actin (Sigma), mouse anti-cyclin D1 (A-12; Santa Cruz Biotechnology), rabbit anti-EGFR (EMD Millipore), rabbit anti-phospho-EGF receptor (Tyr1068; Cell Signaling Technology), rabbit anti-Hes1 (ref. ^48^), rabbit anti-LC3 (Wako), rabbit anti-phospho-MEK1/2 (Ser221; Cell Signaling Technology), rabbit anti-cleaved Notch1 (Val1744; Cell Signaling Technology; to detect only activated Notch [NICD]), mouse anti-Notch1 (C-term, Novus; to detect all Notch1 protein, including full-length, NTMD, NEXT, and NICD), rabbit anti-Sox2, and rabbit anti-TFEB (Bethyl Laboratories). Secondary antibodies were HRP-conjugated anti-mouse and anti-rabbit antibodies (GE Healthcare). Western blots accompanied with molecular weight markers were provided as Source Data Files. For qPCR, total RNA was extracted using the RNeasy Plus mini kit (Qiagen) and analyzed on a 7500 Real-Time PCR system (Applied Biosystems). RNA levels were normalized against the corresponding levels of β-actin mRNA. Primers for real-time PCR were purchased from TaKaRa.

### Immunostaining and LysoTracker staining

NSCs were fixed with 4% PFA in PBS, permeabilized with 0.1% Triton X-100 in PBS, blocked in 5% normal goat serum (NGS)/0.1% Triton X-100 in PBS, and stained with the following antibodies: rat anti-Lamp1 (1D4B; Developmental Studies Hybridoma Bank), rabbit anti-EGFR, mouse anti–Ki-67, rabbit anti-Sox2, mouse anti-Nestin (BD Pharmingen), rabbit anti-GFAP (Sigma), and rabbit anti-TFEB (Bethyl) (primary) and Alexa Fluor-conjugated goat species-specific anti-IgG (Thermo Fisher Scientific) (secondary) in 1% NGS/0.1% Triton X-100 in PBS. Images were obtained on an LSM 510 (ZEISS). For processing of brain tissues, mice were transcardially perfused with 4% PFA in PBS. Brains were postfixed with 4% PFA in PBS, cryoprotected by sequential incubation with 10%, 20%, and 30% sucrose in PBS, and embedded and frozen in OCT (Tissue TEK). To detect lysosomes, cryosections 20 µm thick were treated with 10 mM sodium citrate buffer (pH 6.0) at 80 °C for 5 min for antigen retrieval, cooled at room temperature, blocked with 5% normal donkey serum (NDS) or NGS/0.1% Triton X-100 in PBS, and stained with the following antibodies: mouse anti–Ki-67, chicken anti-GFP (Abcam), rat anti-GFP (Nacalai Tesque), rabbit anti-DsRed (Clontech), goat anti-cathepsin B (R&D systems), goat anti-cathepsin L (R&D systems), or rat anti-Lamp1 (primary antibodies), followed by Alexa Fluor-conjugated donkey or goat species-specific anti-IgG (secondary antibodies), in 1% NDS or NGS containing 0.1% Triton in PBS. Secondary antibodies were conjugated with Alexa Fluor 405 for Ki-67, Alexa Fluor 488 for GFP, Alexa Fluor 594 for mCherry, and Alexa Fluor 647 for cathepsins and Lamp1. In Fig. 4a and Supplementary Fig. 4, we set the colors of Alexa Fluor 647 to red and Alexa Fluor 594 to blue to clearly depict immunostaining with the Alexa Fluor 647-conjugated antibody. Confocal z-stack images were obtained on an LSM880 (ZEISS). Figures show projection images. To count NSCs, DCX-positive cells, and cleaved-caspase-3–positive cells in *Tfeb* cKO mice, every sixth slice of 50-µm cryosections taken along the caudal–rostral axis throughout the DG was immunostained by a free floating method. The slices (six per mouse) were blocked with 5% normal donkey serum (NDS) or NGS/0.3% Triton X-100 in PBS, and then stained with the following antibodies: rabbit anti–Ki-67, mouse anti-GFAP (Sigma), goat anti-Sox2 (R&D Systems), rabbit anti-Sox2, goat anti-DCX (Santa Cruz Biotechnology), or rabbit anti–cleaved-caspase-3 (Cell Signaling Technology) (primary antibodies), followed by Alexa Fluor-conjugated donkey or goat species-specific anti-IgG (secondary antibodies), in 1% NDS or NGS containing 0.3% Triton in PBS. Secondary antibodies were conjugated with Alexa Fluor 405 for GFAP, Alexa Fluor 488 for Ki-67, DCX or cleaved-caspase-3, and Alexa Fluor 647 for Sox2. tdTomato was not stained with an antibody. For BrdU detection, every sixth slice (total six slices) or every tenth slice (total four slices) of a 50-µm section was incubated in 2 N HCl for 30 min, neutralized with 0.1 M sodium tetraborate, and then immunostained with rabbit anti-GFAP, rat anti-BrdU (Serotec), mouse anti-RFP (Abcam), and goat anti-Sox2 antibodies. Confocal z-stack images through 50-µm sections were obtained on an LSM880 Airy (ZEISS) and analyzed using the Imaris software (Bitplane). Specificity of TFEB antibody in IHC was confirmed by immunostaining using liver sections (Supplementary Fig. 10). For LysoTracker staining to detect lysosomes in vitro, quiescent NSCs were incubated in medium containing 50 nM LysoTracker Green DND-26 (Invitrogen) for 30 min, and then observed on an AF6000 microscope after a medium change.

### Three-dimensional image analyses using the Imaris software

To quantify lysosomes in NSC in GFAP-GFP;Nestin-mCherry-NLS double-transgenic mice, 3D images from z-stack images obtained by the confocal microscopy (LSM780 and LSM880) with a ×40 objective were reconstructed in Imaris, and a surface was first created using the GFP channel to extract GFP-positive cells. Subsequently, this surface was manually separated in single cells, and the surfaces were used to quantify cathepsin L signals in each cell type (eight images, 32 cells). To count NSCs, aNSCs, and DCX-positive cells in conditional knockout mice and controls, we used the Imaris software to reconstruct 3D images from z-stack images of the entire medial-lateral length of the DG in a hemisphere, using images obtained by confocal microscopy (LSM880 Airy) with a ×10 objective. To count NSCs in the DG, we made a colocalization channel for the tdTomato and Sox2 signals, created spots, as shown in Fig. 7f, l, for counting from colocalization signals 3 µm in diameter, excluded the spots in the outside of the SGZ as well as the spots of astrocytes identified from GFAP staining in the 3D images, and then confirmed the remains of spots with GFAP expression. To count aNSCs, we made another colocalization channel for the Ki-67, tdTomato, and Sox2 signals, created spots from triple-positive signals 3 µm in diameter, and then counted spots only in NSCs as described above for NSC counting. The same procedure was performed to count BrdU-positive cells. To count DCX-positive cells, we made a colocalization channel for DCX and tdTomato, and created spots from double-positive signals 4 µm in diameter. The number of cleaved-caspase-3–positive cells among tdTomato-positive cells in the SGZ was determined by manual counting. All quantifications for cell counting were performed through the entire rostrocaudal length of the DG.

### Slice culture, immunostaining, and cell counting

Slice preparation was performed by standard procedures. In brief, brains from 6-month-old GFAP-GFP;Nestin-NLS-mCherry double-transgenic mice were dropped into cutting solution (280 mM sucrose, 2 mM KCl, 10 mM HEPES-NaOH [pH 7.4], 0.5 mM CaCl_2_, 10 mM MgCl_2_, and 10 mM glucose). After the brain was cut at the midline, regions including the whole DG were sliced into six 300-µm-thick slices on a vibratome (Leica, VT1200S), yielding 12 slices of DG from each brain. Slices were incubated in bath solution (135 mM NaCl, 50 mM KCl, 10 mM CaCl_2_, 10 mM MgCl_2_, and 100 mM HEPES-NaOH [pH 7.4]) with bubbling oxygen and transferred on Millicell Cell Culture Inserts (0.4-µm pore size, 30-mm diameter; EMD Millipore) in six-well culture plates containing culture medium (5% horse serum and 5% FBS in bath solution). Brain slices were covered with Cellmatrix Type I-A (Nitta Gelatin) and incubated for 15 h (37°C, 5% CO_2_, 80% O_2_). Six slices from each brain hemisphere were treated with DMSO (control) or 20 nM BafA, which was added to both the culture medium and gel. Slices were fixed with 4% PFA in PBS, and then the collagen gel was removed with forceps. Fixed slices were immunostained by permeabilizing with 0.3% Triton X-100 in PBS, blocking with 5% NGS/0.3% Triton X-100 in PBS, primary antibody incubation (chicken anti-GFP, mouse anti-RFP (MBL), and rabbit anti–Ki-67 (Thermo Fisher Scientific) in 2% NGS/0.3% Triton X-100 in PBS), washing with 5% bovine serum albumin in PBS, and secondary antibody incubation (Alexa Fluor 488-conjugated goat anti-chicken, Alexa Fluor 594-conjugated goat anti-mouse, and Alexa Fluor 647-conjugated goat anti-rabbit with DAPI in 2% NGS/0.3% Triton X-100 in PBS). Samples were fixed again for 5 min in 4% PFA in PBS, and then incubated in CUBIC1 solution (25% urea, 25% *NNN*′*N*′-tetrakis (2-hydroxypropyl) ethylenediamine [Quadrol], 15% Triton X-100) to make them transparent for imaging^52^. These experiments used ten 6-month-old dTg mice. For counting of triple-positive cells, confocal z-stack images through 300-µm-thick slices were obtained on an LSM780 (ZEISS) or LSM880. Manual counts were performed in a blinded fashion through the whole SGZ using the ZEN2.1 software (black edition, ZEISS). Charts in Fig. 4e, f show total numbers of aNSCs and NSCs per brain hemisphere.

### Statistical analysis

Results are presented as means ± s.e.m. Statistical analyses were performed using the KaleidaGraph software. Statistical differences were examined using Student’s *t*-test and one-way ANOVA: *P* values < 0.05 were considered significant. All experiments were performed in duplicate or triplicate.

### Reporting summary

Further information on research design is available in the Nature Research Reporting Summary linked to this article.

## Supplementary information

Supplementary information

Peer Review

Reporting Summary

Description of Additional Supplementary Files

Supplementary Data 1–2

Supplementary Movie 1

Supplementary Movie 2

## Data Availability

Microarray data that support the findings of this study have been deposited in Gene Expression Omnibus with the accession codes GSE130018. Our microarray results (Supplementary Data 1 and 2), movies (Supplementary Movies 1 and 2), and all western blots with molecular weight markers were provided as Source Data Files. All other relevant data are available from the corresponding author (T.K.) upon reasonable request.

## Source data

Source Data
