# Supplementary material for: Enhanced lysosomal degradation maintains the quiescent state of neural stem cells
Source: Nat Commun. 2019 Nov 29;10:5446. doi: 10.1038/s41467-019-13203-4 (PMC6884460; doi:10.1038/s41467-019-13203-4)
Supplement: Supplementary file 1 — Supplementary information [file 41467_2019_13203_MOESM1_ESM.pdf]

Supplementary Information

Enhanced lysosomal degradation maintains the quiescent state of neural stem cells

Kobayashi, et al.

Supplementary Figures

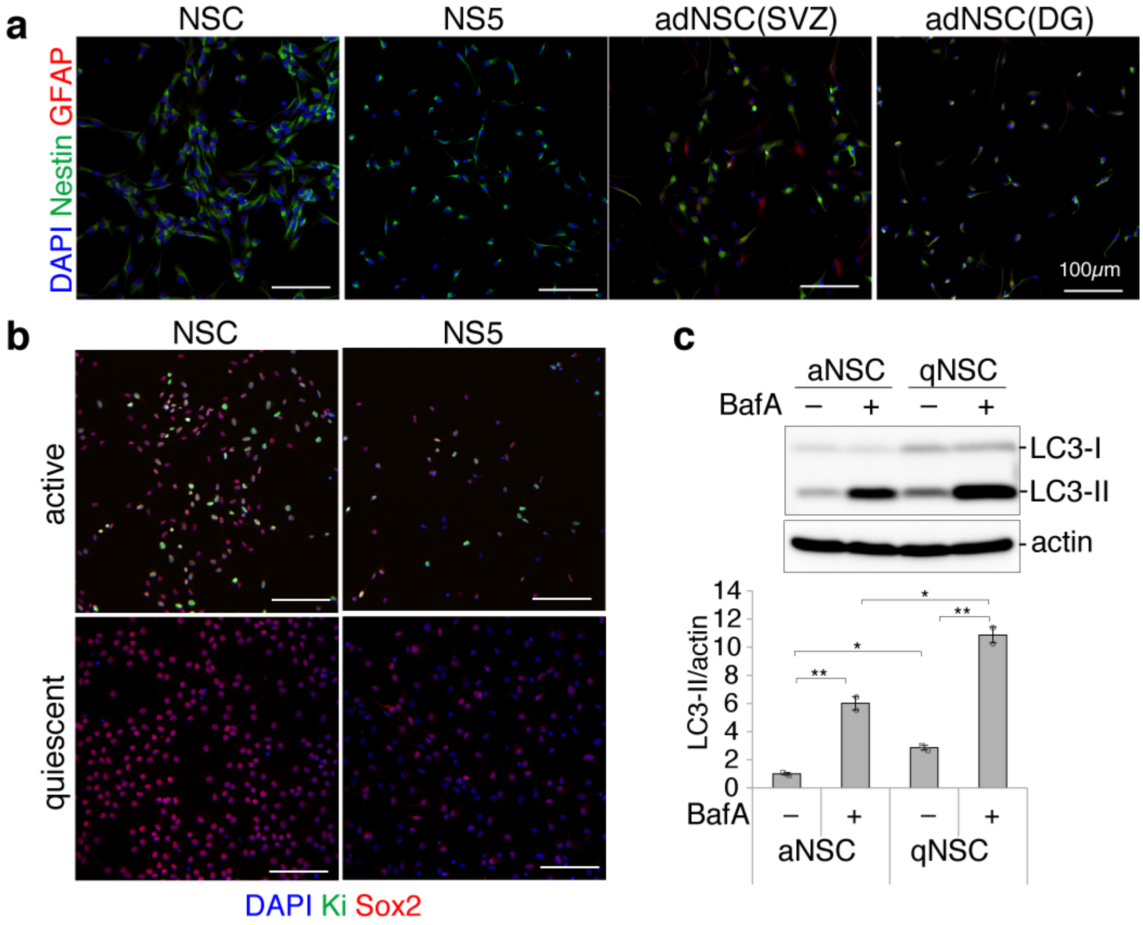

Supplementary Figure 1. Several NSC lines and their quiescence.

a. Comparison of neural stem cells used in this study. Immunocytochemistry of Nestin (green) and GFAP (red) with DAPI (blue) in NSCs (NSC), NS5 cells (NS5), and adult NSCs from the SVZ [adNSC(SVZ)] and DG [adNSC(DG)]. **b.** Immunocytochemistry of Ki-67 (green) and Sox2 (red) with DAPI in the active and quiescent states induced by BMP in NSCs and NS5 cells. **c.** LC3-II turnover assay. LC3-II levels were increased in qNSCs even in the presence of the lysosomal inhibitor, BafA. Cells were treated with BafA for 4 h. Lower panel shows the relative protein level of LC3-II, normalized against β-actin (n = 2). Data represent means ± s.e.m. (*$P < 0.05$, **$P < 0.01$; Student's *t* test). Scale bars, 100 μm. Source data of immunoblots are provided as a Source Data file.

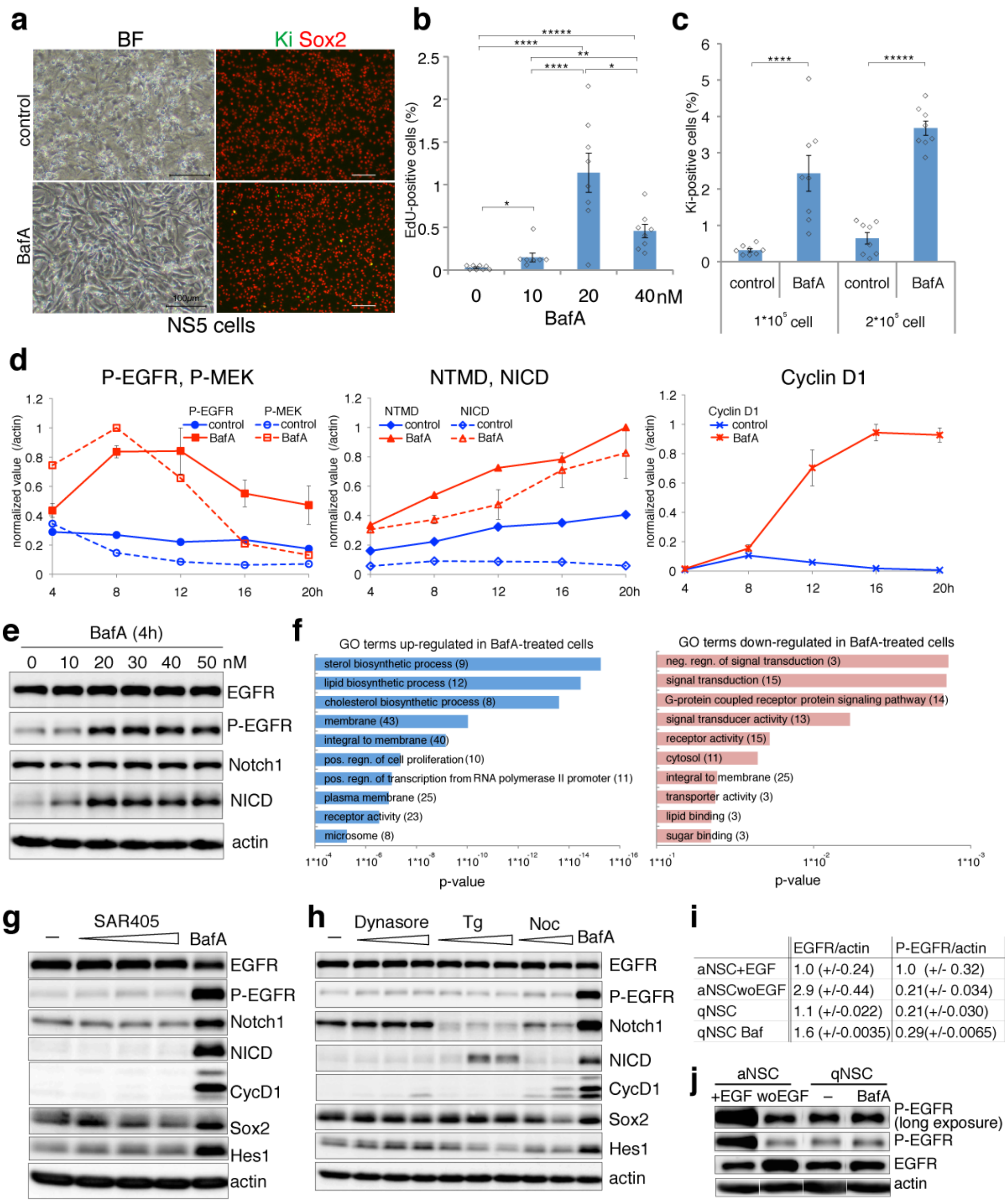

	EGFR/actin	P-EGFR/actin
aNSC+EGF	1.0 (+/-0.24)	1.0 (+/- 0.32)
aNSCwoEGF	2.9 (+/-0.44)	0.21(+/- 0.034)
qNSC	1.1 (+/-0.022)	0.21(+/-0.030)
qNSC Baf	1.6 (+/-0.0035)	0.29(+/-0.0065)

Supplementary Figure 2. Lysosomal inhibition in qNSCs.

a. BafA treatment in quiescent NS5 cells. BafA increased the abundance of Ki-67–positive NS5 cells in quiescence medium. Cells were stained for Sox2 (red) and Ki-67 (green). **b.** Percentage of EdU-positive cells in DAPI-stained cells after treatment of qNSCs with the indicated concentrations of BafA. **c.** Percentage of Ki-67–positive in Sox2-positive cells after BafA treatment of qNSCs at the indicated cell densities. **d.** Quantification of protein bands of P-EGFR, and P-MEK (left), NTMD, and NICD (middle), and Cyclin D1 (right) in BafA-treated (red lines) and control qNSCs (blue lines), based on data shown in Fig. 2d. **e.** Immunoblot of qNSCs

after treatment with various concentration of BafA. P-EGFR and NICD increased and plateaued in qNSCs after treatment with 20 nM BafA for 4 h. **f.** Gene Ontology analysis of BafA-treated qNSCs. GO terms upregulated (left) or downregulated (right) in qNSCs treated with BafA for 4 h. The x-axis represents the *P* value, which was calculated by hypergeometric distribution and corrected by multiple hypothesis testing. The number of genes included in each category is shown in brackets. BafA treatment of qNSCs affected transcription of genes related to lipid biosynthesis, cell proliferation, and signal transduction. **g.** Immunoblot of qNSCs after incubation with SAR405, an autophagy inhibitor. Cells were incubated for 24 h with 0.1, 1, or 10 μM of SAR405 or 20 nM BafA. **h.** Immunoblot of qNSCs after inhibition of endocytosis, ER calcium ATPase, or vesicular transport. Cells were treated with 40 nM, 400 nM, or 4 μM Dynasore, 10 nM, 100 nM, or 1 μM thapsigargin, or 2 μg/ml or 20 μg/ml nocodazole for 16 h. The BafA-treated sample was used as a control. **i.** P-EGFR and EGFR protein levels in each condition at time 0 of the experiment shown in Fig. 3e. **j.** Representative blots of protein bands in an identical membrane, used for the quantification (i). Scale bars, 100 μm. Data represent means ± s.e.m. (*$P < 0.05$, **$P < 0.01$, ****$P < 0.001$, *****$P < 0.0001$; Student's t test). Source data of immunoblots are provided as a Source Data file.

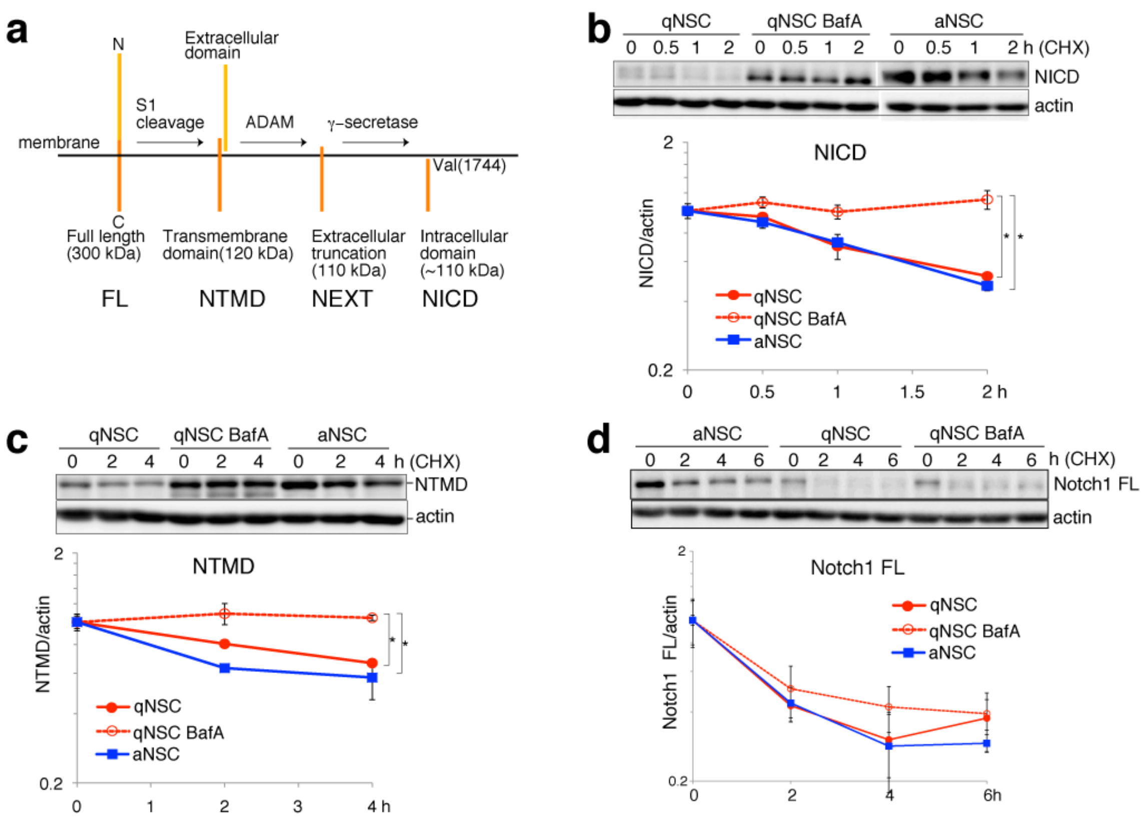

Supplementary Figure 3. Processing and stability of full-length Notch1 protein.

a. Processing pathway of Notch1 protein. Membrane integrated Notch1 protein (full-length) is sequentially cleaved by S1 protease, ADAM protease, and γ-secretase to yield the active form (NICD). **b–d.** Protein stability of NICD (b), NTMD (c), and full-length Notch1 (d) (n=2). For BafA-treated samples, BafA was added 4 h before all assays. Data represent means ± s.e.m. (**P* < 0.05, Student's *t* test). Source data of immunoblots are provided as a Source Data file.

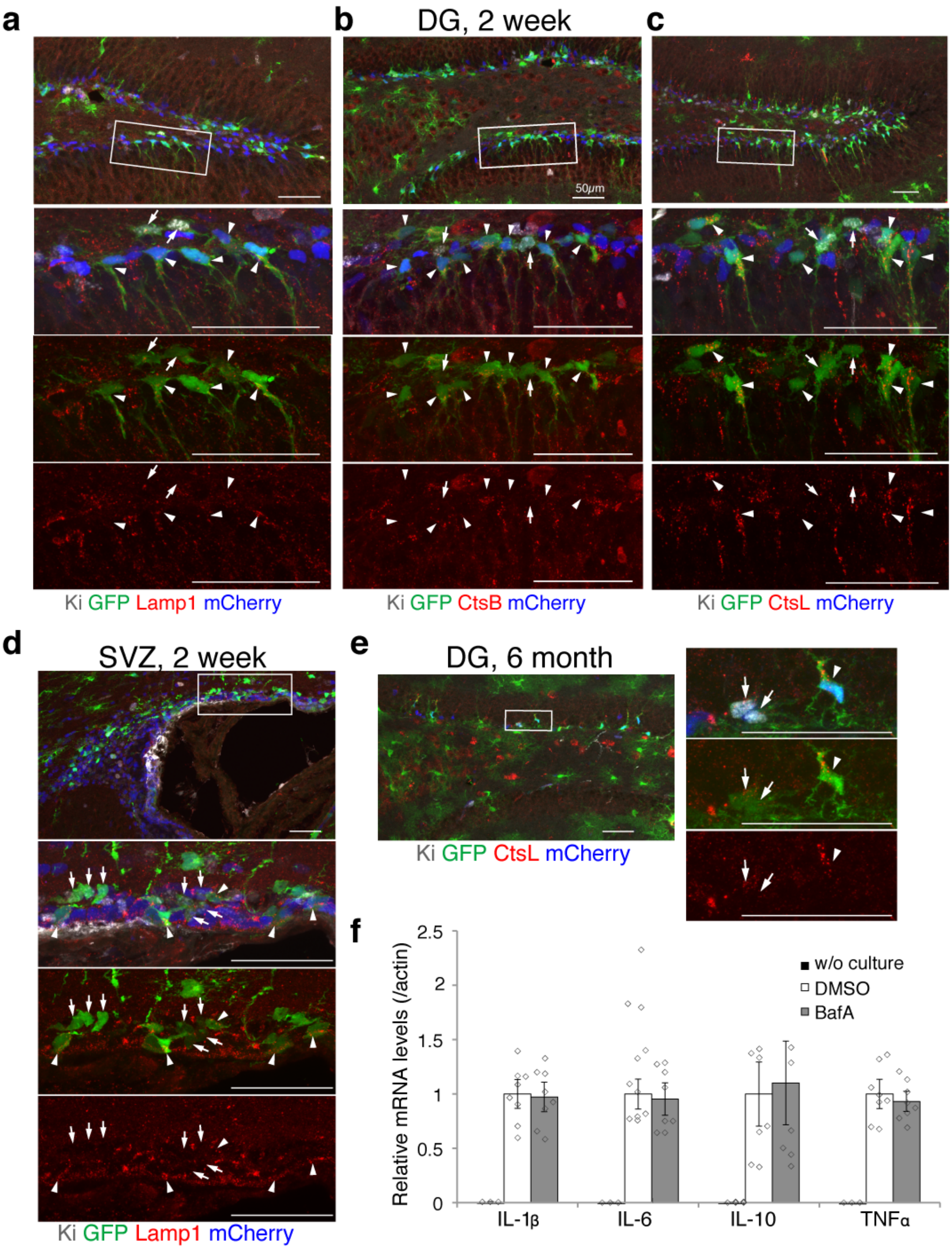

Supplementary Figure 4. Upregulation of lysosomal enzymes in qNSCs *in vivo* and acute injury response in cultured slices.

a-e. Immunohistochemistry of Lamp1, cathepsin B (CtsB), and cathepsin L (CtsL) in the DG (a-c, e) or the SVZ (d) in 2-week-old (a-d) or 6-month-old (e) GFAP-GFP;Nestin-NLS-mCherry mice. Proliferating NSCs expressed Ki-67 (gray), GFP (green), and mCherry (blue), but qNSCs were Ki-67–negative. Selected regions indicated by white squares in top panels are enlarged in lower panels. Proliferating NSCs (arrows) exhibited lower staining for cathepsin L and Lamp1 than qNSCs (arrowheads). In 6-month-old mice (c), Ki-67–positive cells were very rare. **f.** mRNA levels of genes involved in acute injury response in brain slices. Values are shown relative to those in control cultured slices (DMSO, white bars). Black bars correspond to slices before culture (left bars, w/o culture) and grey bars correspond to slices after culture in BafA medium (BafA). Scale bars, 50 μm. Data represent means ± s.e.m.

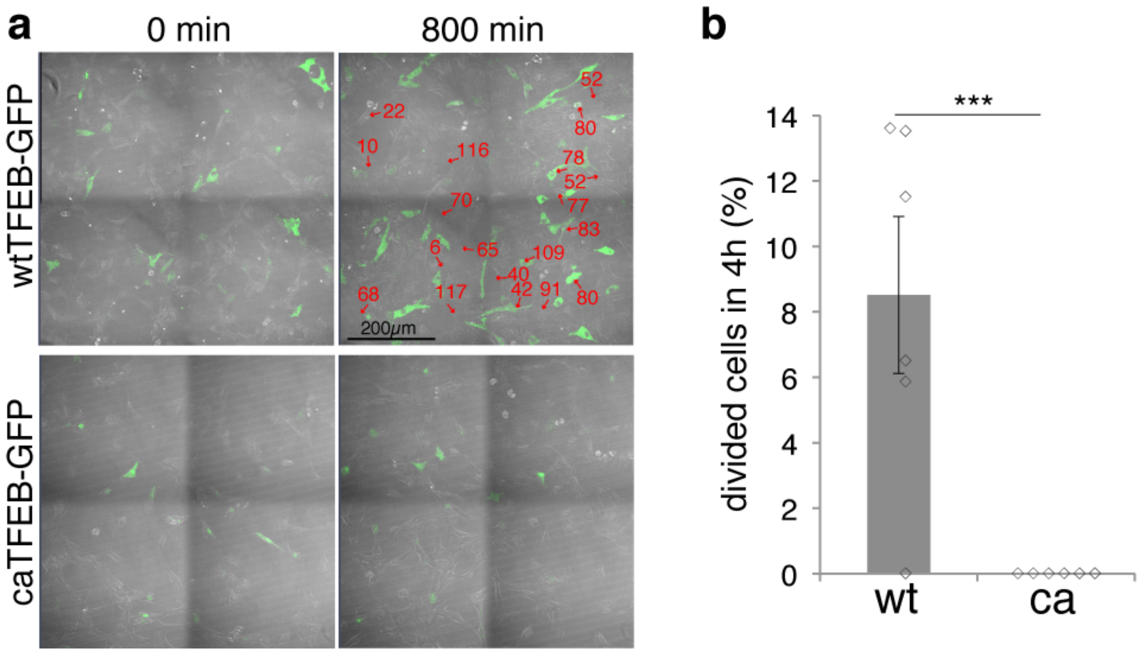

Supplementary Figure 5. Suppression of cell division in NSCs by caTFEB-GFP

a. Representative snapshots at 0 min and 800 min (frames #0 and #80) from real-time imaging of NSCs expressing wtTFEB-GFP (upper panels) and caTFEB (S210A)-GFP (lower panels) (Supplementary Movies 1, 2). Bright-field and fluorescence (GFP) images were acquired every 10 min for 24 h, starting 1 day after cells were seeded in medium containing doxycycline. Red arrows and numbers (red) indicate the positions of dividing cells and frame numbers, respectively. **b.** Percentage of cells that divided in every 4-h period. Data represent means ± s.e.m. (***P < 0.005; Student's t test, n=6).

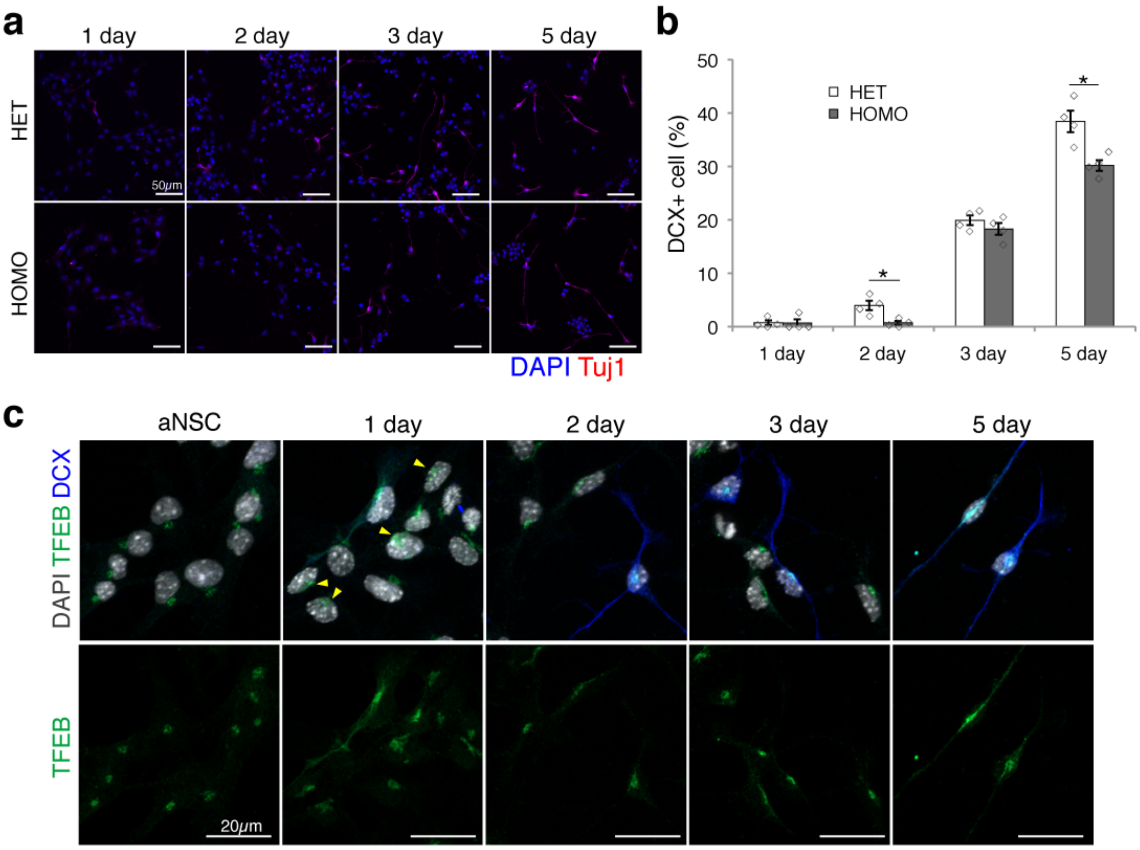

Supplementary Figure 6. Neuronal differentiation of TFEB-KO NSCs *in vitro*.

a. Immunostaining for the neuronal marker Tuj-1 (red) with DAPI after 1, 2, 3, and 5 days in differentiation medium to monitor neuronal differentiation from NSCs in *Tfeb*-KO and control cells. Scale bars, 50 μm. **b.** Percentage of DCX-positive cells among living *Tfeb*-KO (grey bars) and control cells (white bars). Data represent means ± s.e.m. (**P* < 0.05, Student's *t* test). **c.** Localization of TFEB during neuronal differentiation, detected by immunostaining for TFEB (green) and DCX (blue) and staining for DAPI (white). Yellow arrowheads indicate nuclear localization of TFEB on 1 day. Scale bars, 20 μm.

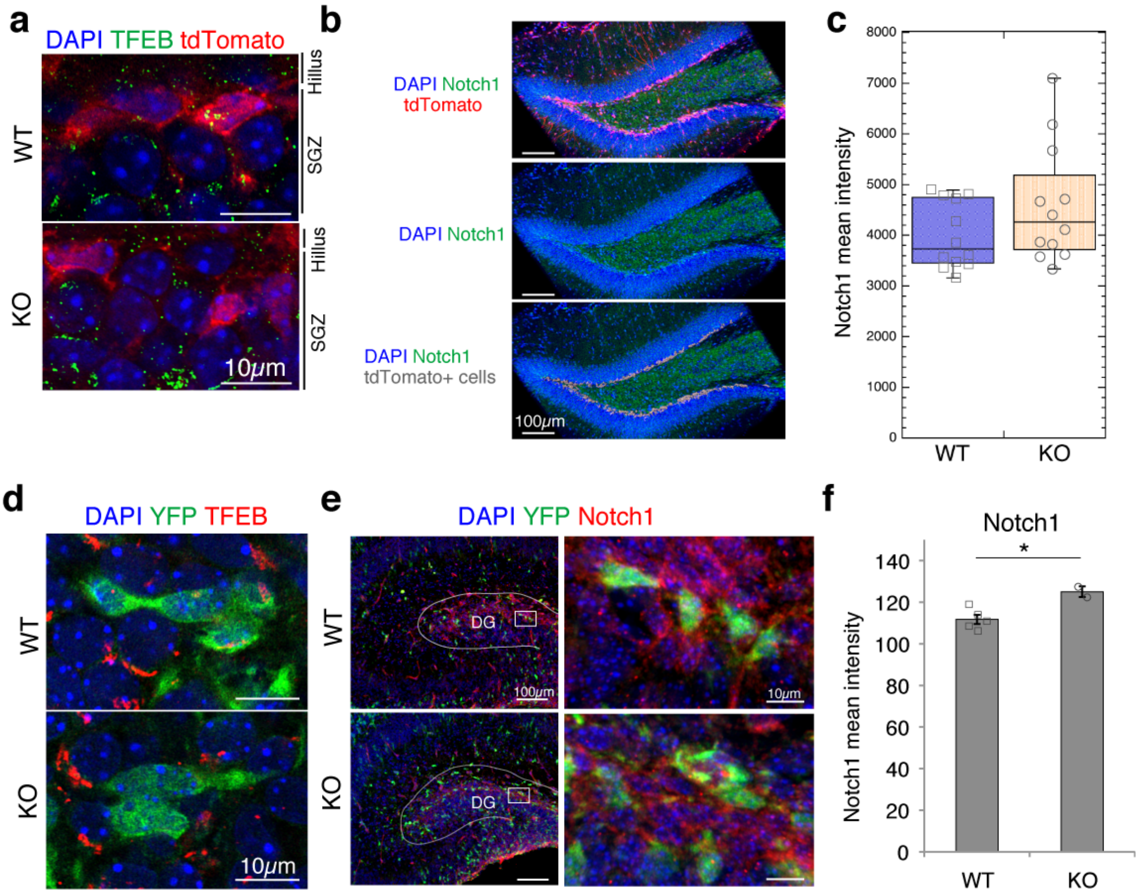

Supplementary Figure 7. Notch1 in *Tfeb*-cKO mice at adult and postnatal stage.

a. Immunostaining of TFEB (green) and tdTomato (red) with DAPI (blue) in the DG of *Tfeb*-cKO and wild-type mice on day 6 after tamoxifen administration at 8 weeks of age. Scale bars, 10 μm. **b.** Representative staining for Notch1 (green) and tdTomato (red) with DAPI (blue) in the DG of wild type. Lower panel is an image processed from the upper panel for the measurement of Notch1 intensity in Imaris software. Individual tdTomato-positive cells in the SGZ were visualized after creating surfaces (grey) in imaris. Scale bars, 100 μm. **c.** Notch1 intensity was quantified in individual cells, and the mean intensity in the DG was plotted in a box chart (four slices per mouse; n=3 [WT], n=3 [HOMO]; center line, median; box limits, upper and lower quartiles; whiskers, minimum and maximum). **d.** Immunostaining for TFEB (red) and YFP (green) with DAPI (blue) in the DG of *Tfeb*-cKO and wild-type mice at P0, obtained after tamoxifen administration of R26-stop-YFP;GLAST-Cre-ERT2 and *Tfeb*-flox;R26-stop-YFP;GLAST-Cre-ERT2 mice, respectively. Scale bars, 10 μm. **e.** Representative immunostaining of Notch1 at P0 in the hippocampus of wild-type and cKO mice. Premature DG, estimated from DAPI signal (ref. 1), is depicted by white lines. Regions indicated by white squares in the left panels are enlarged in the right panels. Scale bars, 10 (right panels) and 100 μm (left panels). **f.** Notch1 intensity in YFP-positive cells of the DG was quantified using Imaris software (n=5 for WT, n=3 for cKO). Data represent means ± s.e.m. (*$P < 0.05$; Student's t test).

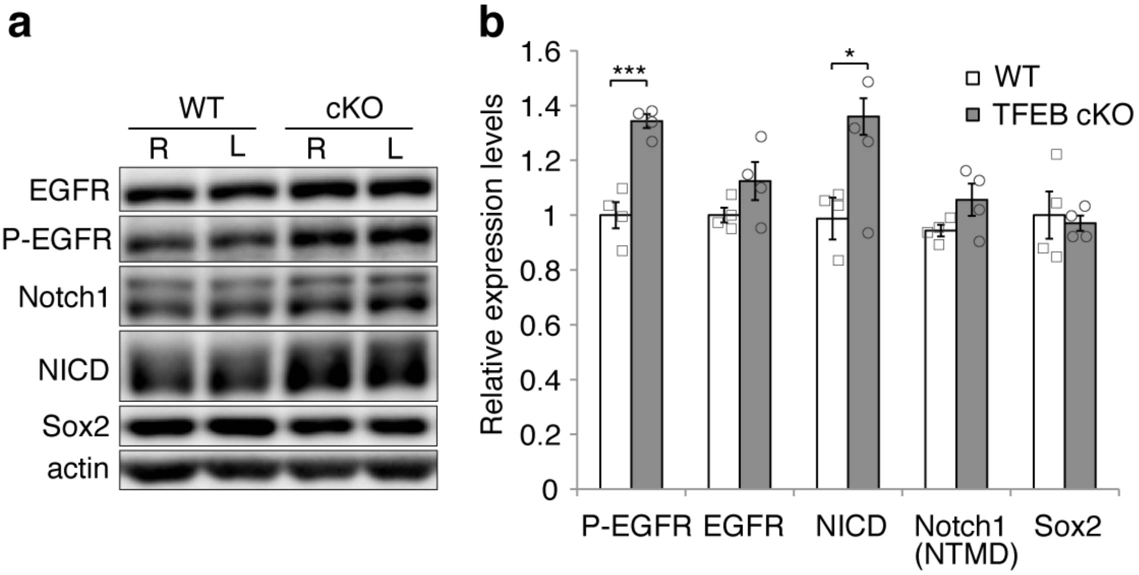

Supplementary Figure 8. Increase of P-EGFR and NICD in the DG of *Tfeb*-cKO mice.
a. Representative immunoblots of EGFR, Notch1, and their active forms in DG from right (R) and left brain (L) of *Tfeb*-cKO (cKO) and control mice (WT). Sox2 is a marker of NSCs. **b.** Relative quantification of P-EGFR, EGFR, NICD, Notch1, and Sox2 in DG of *Tfeb*-cKO (gray) and control mice (white) in (a) (n=4). Data represent means ± s.e.m. (*P < 0.05, ***P < 0.005; Student's t test). Source data of immunoblots are provided as a Source Data file.

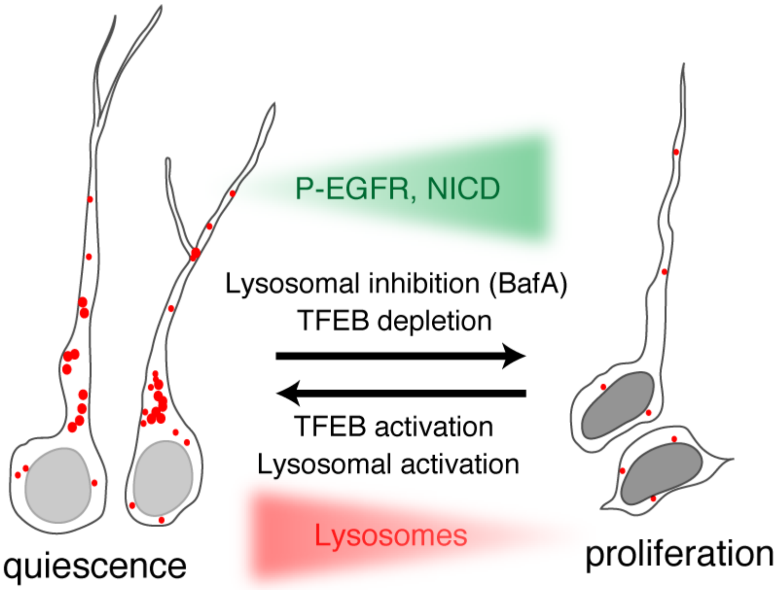

Supplementary Figure 9. Diagram summarizing of our findings.
Lysosomes are indicated by red dots.

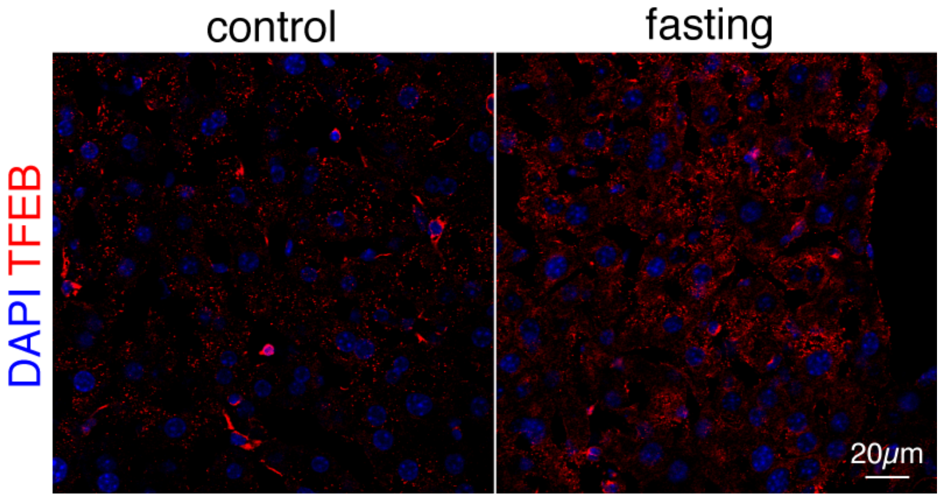

Supplementary Figure 10. Specificity of TFEB antibody.

TFEB is highly activated in the fasting liver (ref. 2). To confirm the specificity of TFEB antibody in IHC, livers from mice fasted for 1 day were examined by IHC. Twenty-micron cryosections were immunostained with rabbit anti-TFEB (Bethyl Laboratories, red) and DAPI (blue). Antigen retrieval was performed at 105°C for 5 min. TFEB IHC yielded stronger signals in fasting liver (right panel) than in control liver (left panel). This result demonstrates that the anti-TFEB antibody we used was specific.

Supplementary Methods

Microarray analysis of NSCs

NSCs were collected 4 h or 24 h after incubation in normal or BafA-containing quiescence medium (duplicate). For microarrays, total RNA was extracted using the RNeasy Plus mini kit (Qiagen), labeled with Cy5, and hybridized to oligo DNA microarrays (3D-Gene®, Toray Industries). For data comparison, the signal intensity was normalized by global normalization using the median from each microarray (Supplementary Data 1). Affected genes exhibiting 2-fold differential expression following BafA treatment in comparison with control samples were subjected to Gene Ontology (GO) analysis using GeneCodis 2.0 (ref. 3, http://genecodis.cnb.csic.es/help). The top ten GO terms from all biological processes, molecular functions, and cellular components are listed in Supplementary Figure 2f; all listed genes had $P < 0.05$, calculated by hypergeometric distribution, and false discovery rate (FDR) < 5%, corrected by multiple hypothesis testing.

qRT-PCR of brain slices

Brain slices were obtained from wild-type BL6/J mice at the age of 6 months, as described. Total RNA was prepared from brain slices just after slice preparation (for samples that were not subjected to cultures) or after culturing slices in medium containing DMSO or 20 nM BafA for 15 h. Four mice were used per condition. RNA was purified from brain slices dissolved in TRIzol (Invitrogen) and analyzed to quantify injury response factors: TNF α , IL-1 β , IL-6 and IL-10 using the primers reported previously (ref. 4).

Quantification of Notch1 receptor *in vivo*

Because Notch1 immunostaining gave much clearer signals in newborn mice than in adults, postnatal P0 mice as well as adult mice were used to quantify Notch1 protein *in vivo*. To obtain *Tfeb*-cKO P0 mice, *Tfeb*(f/+);GLAST-Cre-ERT2 were mated with *Tfeb*(f/+); R26R-stop-YFP(+/+) mice, and tamoxifen (5mg) was orally administrated to the resultant pregnant mice once a day for 2 days at E13.5 and E14.5. Pups were fixed at P0 with 4% PFA, and wild-type and cKO mice were analyzed after genotyping. Twenty-micron cryosections were immunostained with goat-anti Notch1 (Santa Cruz Biotechnology), rabbit anti-GFP (Invitrogen), and DAPI. Antigen retrieval was performed at 105℃ for 5 min. Cryosections from adult mice were stained by the same procedure using goat-anti Notch1 and mouse anti-RFP antibodies with

DAPI. Confocal z-stack images were obtained on an LSM 880 Airy and analyzed by Imaris. 3D object (named by surface) to capture individual YFP- or tdTomato-positive cells was created in Imaris software for their measurements. Notch1 intensity was measured in each cell in the premature DG of P0 mice, as well as in the SGZ of adult mice. Mean Notch1 intensity values from each section are displayed in plots.

Neural differentiation from NSCs

NSCs were differentiated into neurons *in vitro* as described on the web site of Thermo Fisher Scientific. NSCs were plated on a chamber slide ($2.85x10^4$ cells/cm^2) and cultured for 2 days, and then NSC medium was replaced with neural differentiation medium [Neurobasal medium (Gibco), 2% B-27 supplement, 2 mM GlutaMAX-I]. Half of the medium was replaced every day, and cells were cultured for 7 days. Cells were fixed, immunostained with rabbit ant–Tuj-1 (Abcam), goat anti-Dcx, and rabbit anti-TFEB antibodies and stained with DAPI. Images were obtained on LSM510 confocal microscope. Dcx-positive cells were processed using the Image J software, and were manually counted in living cells by DAPI staining.

Live-cell imaging of NSCs

One day before imaging, cells were plated in glass-based dishes in proliferation medium containing doxycycline. A dish was placed on an LSM880 to acquire bright field and GFP fluorescence images every 10 min for 24 hours using a 40x objective lens with 2x2 tiles and Z-stack. Images were represented by a maximum intensity projection. Arrows and numbers were drawn to indicate dividing cells and the frame in which cell division occurred. The Zen software (Zeiss) was used for processing.

Western blotting of the DG of *Tfeb*-cKO mice

DGs from *Tfeb*-cKO mice [Nestin-Cre-ERT2;*Tfeb*(flox/flox)] (ref. 5) and the control mice [*Tfeb*(flox/flox)] were surgically isolated, minced and lysed in lysis buffer (see Material and Methods) after homogenization with a homogenizer pestle (Greiner). Tamoxifen (0.5 mg) was administered to *Tfeb*-cKO mice at P14, P16 and P18.

Supplementary Notes

For mathematical simulation, we describe the dynamical process by

$$\frac{d}{dt}R = v - kRX - \mu_R R, \quad (1)$$

$$\frac{d}{dt}R_X = kRX - pR_X, \quad (2)$$

$$\frac{d}{dt}R_X^* = pR_X - \mu_p R_X^*, \quad (3)$$

$$\frac{d}{dt}R^* = - \mu_p R^*. \quad (4)$$

Here, t is time [h], and the protein concentrations are denoted by $X = [\mathrm{EGF}]$, $R = [\mathrm{EGFR}]$, $R_X = [\mathrm{EGFR\text{-}EGF}]$, $R^* = [\mathrm{P\text{-}EGFR}]$, and $R_X^* = [\mathrm{P\text{-}EGFR\text{-}EGF}]$. The parameters are given as follows: v is the translation rate for EGFR protein, k is the rate constant for the formation of EGFR-EGF complex, p is the phosphorylation rate, and μ_R and μ_p are the degradation rates for EGFR and P-EGFR, respectively. For simplicity, we treat X as a constant. We assume that at $t = 0$, which is the moment of medium change, the ratio β of EGFR is phosphorylated, i.e., $R(+0) = (1-\beta)R(-0)$ and $R^*(+0) = \beta R(-0)$. We also assume that v vanishes at $t = 0$. For qNSC with BafA, we further assume that μ_R vanishes at $t = 0$. For $t \to \infty$, our system approaches the equilibrium state given by

$$R = \frac{v}{kX + \mu_R}, \quad (5)$$

$$R_x = \frac{v}{p}\frac{kX}{kX + \mu_R}, \quad (6)$$

$$R_X^* = \frac{v}{\mu_p}\frac{k\,X}{kX + \mu_R}, \quad (7)$$

$$R^* = 0. \quad (8)$$

We are interested in the transient process of the amounts of the total EGFR and the phosphorylated EGFR proteins, respectively denoted by

$$R_{\mathrm{tot}} = R + R_X + R^* + R_X^*, \quad (9)$$

$$R_{\mathrm{tot}}^* = R^* + R_X^*. \quad (10)$$

We tuned the parameter values so as to fit the experimental data shown in Fig. 3e and Supplementary Fig. 2i, j, which describe the time-courses of R_{tot}^* and R_{tot} for $t > 0$ and the relative amount of R_{tot}^* and R_{tot} for the four different conditions at $t = +0$ (just after the moment of medium change), respectively. Our simulation results are shown below. As seen in

these results, the characteristics of the decay of P-EGFR proteins are well accounted by the difference of μ_p values between qNSC and aNSC conditions.

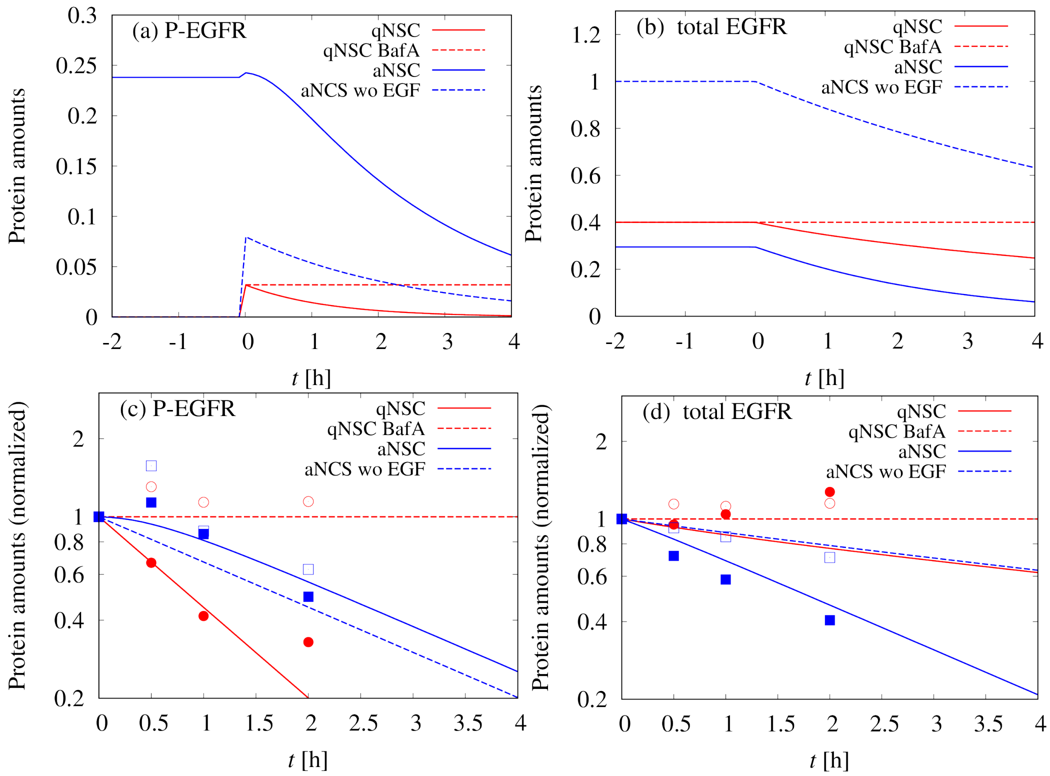

Simulation results show amounts of P-EGFR (a), total EGFR proteins (b), normalized P-EGFR (c), and normalized total EGFR proteins (d). The normalization was performed by the amounts of protein at $t = +0$. The lines and symbols show the simulation and experimental results, respectively. The filled and open symbols correspond to the solid and dashed lines. The experimental data is the same as those shown in Fig. 3e. We set $\mu_p = 0.8$ (qNSC), $\mu_p = 0.4$ (aNSC), $\mu_R = 0.1$, $v = 0.04$ (qNSC), $v = 0.1$ (aNSC), $kX = 5$, $p = 10$, $\beta = 0.08$.

Supplementary References

1. Carmona-Saez, P., Chagoyen, M., Tirado, F., Carazo, J. M. & Pascual-Montano., A. GENECODIS: A web-based tool for finding significant concurrent annotations in gene lists. *Genome Biology* **8**, R3 (2007).
2. Wang, L. et al. Increased inflammation and brain injury after transient focal cerebral ischemia in activating transcription factor 3 knockout mice. *Neuroscience* **220,** 100–108. (2012).
3. Settembre, C. et al. TFEB controls cellular lipid metabolism through a starvation-induced autoregulatory loop. *Nat Cell Biol* **15,** 647-658. (2013).
4. Imayoshi, I. et al. Temporal regulation of Cre recombinase activity in neural stem cells. *Genesis* **44,** 233-238. (2006).
