# Supplementary material for: Enhanced lysosomal degradation maintains the quiescent state of neural stem cells
Source: Nat Commun. 2019 Nov 29;10:5446. doi: 10.1038/s41467-019-13203-4 (PMC6884460; doi:10.1038/s41467-019-13203-4)
Supplement: Supplementary file 3 — Reporting Summary [file 41467_2019_13203_MOESM3_ESM.pdf]

nature research

Corresponding author(s): Taeko Kobayashi and Ryoichiro Kageyama

Last updated by author(s): Oct 12, 2019

Reporting Summary

Nature Research wishes to improve the reproducibility of the work that we publish. This form provides structure for consistency and transparency in reporting. For further information on Nature Research policies, seeAuthors & Referees and theEditorial Policy Checklist .

Statistics

For all statistical analyses, confirm that the following items are present in the figure legend, table legend, main text, or Methods section.

n/a	Confirmed	
☐	☒	The exact sample size (*n*) for each experimental group/condition, given as a discrete number and unit of measurement
☐	☒	A statement on whether measurements were taken from distinct samples or whether the same sample was measured repeatedly
☐	☒	The statistical test(s) used AND whether they are one- or two-sided<br>*Only common tests should be described solely by name; describe more complex techniques in the Methods section.*
☒	☐	A description of all covariates tested
☒	☐	A description of any assumptions or corrections, such as tests of normality and adjustment for multiple comparisons
☐	☒	A full description of the statistical parameters including central tendency (e.g. means) or other basic estimates (e.g. regression coefficient) AND variation (e.g. standard deviation) or associated estimates of uncertainty (e.g. confidence intervals)
☐	☒	For null hypothesis testing, the test statistic (e.g. *F*, *t*, *r*) with confidence intervals, effect sizes, degrees of freedom and *P* value noted<br>*Give P values as exact values whenever suitable.*
☒	☐	For Bayesian analysis, information on the choice of priors and Markov chain Monte Carlo settings
☒	☐	For hierarchical and complex designs, identification of the appropriate level for tests and full reporting of outcomes
☒	☐	Estimates of effect sizes (e.g. Cohen's *d*, Pearson's *r*), indicating how they were calculated

Our web collection on statistics for biologists contains articles on many of the points above.

Software and code

Policy information about availability of computer code

Data collection	Fluorescent images were acquired with the AIM (Zeiss), the Zen (Zeiss), the Leica Application Suite (LAS) and the BZ-X viewer (Keyence). Western blot images were acquired with the Image reader LAS3000 (Fujifilm). Quantitative PCR data was acquired with the SDS System Software (Applied Biosystems). Both luminescence for luciferase assay and fluorescence for protease activity assay in 96 well plate were acquired with the PerkinElmer 2030 Workistation (PerkinElmer).
Data analysis	Fluorescent images were processed and analyzed using the Zen, the BZ-X analyzer (Keyence), the Image J and the Imaris (Bitplane). Western blots were analyzed with the Image Gauge (Fujifilm) and the Image J. KaleidaGraph software (Synergy Software, Hulinks) was used for statistical analyses.

For manuscripts utilizing custom algorithms or software that are central to the research but not yet described in published literature, software must be made available to editors/reviewers. We strongly encourage code deposition in a community repository (e.g. GitHub). See the Nature Research guidelines for submitting code & software for further information.

Data

Policy information about availability of data

All manuscripts must include a data availability statement. This statement should provide the following information, where applicable:

- Accession codes, unique identifiers, or web links for publicly available datasets
- A list of figures that have associated raw data
- A description of any restrictions on data availability

Microarray data that support the findings of this study has been deposited in GEO with the GSE130018 accession code. The data that support the finding of this study are available from the corresponding author (TK) upon reasonable request.

Field-specific reporting

Please select the one below that is the best fit for your research. If you are not sure, read the appropriate sections before making your selection.

☒ Life sciences ☐ Behavioural & social sciences ☐ Ecological, evolutionary & environmental sciences

For a reference copy of the document with all sections, see nature.com/documents/nr-reporting-summary-flat.pdf

Life sciences study design

All studies must disclose on these points even when the disclosure is negative.

Sample size	Sample size for each experiment is indicated in the figure legend for each experiment.
Data exclusions	No data were excluded from experiments.
Replication	All experimental findings were reliably reproducible.
Randomization	For in vitro studies, several types of neural stem cell lines were used. For in vivo studies, animals were chosen by genotypes. Sex-specific differences were eliminated by including almost equal numbers of both sexes.
Blinding	Analyses were performed in a blinded fashion after giving randomized sample number to each sample.

Reporting for specific materials, systems and methods

We require information from authors about some types of materials, experimental systems and methods used in many studies. Here, indicate whether each material, system or method listed is relevant to your study. If you are not sure if a list item applies to your research, read the appropriate section before selecting a response.

Materials & experimental systems

n/a	Involved in the study	
☐	☒	Antibodies
☐	☒	Eukaryotic cell lines
☒	☐	Palaeontology
☐	☒	Animals and other organisms
☒	☐	Human research participants
☒	☐	Clinical data

Methods

n/a	Involved in the study	
☒	☐	ChIP-seq
☒	☐	Flow cytometry
☒	☐	MRI-based neuroimaging

Antibodies

Antibodies used	We used mouse anti–Ki-67 antibody (BD Pharmingen), rabbit anti-Sox2 antibody (EMD Millipore), rabbit anti-actin antibody (Sigma), mouse anti–cyclin D1 antibody (A-12, Santa Cruz Biotechnology), rabbit anti-EGFR antibody (EMD Millipore), rabbit anti–phospho-EGF receptor antibody (Tyr1068, Cell Signaling Technology), rabbit anti-Hes1 antibody (ref. 48), rabbit anti-LC3 antibody (Wako), rabbit anti–phospho-MEK1/2 antibody (Ser221, Cell Signaling Technology), rabbit anti–cleaved Notch1 antibody (Val1744, Cell Signaling Technology), mouse anti-Notch1 antibody (C-term, Novus), rabbit anti-TFEB antibody (Bethyl Laboratories), HRP-conjugated anti-mouse and anti-rabbit antibodies (GE Healthcare), rat anti-Lamp1 antibody (1D4B, Developmental Studies Hybridoma Bank), mouse anti-Nestin antibody (BD Pharmingen), rabbit anti-GFAP antibody (Sigma), mouse anti-GFAP antibody (Sigma), Alexa Fluor–conjugated goat species-specific anti-IgG antibody (Thermo Fisher Scientific),chicken anti-GFP antibody (Abcam), rat anti-GFP antibody (Nacalai Tesque), rabbit anti-DsRed antibody (Clontech), goat ant–cathepsin B antibody (R&D systems), goat anti–cathepsin L antibody (R&D systems), goat anti-Sox2 antibody (R&D Systems), goat anti-DCX antibody (Santa Cruz Biotechnology), rabbit anti–cleaved-caspase3 (Cell Signaling Technology), rat anti-BrdU antibody (Serotec) and mouse anti-RFP antibody (abcam).
Validation	Specificity of commercially available antibodies was validated by data sheet provided by the manufacture. Hes1 antibody was validated in our previous report (ref.48). TFEB IHC re-validation was shown in supplementary figure 10.

Eukaryotic cell lines

Policy information about cell lines

Cell line source(s)	C3H10T1/2 mouse fibroblast cell line was obtained from ATCC.<br>NS5 cell line was obtained from A.Smith (ref. 49).<br>NSC line was obtained through passages about 10 times and freezes several times after dissociation from the ventral telencephalon of ICR mouse embryos at E14.5.

	Adult NSCs were derived from the SVZ and DG of 7-week-old C57BL/6 mice.
Authentication	Authentication of neural stem cell lines was indicated in manuscript.
Mycoplasma contamination	All cell lines were tested negative for mycoplasma contamination.
Commonly misidentified lines (See ICLAC register)	No commonly misidentified cell lines were used in this study.

Animals and other organisms

Policy information about studies involving animals; ARRIVE guidelines recommended for reporting animal research

Laboratory animals	We used Tfeb-flox mice (ref. 34), Ai14 reporter mice (Rosa-CAG-LSL-tdTomato-WPRE, stock 007908, The Jackson Laboratory), GLAST-CreERT2 mice (a kind gift from M. Götz, ref. 38, 39), GFAP-GFP;Nestin-NLS-mCherry double-transgenic mice, Nestin-CreERT2 mice (ref. 47). All mice were of the C57BL/6 background. Mice were maintained in our animal facility and housed in a 12:12 hour light–dark cycle.
Wild animals	No wild animals were used in this study.
Field-collected samples	This study did not involve samples collected from the field.
Ethics oversight	Animal care and experiments were conducted in accordance with the guidelines of the animal experiment committee of Kyoto University. We have complied with all relevant ethical regulations for animal testing and research.

Note that full information on the approval of the study protocol must also be provided in the manuscript.
