# Supplementary material for: Enhanced lysosomal degradation maintains the quiescent state of neural stem cells
Source: Nat Commun. 2019 Nov 29;10:5446. doi: 10.1038/s41467-019-13203-4 (PMC6884460; doi:10.1038/s41467-019-13203-4)
Supplement: Supplementary file 4 — Description of Additional Supplementary Files [file 41467_2019_13203_MOESM4_ESM.docx]

**Description of Additional Supplementary Files**

File Name: Supplementary Data 1.

Description: Gene expression in NSCs after BafA treatment. Gene expression in NSCs 4 h or 24 h after incubation in normal (cont) or BafA-containing (BafA) quiescence medium was normalized by global normalization (see Methods). Values greater than 100 are highlighted by yellow. Asterisks indicate fold change relative to controls: *2-fold, **4-fold, and ***8-fold. Dollar sign indicates significance, as determined by t-test ($, *P* < 0.05; $$, *P* < 0.01).

File Name: Supplementary Data 2.

Description: Gene expression of Notch- and EGFR-related genes. Notch- and EGFR- related genes were extracted from Supplementary Data 1. Asterisks indicate fold change relative to controls: *2-fold, **4-fold, and ***8-fold.

File Name: Supplementary Movie 1.

Description: Live-cell imaging of NSCs expressing wild type of TFEB-GFP. Images of bright field and GFP (green) were acquired every 10 min for 24 h. Merged images are represented at 10 frames per sec. Red arrows and numbers (red) indicate the positions of dividing cells and frame numbers, respectively.

File Name: Supplementary Movie 2.

Description: Live-cell imaging of NSCs expressing S210A mutant of TFEB-GFP. Images were acquired and represented same as Supplementary Movie 1.
