# Supplementary material for: Enhanced lysosomal degradation maintains the quiescent state of neural stem cells
Source: Nat Commun. 2019 Nov 29;10:5446. doi: 10.1038/s41467-019-13203-4 (PMC6884460; doi:10.1038/s41467-019-13203-4)
Supplement: Supplementary file 8 — Source Data [file 41467_2019_13203_MOESM8_ESM.zip › 183148_3_data_set_4144222_pz8vk6.pdf]

Figure 1g

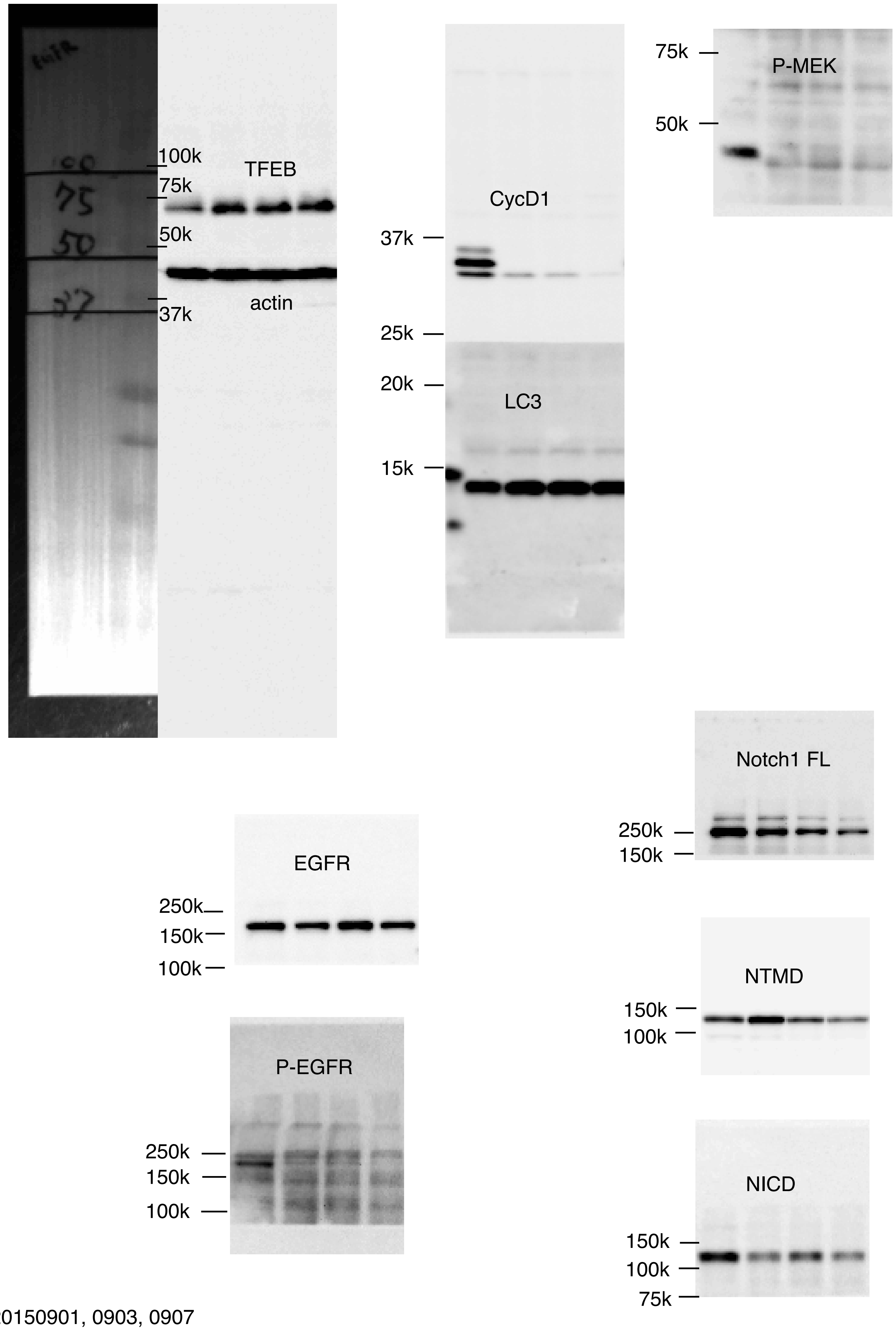

20150901, 0903, 0907

Figure 1h

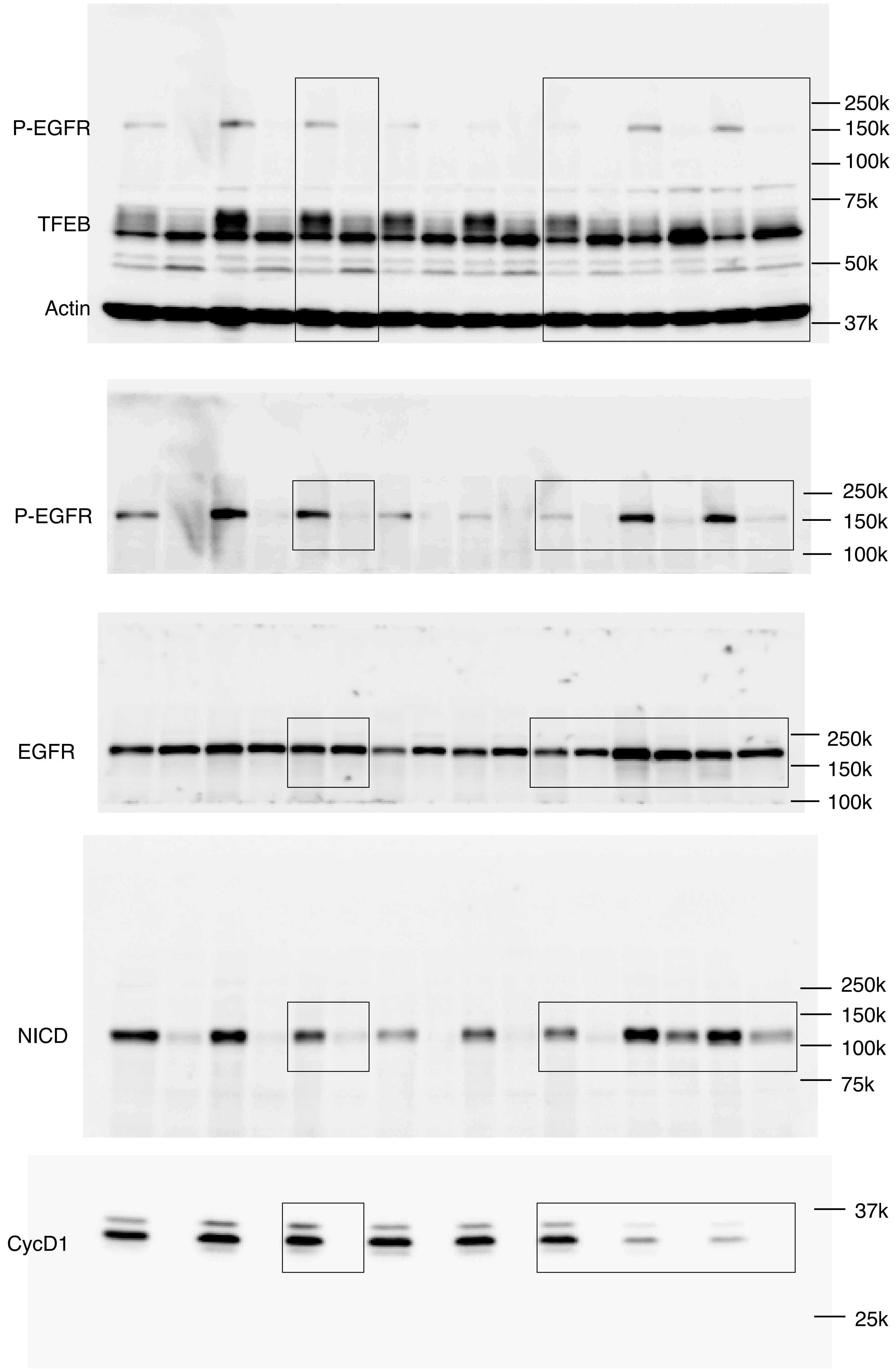

20170602

Figure 2d

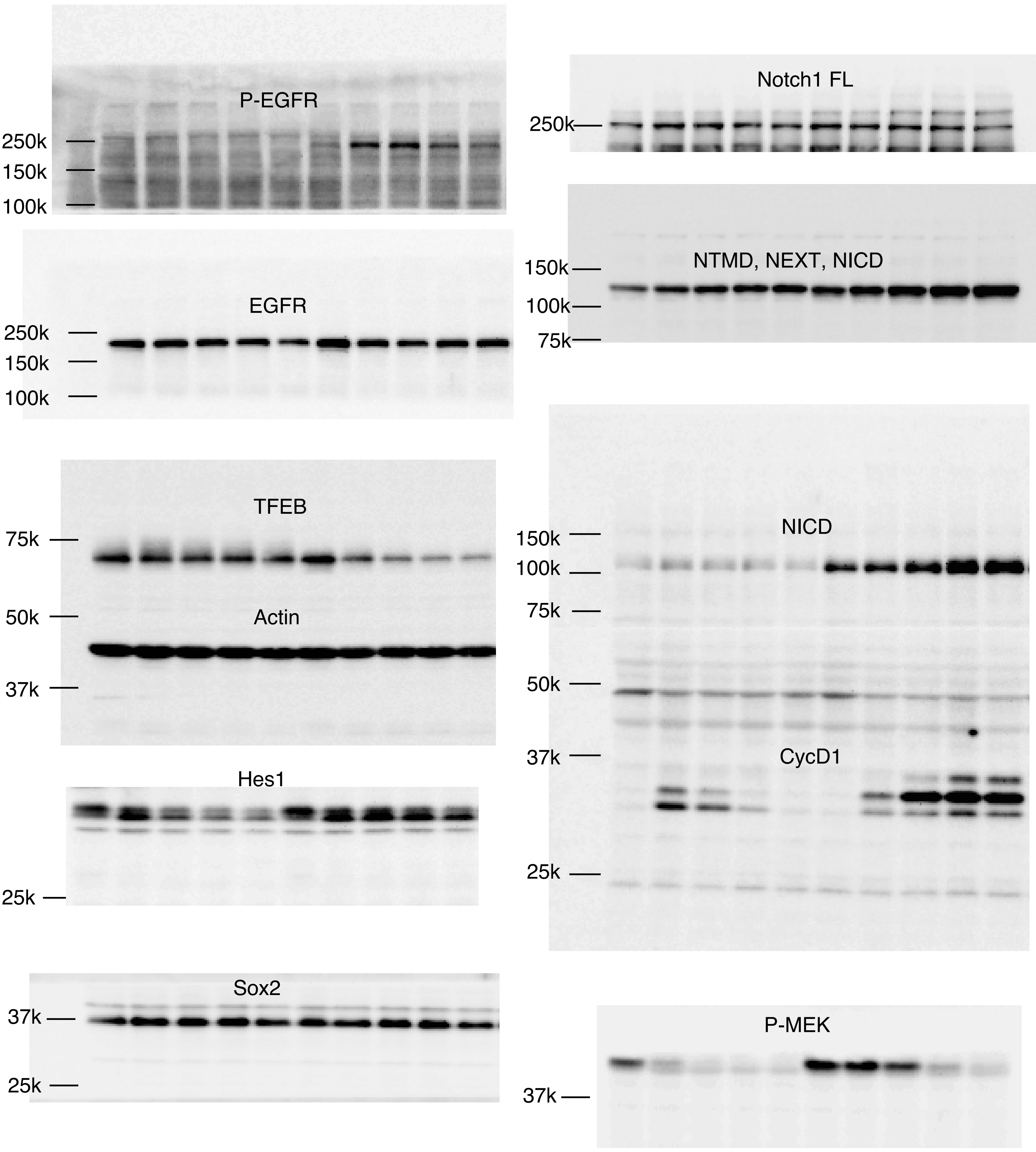

20150818

Figure 2f

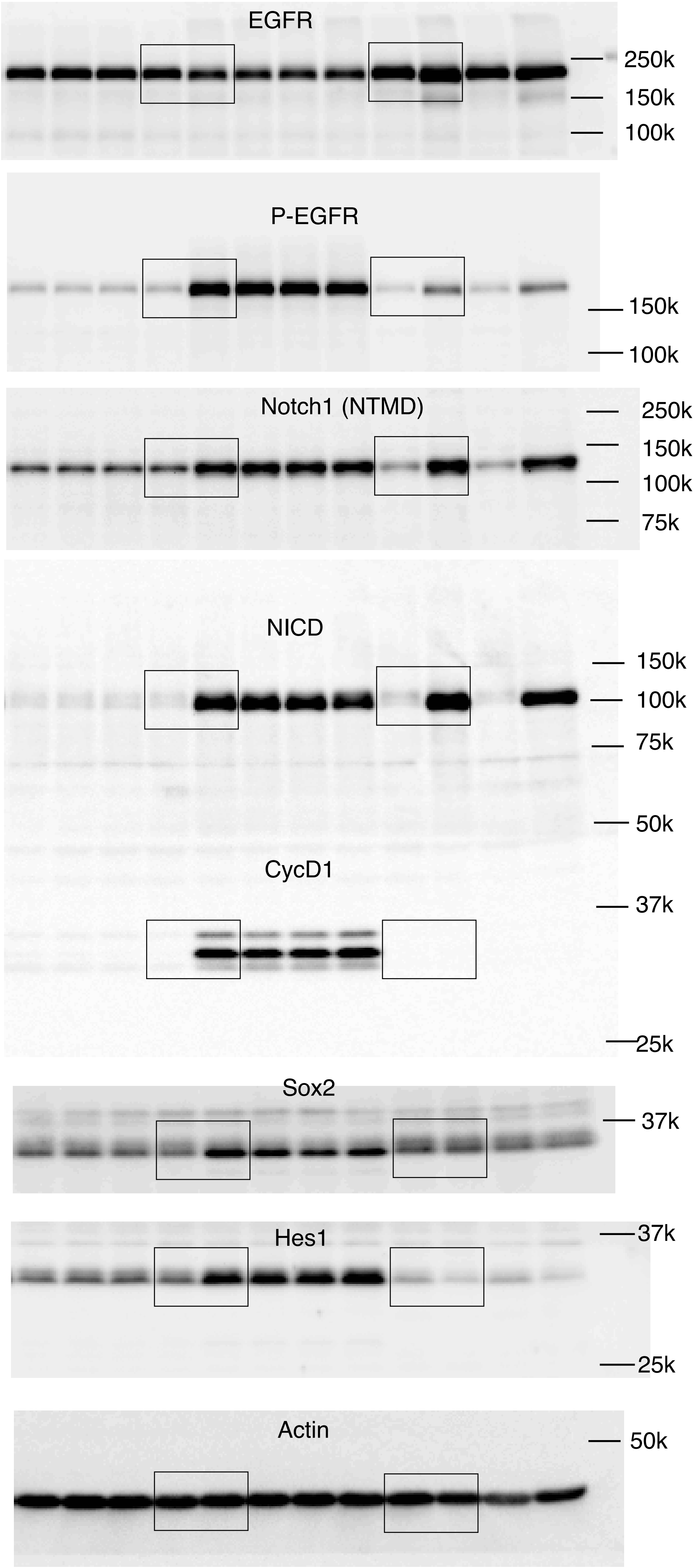
EGFR
250k
150k
100k
P-EGFR
150k
100k
Notch1 (NTMD)
250k
150k
100k
75k
NICD
150k
100k
75k
50k
CycD1
37k
25k
Sox2
37k
Hes1
37k
25k
Actin
50k

Figure 3c

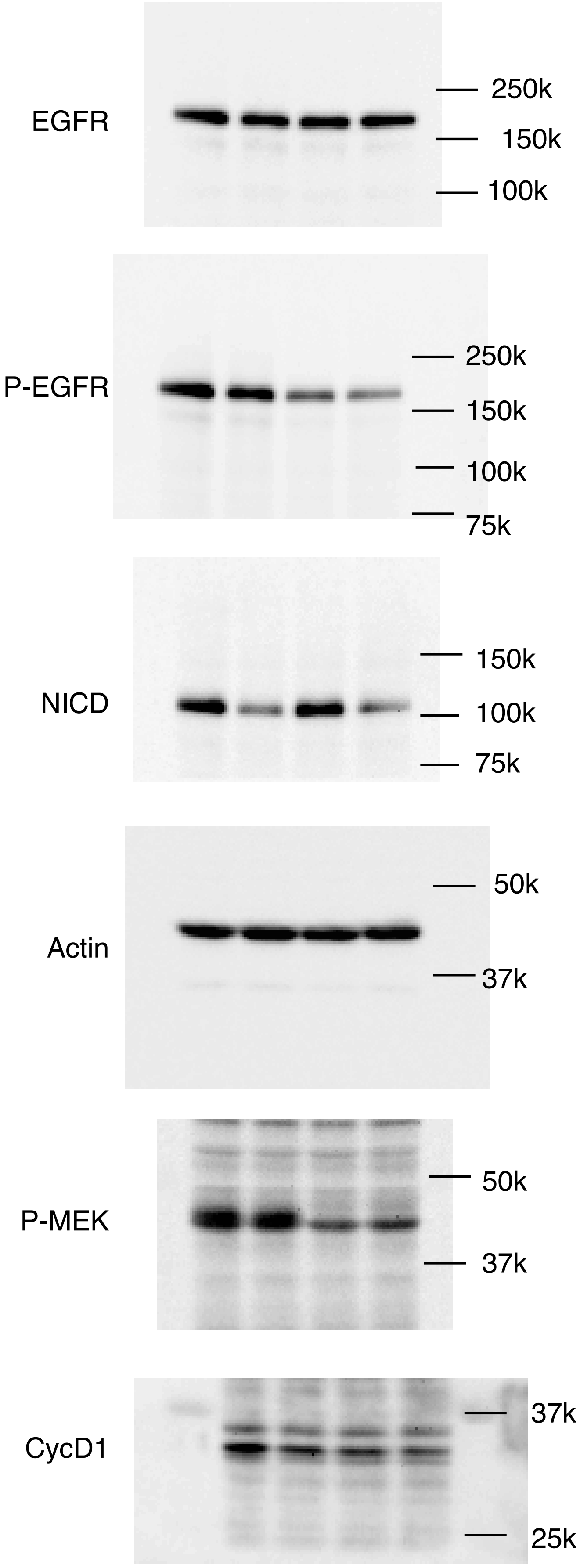

201501125

Figure 3e

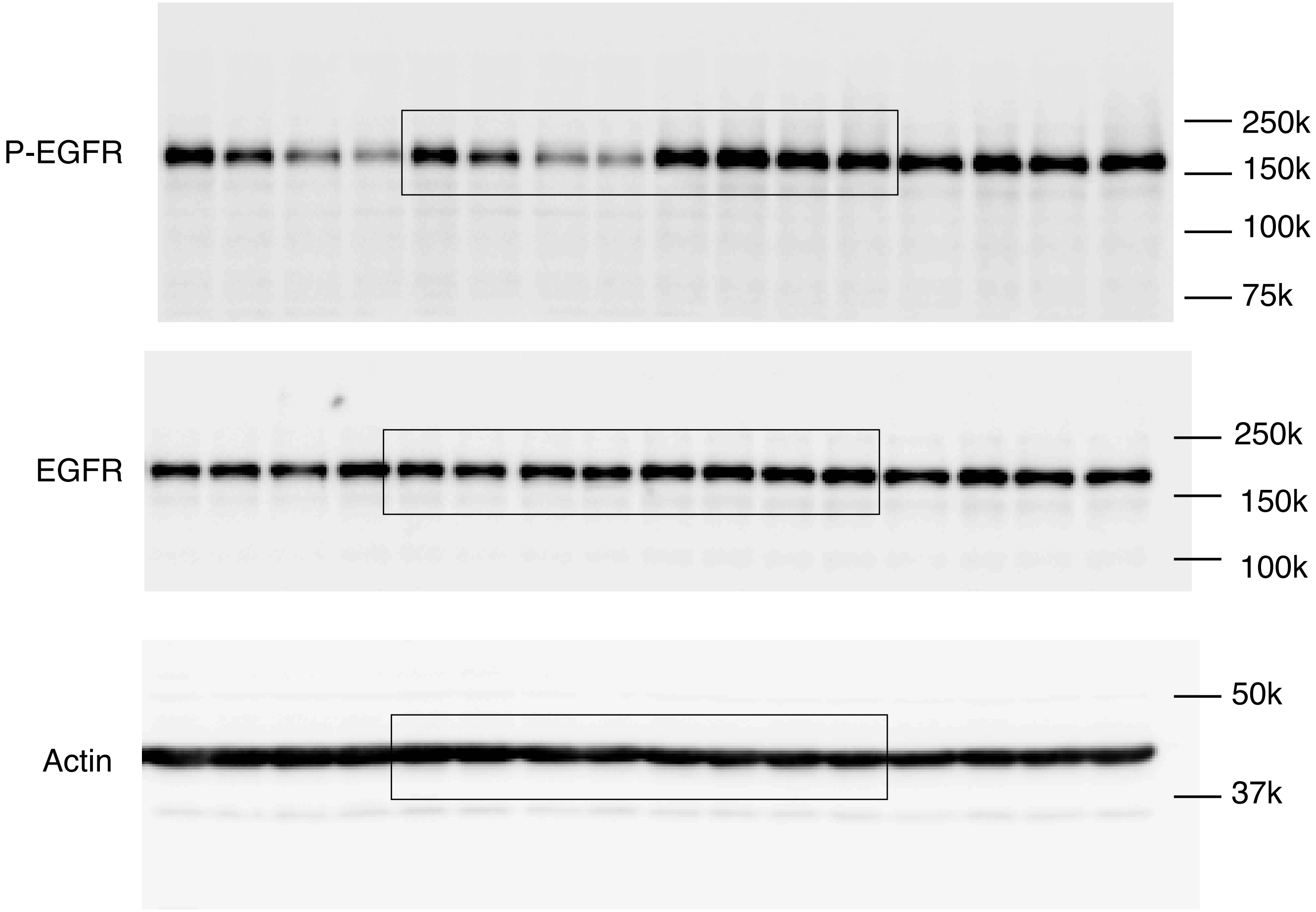

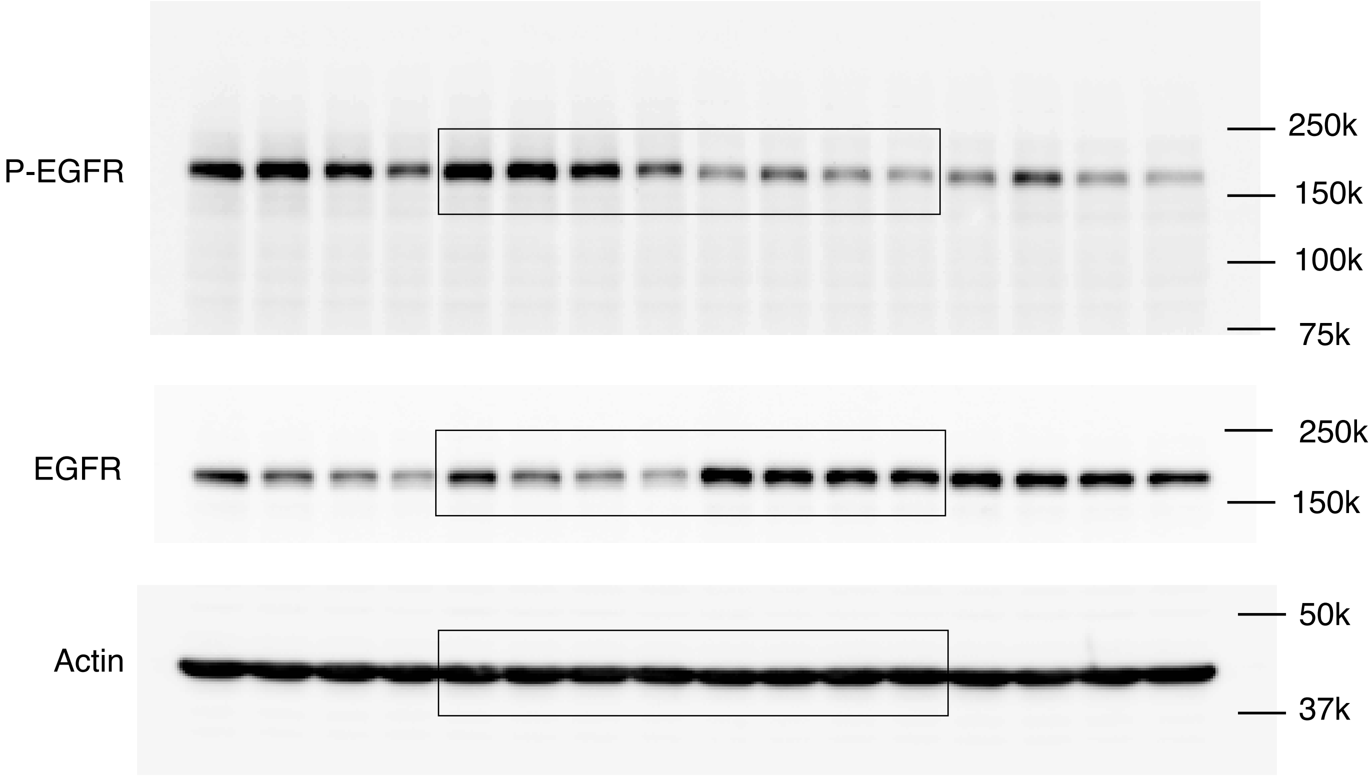

20151023, 1026, 1102

Figure 5a

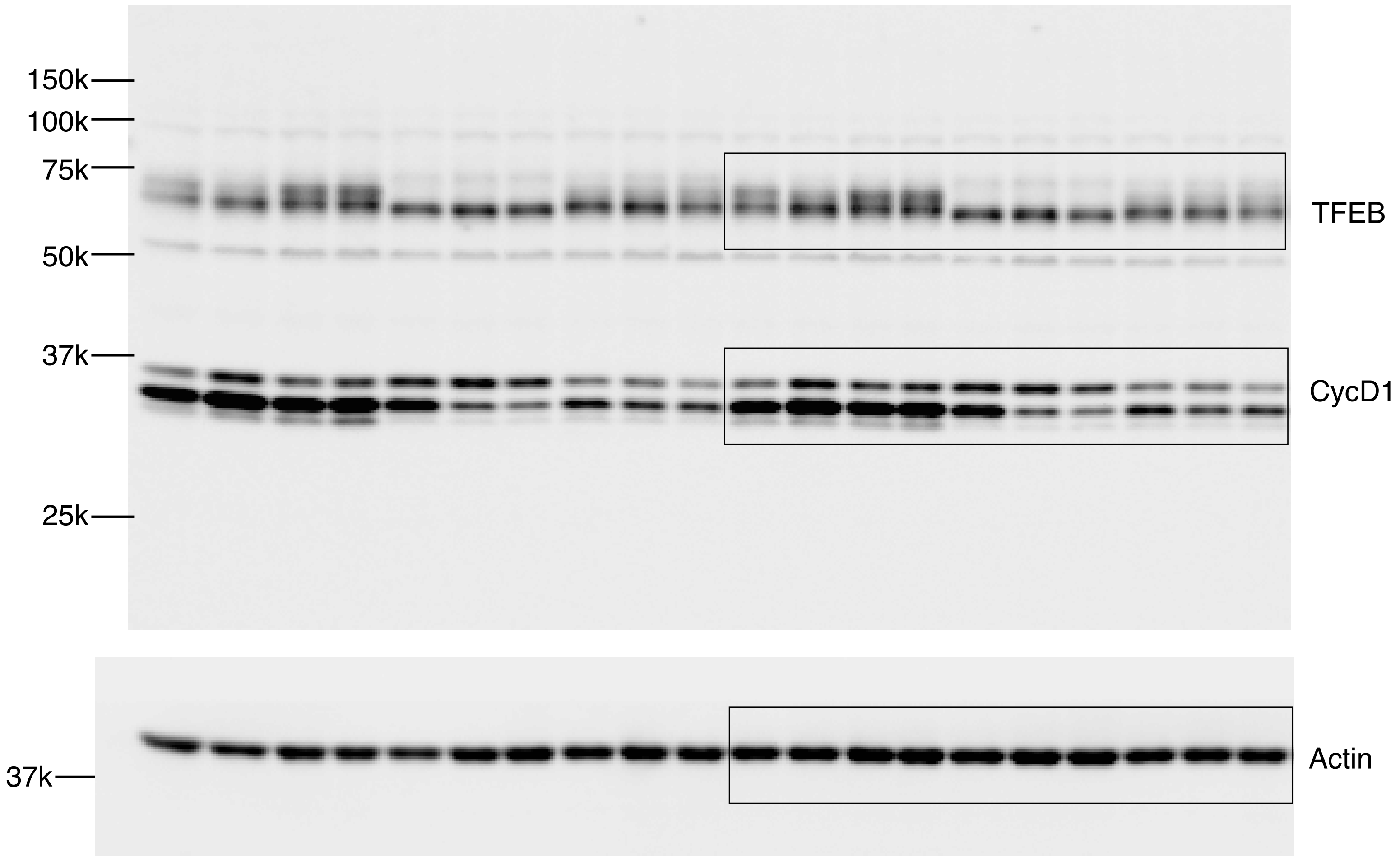

Figure 5d

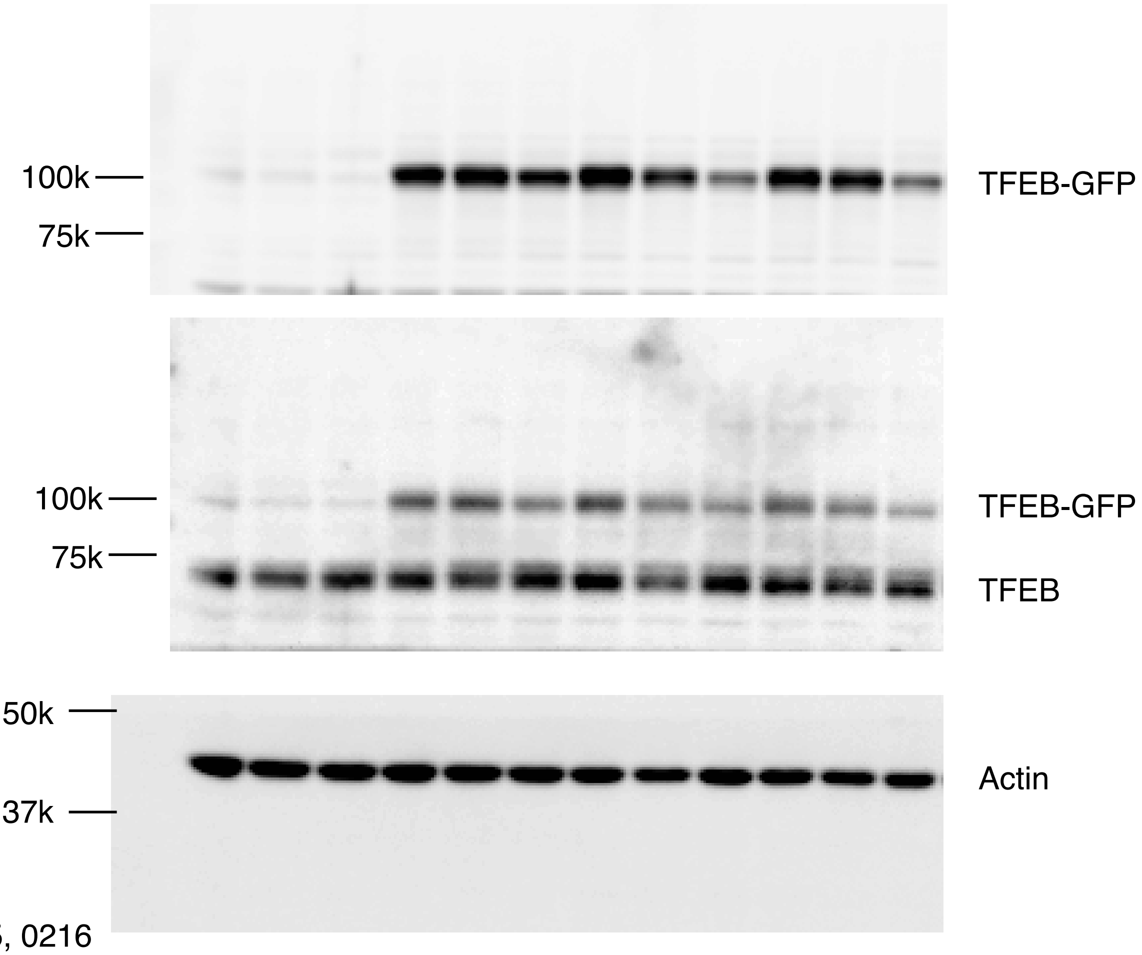

20190215, 0216

Figure 6a

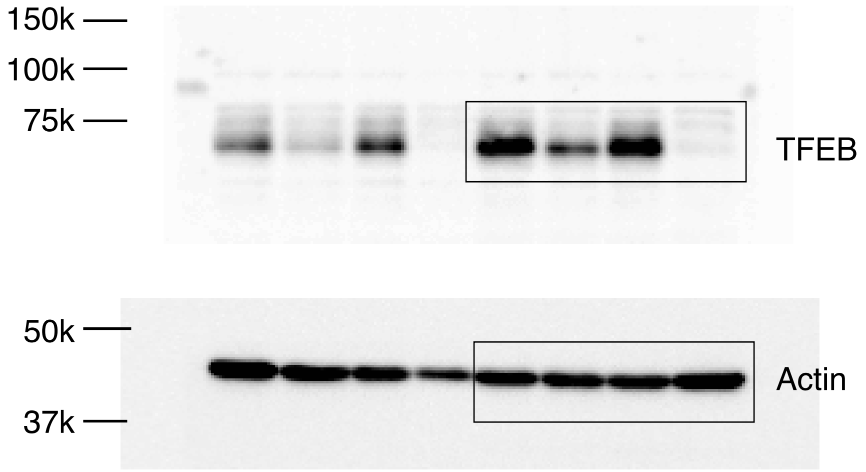

20180608

Figure 6g

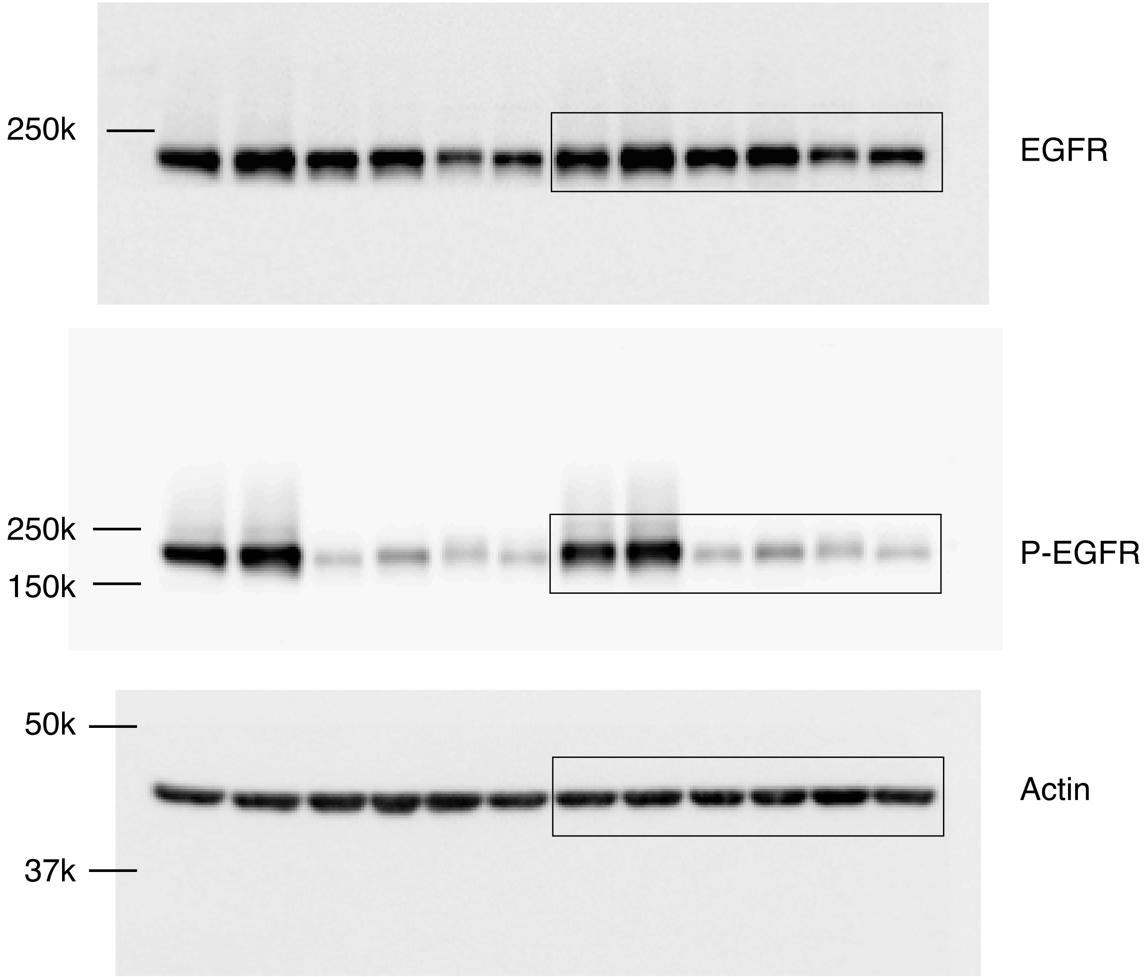

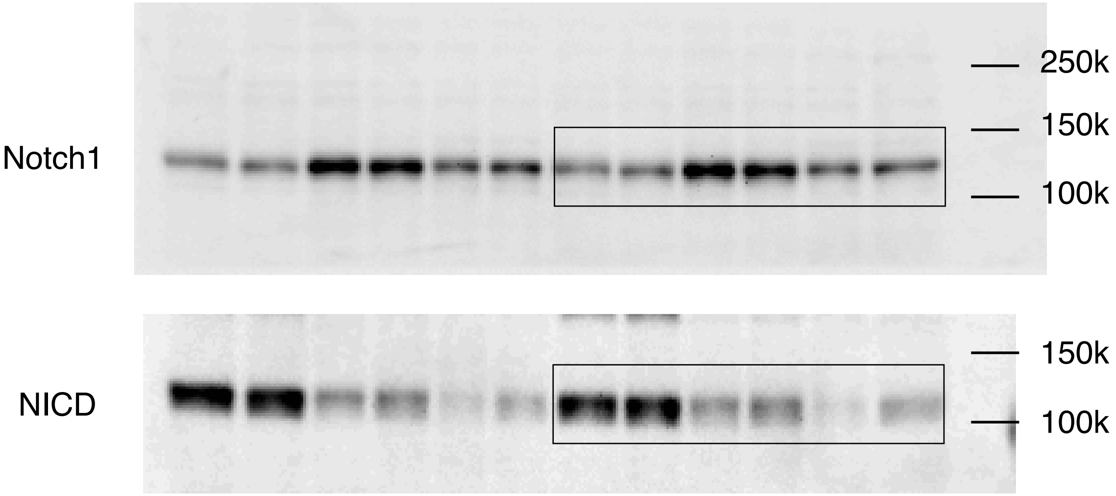

20190118, 0119

Figure 6i

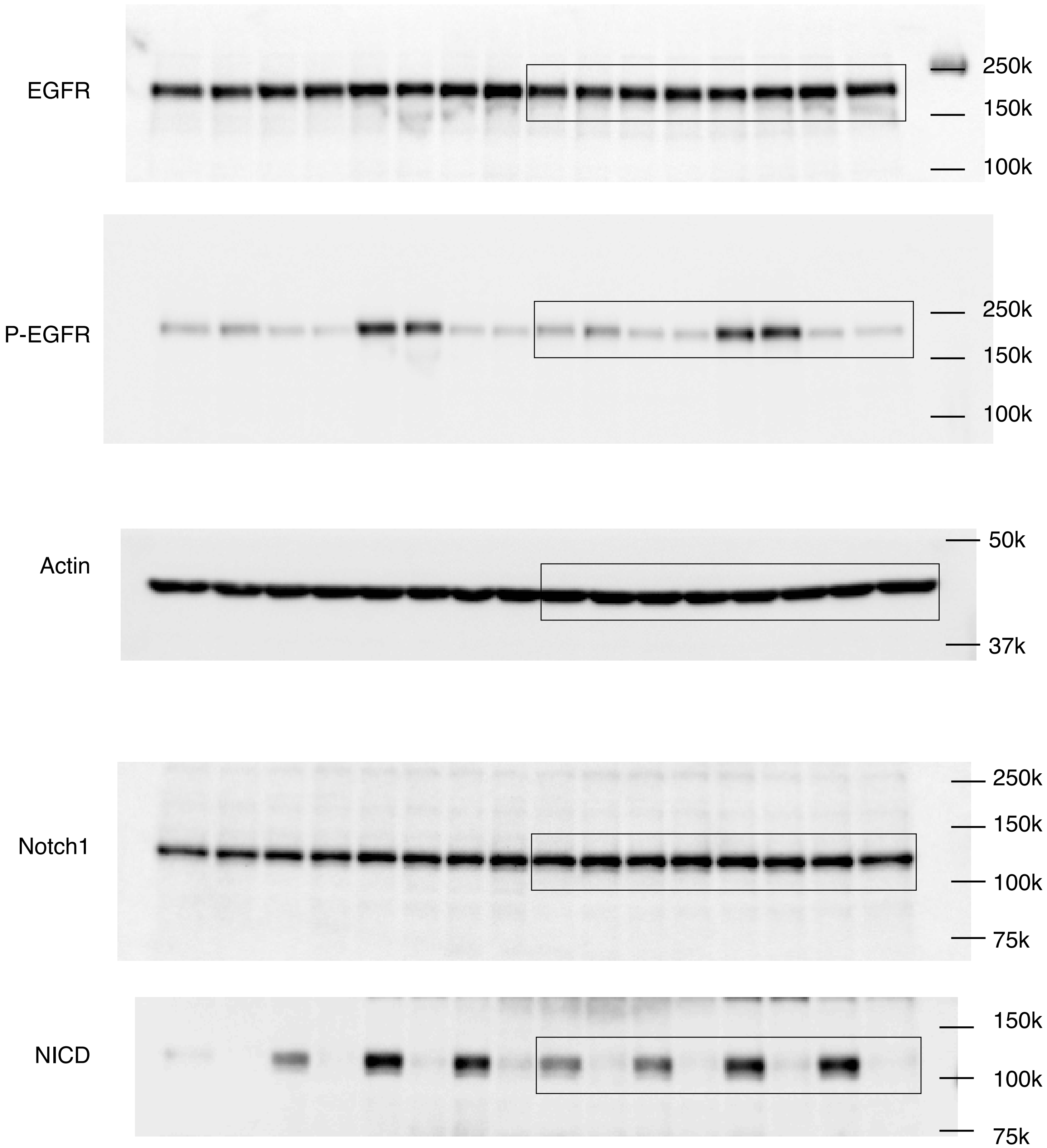

20190316, 0317

Supplemental Figure 1c

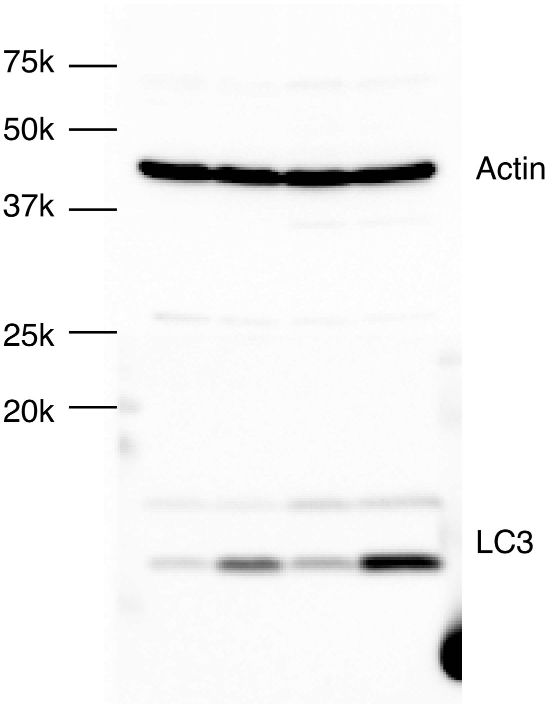

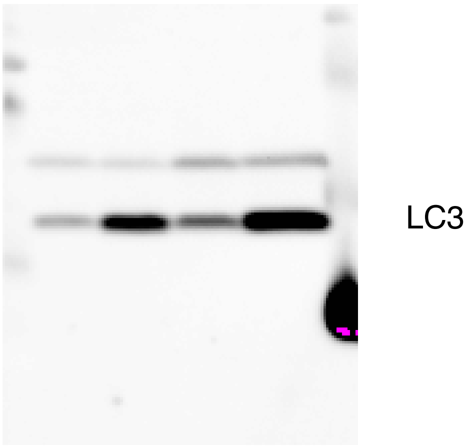

20170612

Supplemental Figure 2e

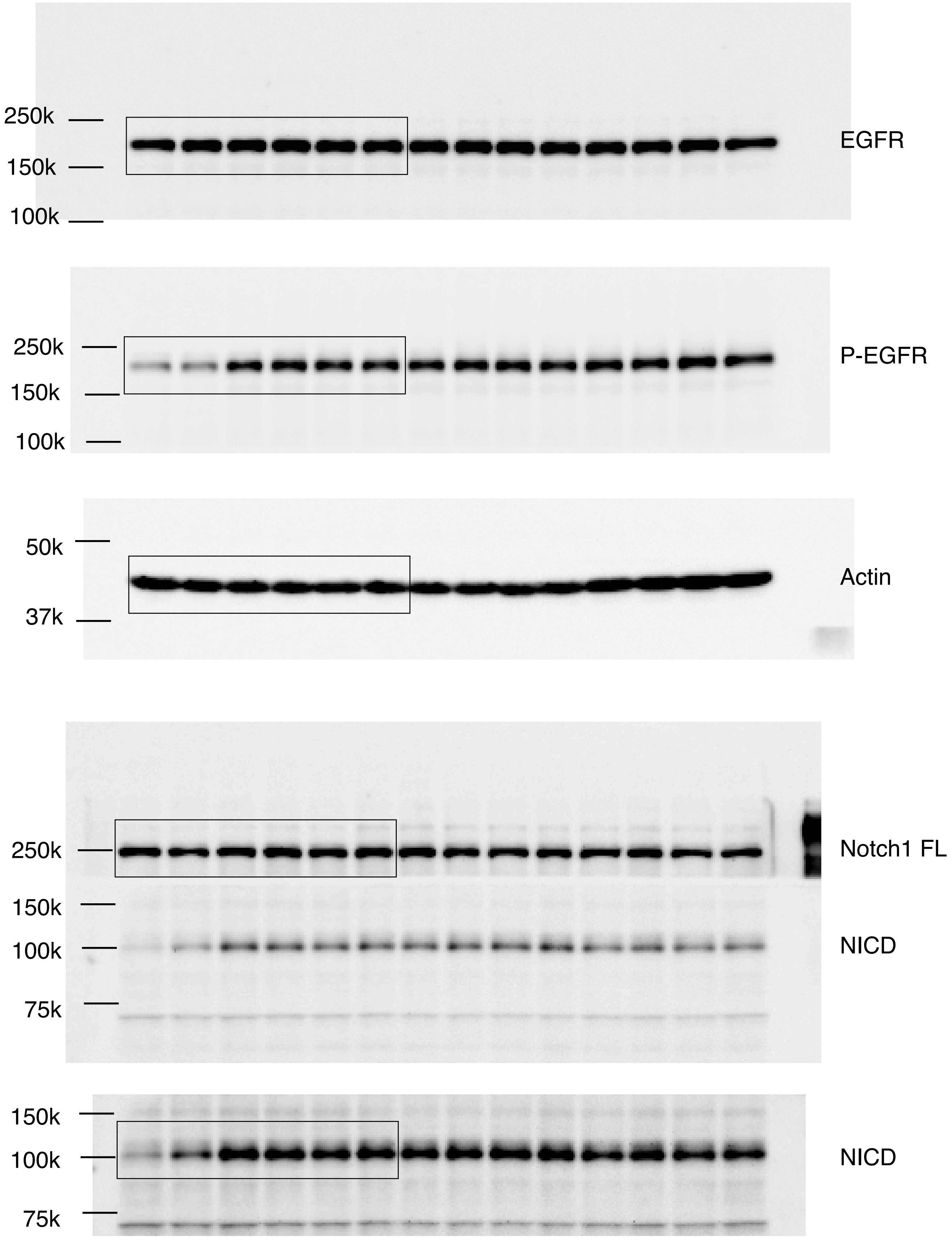

20150910, 0915, 0930

Supplementary Figure 2g

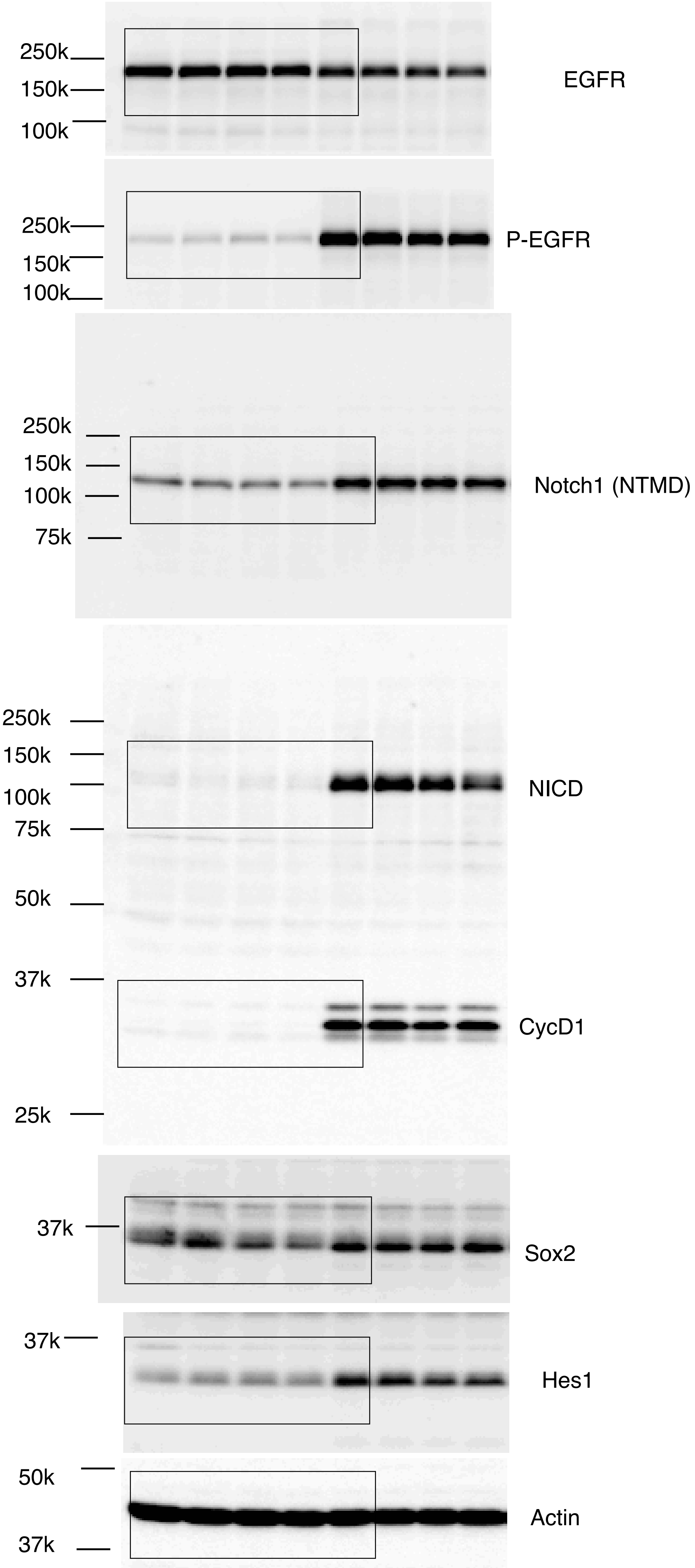

20180309, 0310

Supplementary

Figure 2h

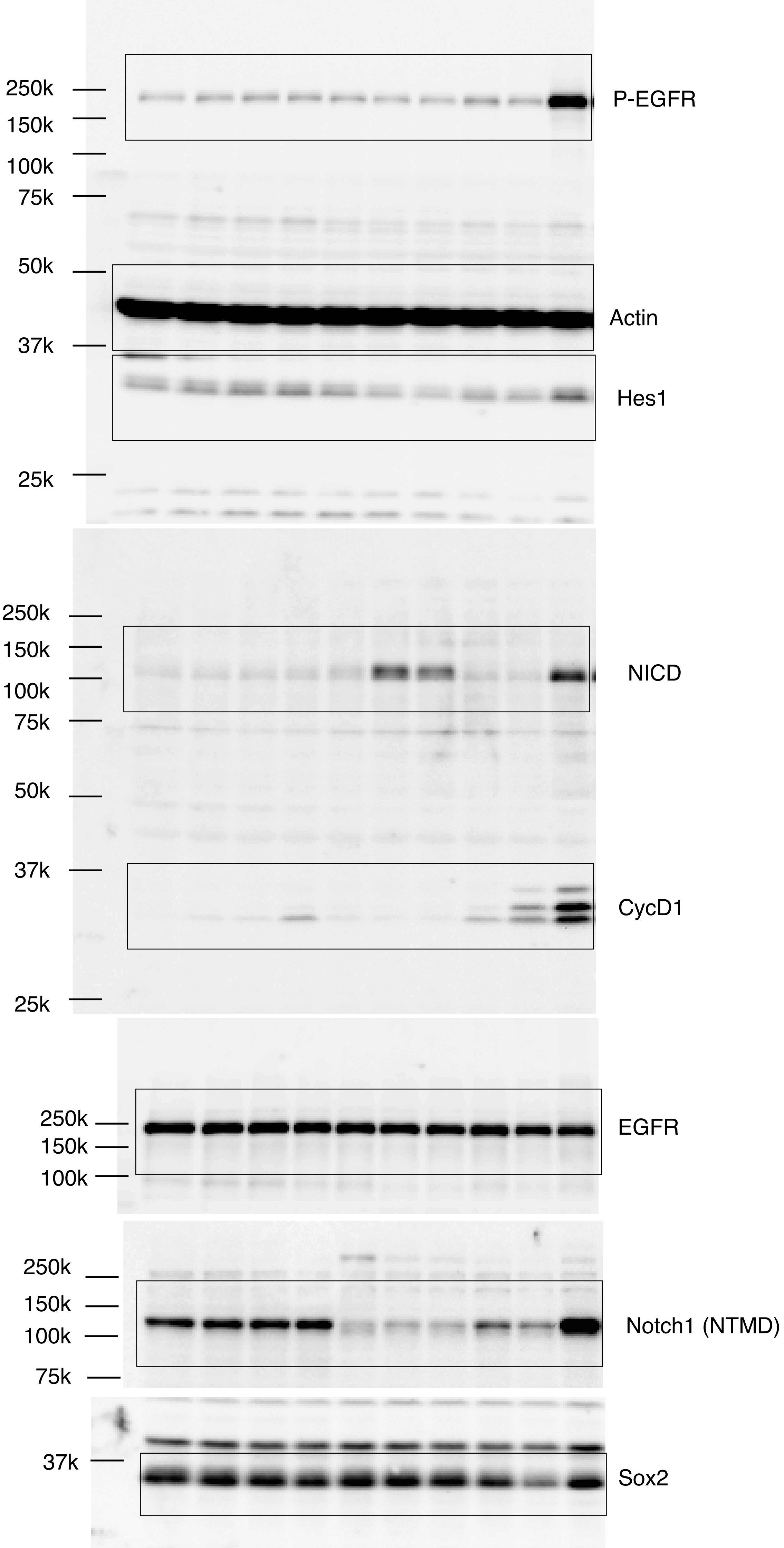

20180203, 0205, 0206

Supplementary Figure 2j

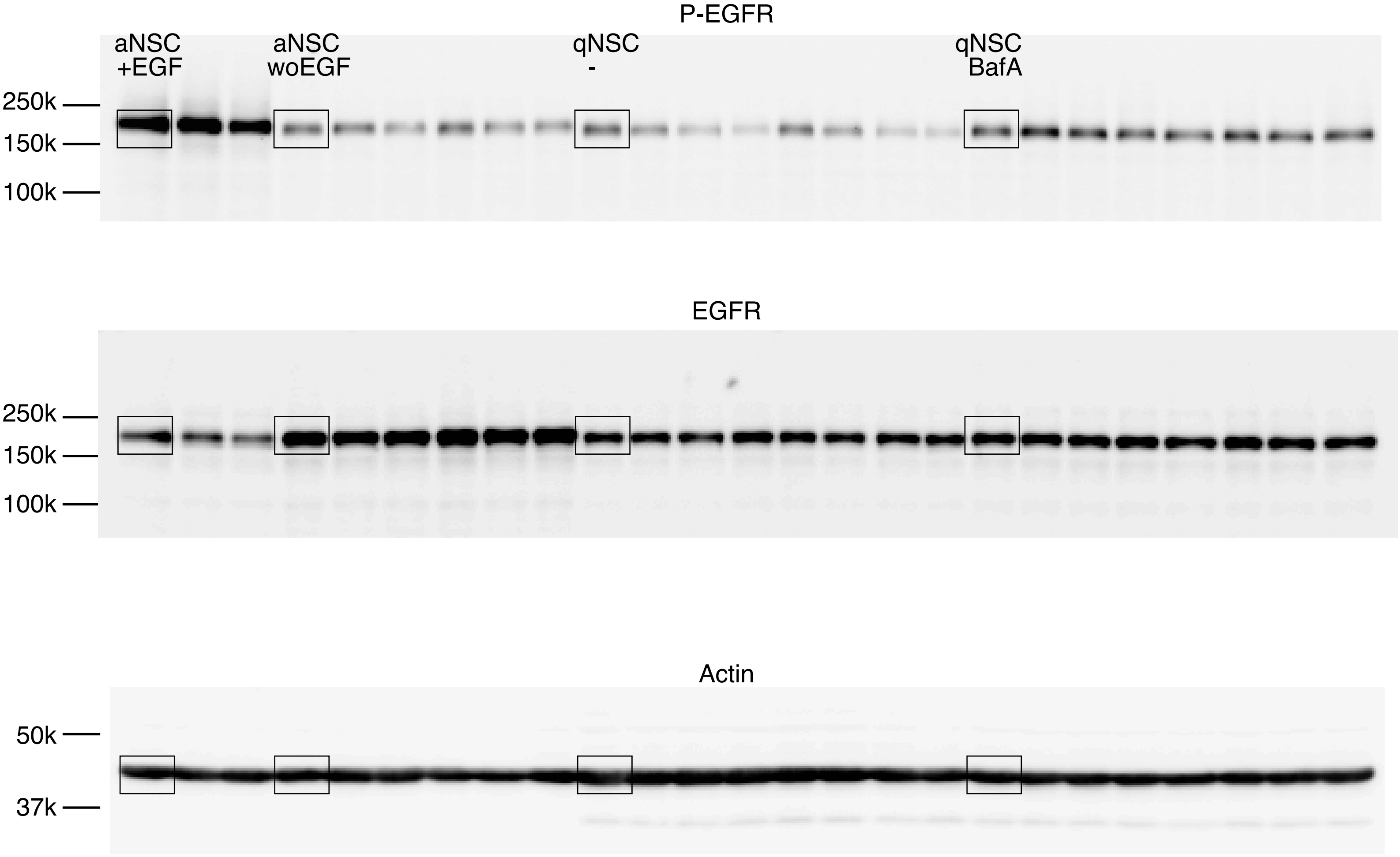

20151023, 1026

Supplementary Figure 3

b

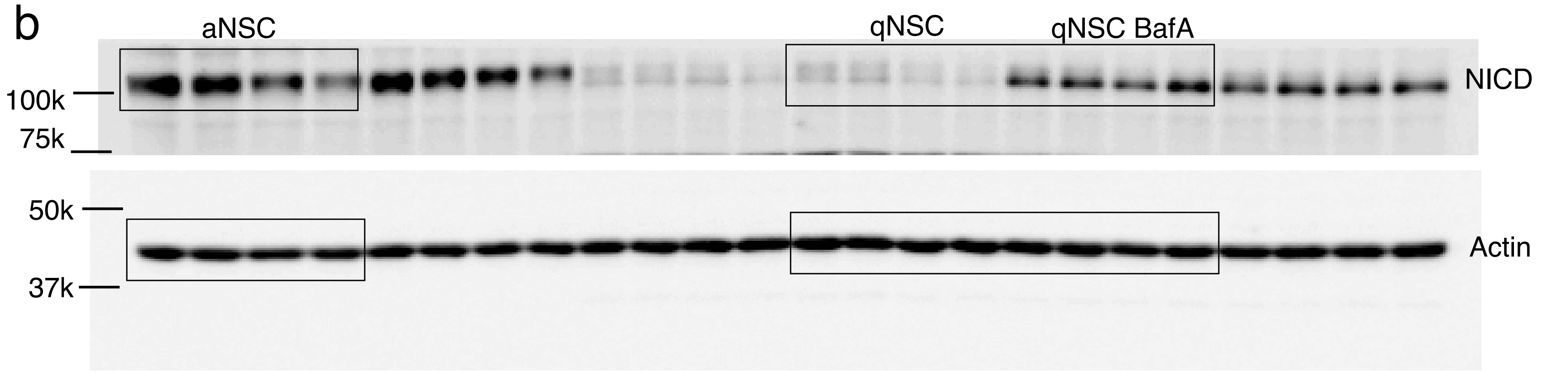

20151125, 1126

c

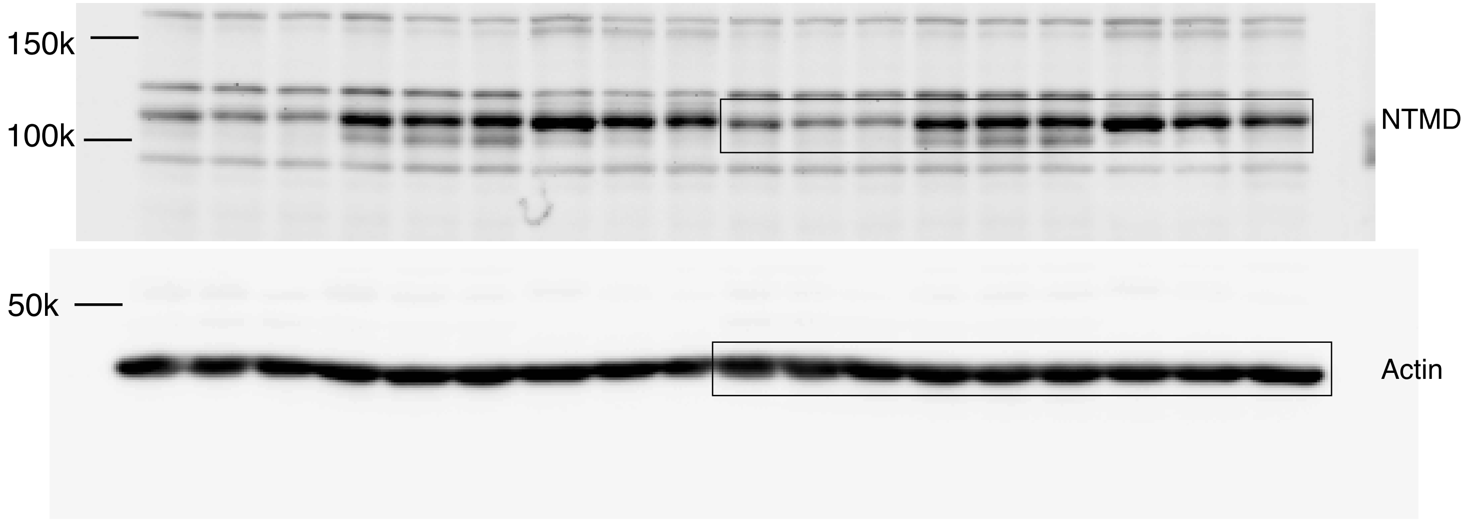

20160906

d

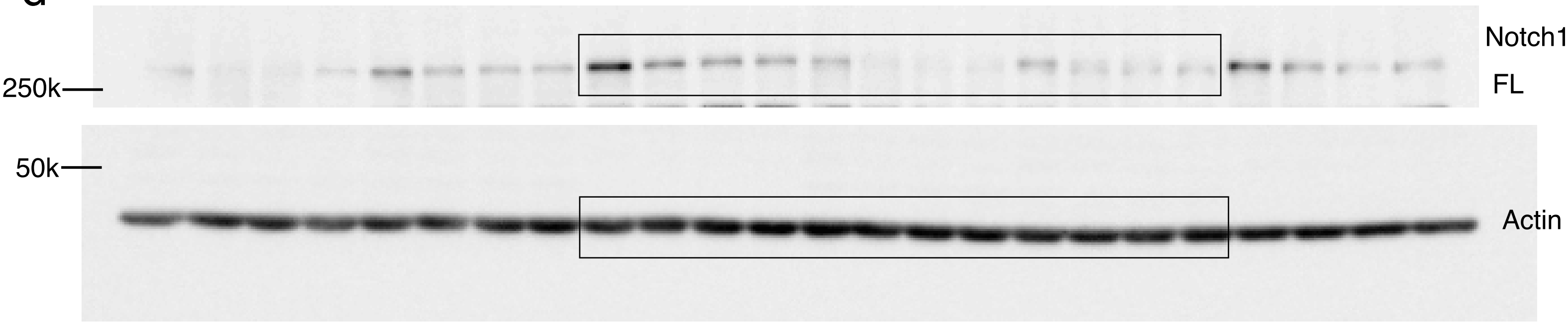

20160826

Supplementary Figure 8a

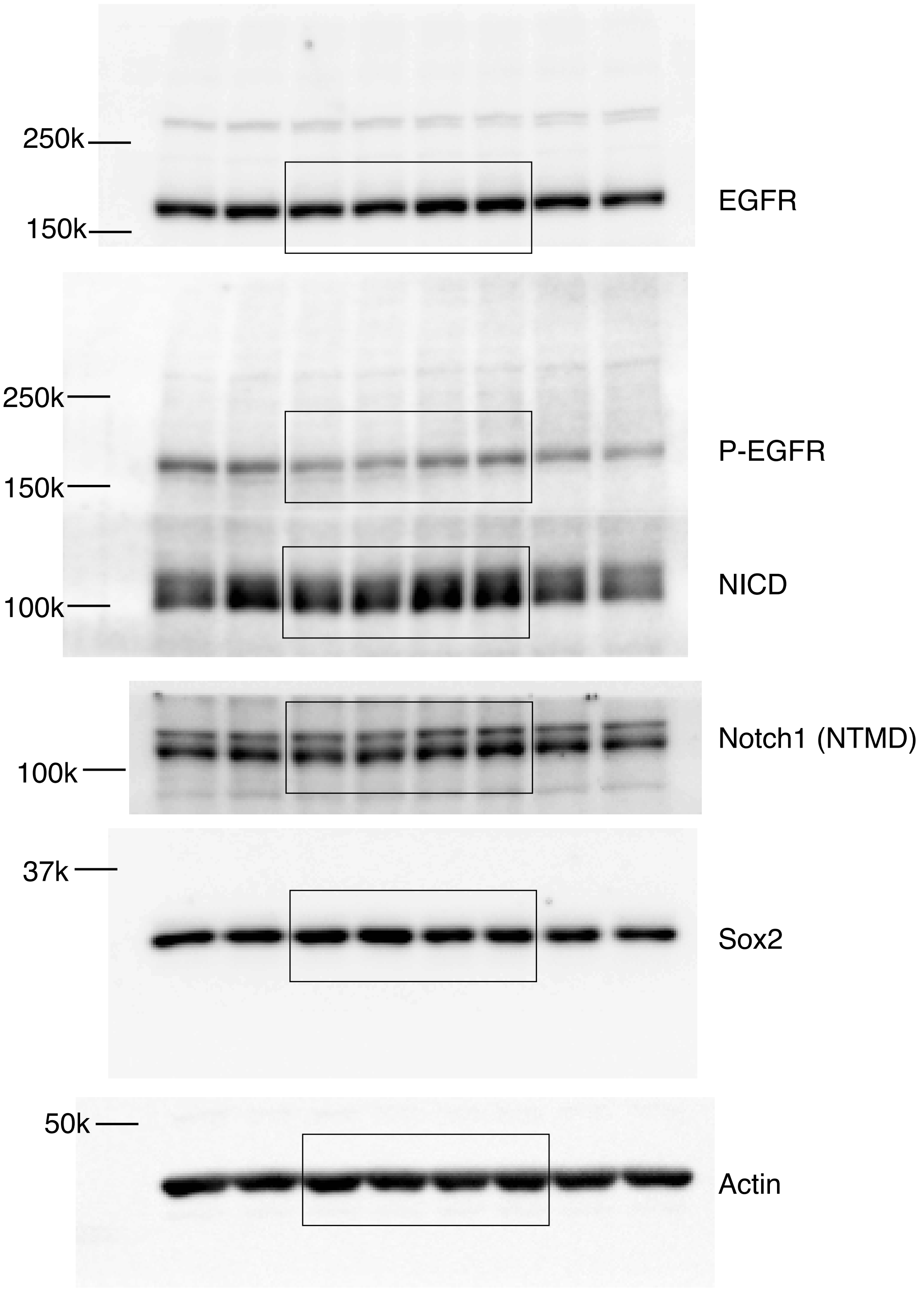

20190718, 0720, 0723
