# Supplementary material for: Enhanced lysosomal degradation maintains the quiescent state of neural stem cells
Source: Nat Commun. 2019 Nov 29;10:5446. doi: 10.1038/s41467-019-13203-4 (PMC6884460; doi:10.1038/s41467-019-13203-4)
Supplement: Supplementary file 2 — Peer Review [file 41467_2019_13203_MOESM2_ESM.pdf]

Reviewers' comments:

Reviewer #1 (Remarks to the Author):

The manuscript by Kobayashi and colleagues addresses the question of how lysosomes function to maintain neural stem cells in a quiescent state. The authors show that lysosomal activity is elevated in an in vitro model of quiescence, and that a function of the lysosmal pathway in these cells is to degrade the activated EGF receptor. Pharmacological inhibition of lysosomal function in vitro or in an in vivo slice assay increased markers of proliferation in qNSCs, supporting a role for this pathway in restraining NSC activation. The authors also explore the role of the TFEB transcription factor and "master regulator" of lysosomal biogenesis in the maintenance of quiescence. They observe that TFEB protein is slightly more nuclear and more transcriptionally active in qNSCs compared to aNSCs. Intriguingly, TFEB knockout NSCs display decreased lysosomal activity in vitro and slightly elevated activation in the quiescent state in vitro and in vivo, but paradoxically decreased neurogenesis in vivo.

Overall, the experiments in this study are well-performed and provide solid evidence for a key role for lysosomes in supporting the quiescent state. A recent study demonstrated this phenomenon as well (Leeman et al., Science 2018). Thus, the novelty in this study lies in the identification of the mechanism through which lysosomes maintain quiescence. The authors propose a mechanism involving the degradation of activated EGFR and transcriptional activity of TFEB, specifically in the quiescent state. Overall, the proposed mechanism is supported by the experimental evidence presented. However, there are key points that need to be addressed to strengthen the model before publication:

A major concern with the study is the subtle increased in proliferation of qNSCs in response to BafA. Though statistically significant (Fig. 3C), the increase in EdU+ and Ki67+ cells is weak and far from levels found in cells cultured in proliferation media. In contrast, activation of signaling pathways that support NSC activation (EGF, Notch) is relatively strong. These seemingly contradictory results raise the question of why the cells are restrained from stronger activation in this assay? A previous study using a similar system showed that BMP signaling is dominant over EGF signaling in vitro, which could explain this result (Joppe, Front Neurosci, 2015). This finding also suggests that the BMP-induced quiescence assay is not an optimal assay to test the proposed model. Similarly, the TFEB KO displayed only a slight increase in Ki67+ cells in the quiescent state. The authors show that TFEB is not required for NSCs to enter quiescence, at least in the case of BMP-induced quiescence (Fig. 6F). The study would be more compelling if the authors could provide stronger evidence perturbing lysosomal activity can regulates NSC quiescence vs activation. For example, does activating lysosomal activity (pharmacologically or through TFEB activation) enhance quiescence?

A second question raised by the study is the nature of the connection between TFEB activity and the pathways that drive NSC activation. Do the TFEB KO have increased pEGFR and NICD? Can the TFEB KO phenotype be suppressed by EGFR and Notch inhibitors?

The reason for the decreased neurogenesis in the TFEB KO is unclear but suggests a role for lysosomal biogenesis in differentiation. Do the TFEB KO cells have differentiation defects in in vitro assays? Is TFEB more nuclear in differentiating NSCs? This could be tested.

The study includes a microarray experiment, but the authors do not include any quality control results from the experiment, and only limited, select results from the experiment are shown. Complete results from this experiment (including QC analysis and a gene list of significant genes) should be included. In addition, are any genes downstream of the EGFR or Notch altered in the microarray analysis?

Minor points:

The study begins with experiments testing various drugs inhibiting peptidase activities and the authors find that the cathepsin inhibitor blocked trypin-like activity in qNSCs. Does this drug increase qNSC activaition (EdU incorporation, Ki67 activity)?

6 month old mice are not “old”. They are adult mice, but not aged.

Figure 3: Few methodological details are provided. How many days after replacing with PM did they perform the Ki67 and EdU incorporation? What exactly is the control timecourse in 3D? In 3F, is the left side of the blot BafA + switch to proliferation medium?

In vivo, EGFR is used to differentiate between qNSCs and aNSCs. In Fig. 4E, it appears that qNSCs (BMP-treated embryonic NSCs?) have equal EGFR levels to aNSCs at 0 time point. Quiescence induction was done in the absence of EGF, so why do qNSCs and aNSCs show similar phosphorylated and total EGFR before cycloheximide? Can the authors clarify?

The manuscript would benefit from the addition diagram that summarizes the main findings and integrates them into a cohesive model at the end (including Notch/ BafA/EGFR/TFEB).

Reviewer #3 (Remarks to the Author):

The paper by Kobayashi et al makes the interesting suggestion that regulation of lysosomal activity is key to the transition between quiescent and active neural stem cells. While many of the data are highly suggestive, in the current state the evidence remains preliminary.
1. The main concern is that most work involves NSCs in culture, and the in vivo work is rather limited. It is clear that mechanisms are more easily studied in vitro, but there should be more attempts to validate conclusions in vivo, e.g. with regard to EGFR and Notch receptor accumulation as well as TFEB regulation. Can BafA be applied to the neurogenic niches in vivo?
2. Also much of the study is based on pharmacology, with some of the drugs exerting toxic effects according to the authors. There should be attempt to use molecular tools to abrogate some of the lysosomal functions.
3. Moreover, it would be very informative to see what happens if lysosomal activity is raised in active NSCs. Can they be converted into qNSC?
4. Some of the western blot experiments seem to lack quantification and statistical analyses.
5. in Figure 3E and F, EGFR expression differences seem to be more pronounced in the immunostaining than the western blot.
6. When discussing the effect of DAPT, do the authors consider also Notch2? In contrast to Notch1, Notch2 may be involved in signaling quiescence.
7. Some of the cell biological experiments, e.g. those describing EGFR and P-EGFR turn over are difficult to follow to the authors' conclusions (page 7, lines 192-200). Eventually, when complex scenarios are being suggested, outcomes might be best modelled mathematically.
8. Some of the immunostainings are not very impressive, including those of TFEB. What are the controls for the specificity of the antibodies used?
9. What is the contribution of an acute injury response to the slice culture experiments
10. The in vitro experiments using the TFEB flox/flox cells are not well described? How is Cre introduced and transiently activated?

11. The in vivo effects of TFEB ko are not studied in sufficient detail. (A control wildtype image should be provided alongside Fig 6A). The fact that the reasons for the lack of increased neurogenesis following higher levels of aNSC is not pursued is unsatisfactory. It would be important to include more time points to reveal the dynamics of NSC activation, such as 7days, 14 days, 1 month, 2 months et al.

12.From the knock out experiments, it does not become clear how TFEB changes NSC activity. The following experiments should be included (1) Use BrdU to quantify cell cycle reentry. (2) Long term BrdU retention to label quiescent NSCs, then knock out TFEB in these labeled quiescent NSCs. (3) BrdU label active stem cells, then knock out TFEB in these labeled active NSCs.

Minor points:

The manuscript is somewhat disorganized. There is multiple switching back and forth with regard to figure numbering. Also, the experimental models used are multiple and sometimes without a clear statement for why a specific model is used rather than another. Some figures may be rather supplemental information, such as Figure 2 upper two panel rows.

We would like to thank the editor and the reviewers for their valuable and constructive comments. We answered their comments one by one as below.

To the Editor:

In particular, we need to see both reviewers concerns regarding the validation of these findings in vivo (with regard to EGFR and Notch receptor accumulation as well as TFEB regulation) addressed completely (Rev#2 pt 1; Rev#1).

To answer this comment, we have conducted immunohistochemistry (IHC) to quantify levels of EGFR, Notch1 and Notch2 receptors in NSCs of TFEB-cKO mice. Results showed that the levels of the Notch1 receptor were slightly elevated in the SGZ of adult *Tfeb*-cKO mice (Supplemental Fig. 6c). Alternatively, we analyzed postnatal *Tfeb*-cKO mice at P0 stage. In P0 mice, levels of the Notch1 protein intensity significantly increased in the DG of *Tfeb*-cKO mice than wild-type mice (Supplemental Fig. 6f).

Furthermore, we expect this study to be further strengthened with the inclusion of experimental data, beyond the use of pharmacological inhibitors, to demonstrate the effects of lysosomal functions on NSC dynamics (Rev#2 pt 2).

We analyzed the expression of EGFR, Notch1 and Notch2 receptors in TFEB-KO NSC *in vitro*. Our data revealed a significant accumulation of active EGFR and Notch1 in TFEB KO relative to control, as observed following treatment with BafA. Data are shown in Fig. 6g-j.

To the Reviewer #1:

A major concern with the study is the subtle increased in proliferation of qNSCs in response to BafA. Though statistically significant (Fig. 3C), the increase in EdU+ and Ki67+ cells is weak and far from levels found in cells cultured in proliferation media. In contrast, activation of signaling pathways that support NSC activation (EGF, Notch) is relatively strong. These seemingly contradictory results raise the question of why the cells are restrained from stronger activation in this assay? A previous study using a similar system showed that BMP signaling is dominant over EGF signaling in vitro, which could explain this result (Joppe, Front Neurosci,

2015). This finding also suggests that the BMP-induced quiescence assay is not an optimal assay to test the proposed model. Similarly, the TFEB KO displayed only a slight increase in Ki67+ cells in the quiescent state. The authors show that TFEB is not required for NSCs to enter quiescence, at least in the case of BMP-induced quiescence (Fig. 6F). The study would be more compelling if the authors could provide stronger evidence perturbing lysosomal activity can regulates NSC quiescence vs activation. For example, does activating lysosomal activity (pharmacologically or through TFEB activation) enhance quiescence?

We appreciate your comments regarding BMP-induced quiescence. We agree that BMP exerts a strong effect on NSC proliferation. For example, BMP rapidly decreased the level of cyclin D1 in aNSCs within 4 hours in our experiment (not shown), but this might still be the only method for inducing NSC quiescence *in vitro*. We very much appreciate the suggestion that we perform new experiments to increase lysosomal activity. We conducted two experiments in response to this recommendation; the results are shown in new Fig. 5. First, we treated aNSC with rapamycin and Torin-1, two pharmacological activators of TFEB: rapamycin indirectly blocks mTORC1 activity, whereas Torin-1 directly inhibits both mTORC1 and mTORC2. Both drugs clearly decreased the level of phosphorylated TFEB (therefore they activated TFEB) and reduced the level of cyclin D1 in aNSCs (new Fig. 5a, b). However, both of these drugs are well-known inhibitors of the mTOR pathway, which is involved in EGF-driven proliferation of neural precursor cells (Paliouras et al. JNS, 2012). Second, we exogenously expressed two constitutively active mutants of TFEB (Settembre et al. EMBO J. 2012). Because continuous expression of these mutants under the control of the EF promoter in a construct delivered by a lentiviral vector dramatically decreased the number of TFEB-GFP–expressing cells in culture after several passages (not shown), we generated doxicycline (dox)-inducible lentiviral vectors. The results are shown in new Fig. 5 c-e. Exogenous TFEB mutants were expressed at the similar levels to endogenous or wild-type TFEB in the presence of Dox on 1 day, but their levels gradually decreased on 2 and 3 days (new Fig. 5d). This result suggests that the number of cells expressing mutant TFEB-GFPs relative to that of non-infected cells decreased in culture, similar to the observation using the EF promoter as mentioned above. Consistent with this observation, the proportion of Ki-67–positive cells decreased to a greater extent in NSCs expressing constitutively active mutants than in NSCs expressing wild type on 3 day (new Fig. 5e). These results indicate that TFEB activation (i.e., lysosomal activation) decreases proliferation of aNSCs.

A second question raised by the study is the nature of the connection between TFEB activity and the pathways that drive NSC activation. Do the TFEB KO have increased pEGFR and NICD? Can the TFEB KO phenotype be suppressed by EGFR and Notch inhibitors?

We analyzed pEGFR and NICD in TFEB-KO NSCs in quiescence medium, and found the levels of both proteins increased in TFEB-KO NSCs relative to the control (new Fig. 6 g, h). As you mentioned, these increases were suppressed in the presence of EGFR and Notch inhibitors (new Fig. 6i, j). These results reveal a connection between TFEB activity and the regulation of activated membrane receptors.

The reason for the decreased neurogenesis in the TFEB KO is unclear but suggests a role for lysosomal biogenesis in differentiation. Do the TFEB KO cells have differentiation defects in in vitro assays? Is TFEB more nuclear in differentiating NSCs? This could be tested.

We examined neuronal differentiation *in vitro* by immnuostainig for Tuj-1 and DCX as shown in new Supplemental Fig. 5. The results revealed that neuronal differentiation was slower in TFEB-KO cells than in control cells (new Supplemental Fig. 5a, b). We detected nuclear localization of TFEB on 1 day (new Supplemental Fig. 5c), suggesting that TFEB also plays a role in the initiation of neuronal differentiation.

The study includes a microarray experiment, but the authors do not include any quality control results from the experiment, and only limited, select results from the experiment are shown. Complete results from this experiment (including QC analysis and a gene list of significant genes) should be included. In addition, are any genes downstream of the EGFR or Notch altered in the microarray analysis?

We included all data obtained from our microarray experiment in new Supplemental Table 1 and listed genes related to the EGFR and Notch pathways in new Supplemental Table 2. As shown in Supplemental Table 2, several downstream genes were upregulated in BafA-treated qNSCs.

Minor points:

The study begins with experiments testing various drugs inhibiting peptidase activities and the authors find that the cathepsin inhibitor blocked trypin-like activity in qNSCs. Does this drug increase qNSC activaition (EdU incorporation, Ki67 activity)?

As you mentioned, we treated qNSCs with the cathepsin inhibitor I, the same inhibitor used in Fig. 1a, but it was much more toxic to NSCs than BafA. Complete inhibition of lysosomes seems to be extremely toxic to NSCs.

6 month old mice are not "old". They are adult mice, but not aged.

We corrected the manuscript from "old" to "adult".

Figure 3: Few methodological details are provided. How many days after replacing with PM did they perform the Ki67 and EdU incorporation? What exactly is the control time course in 3D? In 3F, is the left side of the blot BafA + switch to proliferation medium?

We appreciate this comment. We added the details to the legend of new Fig. 2 (original Fig. 3). A day before our analyses, we replaced quiescence medium in all samples: with quiescence medium for control; with quiescence medium containing BafA for the BafA sample; and with proliferation medium for the PM sample. We also added "control" to show the time course in the legend of new Fig. 2d (original Fig. 3D). In new Fig. 2f (original Fig. 3F), quiescence medium (no EGF) was used for all samples.

In vivo, EGFR is used to differentiate between qNSCs and aNSCs. In Fig. 4E, it appears that qNSCs (BMP-treated embryonic NSCs?) have equal EGFR levels to aNSCs at 0 time point. Quiescence induction was done in the absence of EGF, so why do qNSCs and aNSCs show similar phosphorylated and total EGFR before cycloheximide? Can the authors clarify?

We added a new table and a new figure to compare EGFR and P-EGFR levels at time 0 in the same western blot (new Supplemental Fig. 2i, j). P-EGFR level was lower in qNSCs than in aNSCs with EGF, but it was the same in qNSCs as in aNSCs without EGF. As reported previously, EGFR expression is reduced in qNSC (Martynoga et al, Genes & Development, 2013). However, our data including mathematical simulation (new Fig. 3e and new

supplemental text) suggested that total EGFR degradation was repressed in qNSCs due to the absence of EGF, which might be responsible for the equivalent EGFR level in aNSCs to in qNSCs.

The manuscript would benefit from the addition diagram that summarizes the main findings and integrates them into a cohesive model at the end (including Notch/ BafA/EGFR/TFEB).

We added a model diagram to new Supplemental Fig. 7.

To the Reviewer #3:

1. The main concern is that most work involves NSCs in culture, and the in vivo work is rather limited. It is clear that mechanisms are more easily studied in vitro, but there should be more attempts to validate conclusions in vivo, e.g. with regard to EGFR and Notch receptor accumulation as well as TFEB regulation. Can BafA be applied to the neurogenic niches in vivo?

According to this comment, we have conducted IHC for EGFR, Notch1 and Notch2 in TFEB cKO mice. The results are shown in new Supplemental Fig. 6. The levels of the Notch1 receptor were slightly elevated in the SGZ of adult *Tfeb*-cKO mice; however, there was a wide variety in cKO mice relative to in wild-type mice (Supplemental Fig. 6c). Because the IHC signals of these receptors were very weak in adult mice (Supplemental Fig. 6b), we also prepared P0 TFEB cKO mice. In P0 mice, the IHC signals were very clear (Supplemental Fig. 6e) and the levels of the Notch1 protein intensity significantly increased in the DG of *Tfeb*-cKO mice than wild-type mice (Supplemental Fig. 6f).

Previously, we attempted stereotactic injection of BafA into the SGZ via a glass capillary, but the resultant damage (including the injection itself) in lysosomes made it impossible to detect significant differences relative to control samples. Alternatively, we performed the slice culture experiments (new Fig. 4c-g).

2. Also much of the study is based on pharmacology, with some of the drugs exerting toxic effects according to the authors. There should be attempt to use molecular tools to abrogate some of the lysosomal functions.

According to your suggestion, we used TFEB-KO NSCs as a tool to abrogate activation of lysosomes in the quiescent state. We observed significant accumulations of activated EGFR and Notch1 receptor in TFEB-KO NSCs, as in BafA-treated cells (new Fig. 6g, h).

3. Moreover, it would be very informative to see what happens if lysosomal activity is raised in active NSCs. Can they be converted into qNSC?

We appreciate this comment. We increased lysosomal activity in two ways: pharmacological activators and constitutively active mutants of TFEB. First, we treated aNSCs with pharmacological activators of the lysosome, rapamycin and Torin-1. Both drugs significantly reduced cyclin D1 levels in aNSCs (new Fig. 5a, b). Second, we exogenously expressed two constitutively active mutants of TFEB (Settembre et al. EMBO J. 2012) (new Fig. 5 c-d). These mutants localized in the nucleus to greater extent than wild-type TFEB (new Fig. 5c). Because continuous expression of these mutants under the control of EF promoter in a construct delivered by a lentiviral vector dramatically decreased the number of TFEB-GFP–expressing cells in culture after several passages (not shown), we generated doxycycline (dox)-inducible lentiviral vectors. Exogenous TFEB mutants were expressed at the same levels as endogenous or wild-type TFEB in the presence of Dox on 1 day, but their levels gradually decreased on 2 and 3 days (new Fig. 5d). This result suggests that the number of cells expressing mutant TFEB-GFPs relative to that of non-infected cells decreased in culture, similar to the observation using the EF promoter as mentioned above. Consistent with this observation, the proportion of Ki-67–positive cells decreased to a greater extent in NSCs expressing TFEB mutants than in NSCs expressing wild-type TFEB (new Fig. 5e). These results indicate that TFEB activation (i.e., lysosomal activation) decreases proliferation in aNSCs. In the future, we plan to modulate the quiescence of NSCs using these mutants.

4. Some of the western blot experiments seem to lack quantification and statistical analyses.

According to this comment, we displayed quantification and statistical analyses in figures.

5. in Figure 3E and F, EGFR expression differences seem to be more pronounced in the immunostaining than the western blot.

This immunocytochemistry technique can only detect cytoplasmic EGFR internalized from the membrane surface because the membrane was permeabilized with detergent. This might be responsible for the differences between the immnostaining and western blot experiments.

6. When discussing the effect of DAPT, do the authors consider also Notch2? In contrast to Notch1, Notch2 may be involved in signaling quiescence.

We analyzed Notch2 expression in wild-type and TFEB-KO NSCs. However, Notch2 mRNA levels were lower than those of Notch1 in NSCs (about three times lower by qPCR, and another information in new Supplemental Table 2). In western blotting, Notch2 protein levels were fluctuated in NSCs *in vitro*, making it difficult to evaluate the involvement of Notch2 in our experiments.

7. Some of the cell biological experiments, e.g. those describing EGFR and P-EGFR turn over are difficult to follow to the authors' conclusions (page 7, lines 192-200). Eventually, when complex scenarios are being suggested, outcomes might be best modelled mathematically.

In response to this comment, we modeled the EGFR and P-EGFR turnover by mathematical simulation, as described in new Supplemental text. The model was helpful for assuming our proposed scenario.

8. Some of the immunostainings are not very impressive, including those of TFEB. What are the controls for the specificity of the antibodies used?

We do not have a better antibody than the one we used to detect TFEB. However, this antibody yielded clear bands in western blots (new Fig. 6a) and clear signals in immunocytochemistry (new Fig. 6b), both of which were diminished in TFEB-KO cells (new Fig. 6a, b). To confirm the specificity of this antibody in IHC, we used mice that were fasted for 1 day, because TFEB is highly activated in the fasting liver (Settembre et al, Nat Cell Biol. 2013). TFEB IHC yielded stronger signals in fasting liver than control liver (Figure 1 for reviewers), as previously reported. These results demonstrate the specificity of our anti-TFEB antibody.

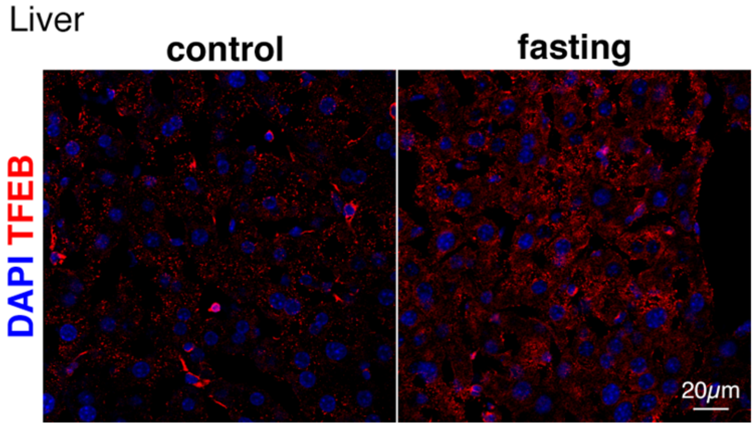

Figure 1 for reviewers. IHC of TFEB in liver from non-fasted and fasted mice. TFEB (red) was immunostained with DAPI (blue).

9. What is the contribution of an acute injury response to the slice culture experiments

We analyzed injury response genes in brain slices before and after culturing the slices in the presence or absence of BafA. The results are shown in new Supplemental Fig. 4f. These genes were highly upregulated in slices after culturing compared to slices without culturing, but did not exhibit differences between DMSO (control) and BafA-treated slices.

10. The in vitro experiments using the TFEB flox/flox cells are not well described? How is Cre introduced and transiently activated?

We described the source of Cre in our manuscript. Cre was transiently expressed under the control of the CAG promoter and introduced into cells by lipofection.

11. The in vivo effects of TFEB ko are not studied in sufficient detail. (A control wildtype image should be provided alongside Fig 6A). The fact that the reasons for the lack of increased neurogenesis following higher levels of aNSC is not pursued is unsatisfactory. It would be important to include more time points to reveal the dynamics of NSC activation, such as 7days, 14 days, 1 month, 2 months et al.

The control wild-type image was provided alongside with a TFEB KO image in new Supplemental Fig. 6 a, d. To address this comment, we analyzed aNSC dynamics by monitoring BrdU incorporation at two time points: day 6 (just 1 day after the last tamoxifen injection) and 4 weeks. The results are shown in new Fig. 7 a-d. TFEB KO mice (HOMO) increased aNSCs at both times relative to wild type (WT). Unexpectedly, TFEB heterozygote mice (HET) increased the abundance of aNSCs at 4 weeks to the same extent as HOMO mice, suggesting that the reduced level of TFEB in the heterozygote might gradually affect NSC activation, or that some compensation might reduce the number of aNSC in HOMO mice. However, at 12 weeks, the abundance of aNSCs was higher in HOMO mice than in HET mice (new Fig7e-j). These results suggest that the increase in the abundance of aNSCs following TFEB depletion might progress slowly, along with some unknown compensation machinery *in vivo*.

12.From the knock out experiments, it does not become clear how TFEB changes NSC activity. The following experiments should be included (1) Use BrdU to quantify cell cycle reentry. (2) Long term BrdU retention to label quiescent NSCs, then knock out TFEB in these labeled quiescent NSCs. (3) BrdU label active stem cells, then knock out TFEB in these labeled active NSCs.

We addressed experiment (1) above in our response to comment (11). We performed experiment (2) (long-term BrdU incorporation before knock out to label qNSCs) and experiment (3) (simultaneous injection of BrdU and tamoxifen to label aNSCs). Then, we analyzed mice on 10 days after tamoxifen administration, but the BrdU-labeled cells were very rare, and we observed no significant difference between KO (HOMO) and controls (HET and wild type) (Figure 2 for reviewers). Additional observation at later times (e.g. 1 month after tamoxifen administration) might be required to detect the differences.

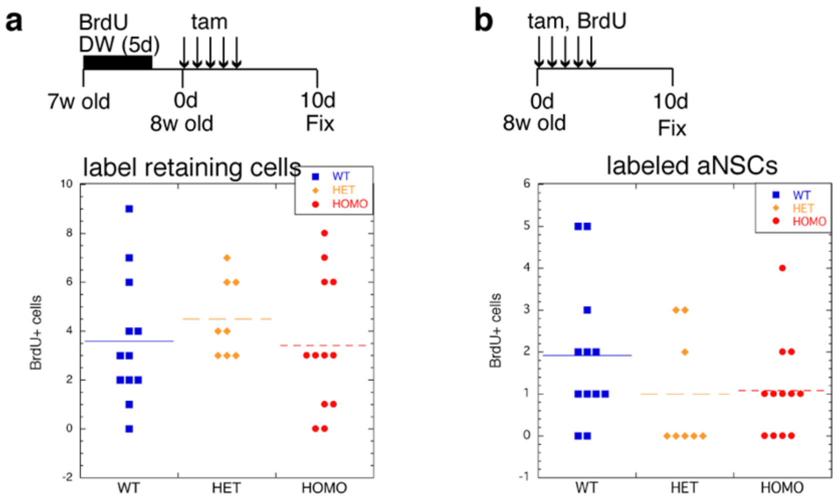

Figure 2 for Reviewers. BrdU-labeling experiments to label qNSC (a) and aNSC (b) in wild type (blue), heterozygote (yellow) and homozygote of TFEB cKO (red). Four slices per mouse were analyzed (n=3 (WT), n=2(HET), n=3(HOMO)).

Minor points:

The manuscript is somewhat disorganized. There is multiple switching back and forth with regard to figure numbering. Also, the experimental models used are multiple and sometimes without a clear statement for why a specific model is used rather than another. Some figures may be rather supplemental information, such as Figure 2 upper two panel rows.

We appreciate this comment. In the revised manuscript, we improved the figure numbering and explained it more clearly. The upper two rows from the original Figure 2 have been moved to new Supplemental Fig. 1a, b.

Reviewers' comments:

Reviewer #1 (Remarks to the Author):

My concerns have been addressed and I support publication of the manuscript in Nature Communications.

Reviewer #2 (Remarks to the Author):

The authors have improved the manuscript, however the overall evidence for lysosomal activity regulating return to quiescence remains uncompelling. See specific points below.

Comment #1. The authors attempted to address regulation of Notch1 (EGFR was not addressed) via immunoreactivity in wild type and cko Tfeb mice. The results are not entirely convincing. The image quality even at P0 is only limited. Would it be possible to do FACS-based analysis of Notch and EGFR expression levels?
If injection itself causes such damage that the effect of BafA could not be assessed in vivo in the SGZ, might this eventually work in the SVZ?
It is fully appreciated that the authors attempted the best to address my concerns. The question remains if the present data are compelling enough to follow the conclusions of the authors.

Comment #2. nice result.
Comment #3. What is the evidence that the effect of Torin-1 or rapamycin on cyclin D1 is via lysosomal activation? Could one follow return to quiescence following Tfeb constitutively activate mutants' expression by live imaging? Do these mutants increase the number of label retaining cells in the SGZ in vivo?
The comment in the rebuttal: "in the future...using the mutants" is a bit surprising. This effort should be included here. It could be the most compelling way to demonstrate the role of lysosomal activity in inducing quiescence.

Comment #4. Ok
Comment #5. Please state this in the results.

Comment #6. OK

Comment #7. Great

Comment #8. This is useful information and should be mentioned in the subplemental materials and methods.

Comment #9. Ok
Comment #10. Ok
Comment #11. Ok
Comment #12. It is a bit diffcult to understand why the experiments didn't yield any difference given the conclusions by the authors. Could it be that the experiments argue against a role of TFEB in inducing quiescence?

We would like to thank the editor and the reviewers for their valuable comments. We answered their comments one by one as below.

To the Editor:

In this regard, we expect to see additional analysis/data to highlight the expression profiles of EGFR and Notch1 in wild type and cko Tfeb mice.

We sought to reveal the expression profiles of these proteins in mice of wild type and *Tfeb* cKO. To this end, we first performed FACS-based analysis to quantify P-EGFR/EGFR and NICD/Notch1 levels, according to comment #1 of Reviewer #2. Although we tried many experimental conditions for flow cytometory, we detected no clear signals from NSCs. Moreover, for FACS analysis of tissue samples, dissociation using proteases is a critical step for generating single-cell suspensions. We found that the proteases dramatically decreased protein levels of membrane receptors, as reported previously (*Stem Cells* (2007) 25, 1560–1570). In western blotting, the protein bands were diminished, depending on the type of proteases used, and were undetectable in some samples (see new Figure for reviewers). For all of these reasons, we decided to subject DG dissected from *Tfeb*-cKO and control mice for western blotting. The results revealed significant increases in P-EGFR and NICD in the DG of *Tfeb*-cKO mice (new Supplemental Fig. 8). This observation indicates that these receptors accumulated due to lower lysosomal activity in the NSC niche of *Tfeb*-cKO mice.

Furthermore, we the editors agree with the reviewer that further support is needed to validate that lysosomal activity regulates return to quiescence (see Reviewers comments to #3 and #12).

To address this point, we conducted two experiments: live imaging of aNSCs expressing a constitutively active mutant of TFEB-GFP (caTFEB-GFP) (new Supplemental Fig. 5) and lentivirus injection to introduce caTFEB-GFP into the SGZ (new Fig. 5f-h). All results support the hypothesis that lysosomal activation by caTFEB induces quiescence *in vitro* and *in vivo*.

We feel that we have addressed all concerns from reviewers with new experimental data.
Accordingly, we believe that the manuscript is now suitable for publication in *Nature Communications.*

To Reviewer #1:

My concerns have been addressed and I support publication of the manuscript in Nature Communications.

Thank you very much. We greatly appreciate your supportive and constructive comments.

To Reviewer #2:

The authors have improved the manuscript, however the overall evidence for lysosomal activity regulating return to quiescence remains uncompelling. See specific points below.

Comment #1. The authors attempted to address regulation of Notch1 (EGFR was not addressed) via immunoreactivity in wild type and cko Tfeb mice. The results are not entirely convincing. The image quality even at P0 is only limited. Would it be possible to do FACS-based analysis of Notch and EGFR expression levels?

According to this comment, we performed FACS-based analysis to quantify P-EGFR/EGFR and NICD/Notch1 levels. Although we tried many experimental conditions for fixation, permeabilization, and immunostaining for flow cytometery, we could not detect clear signals relative to the negative control (normal IgG). For FACS analysis from tissue, dissociation using proteases is a critical step for generating single-cell suspensions. However, we found that proteases dramatically decreased the amount of membrane receptors, as reported previously (*Stem Cells* (2007) 25, 1560–1570). In western blotting, protein bands from protease-treated cells were weaker depending on the types of proteases used, and were undetectable in some samples (new Figure for reviewers b, c), although some tissue samples yielded clear bands (new Figure for reviewers a).

For these reasons, we subjected DG dissected from mice to western blotting. Significant increases in the levels of P-EGFR and NICD were detected in the DG of *Tfeb*-cKO mice (new Supplemental Fig. 8). This result indicates that these membrane receptors accumulated due to lower lysosomal activity in the NSC niche of *Tfeb*-cKO mice.

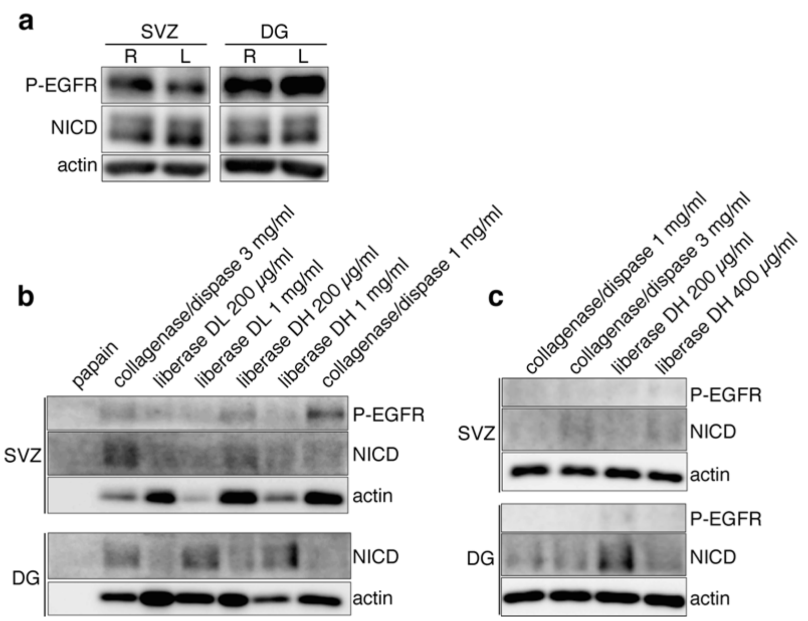

Figure for reviewers

a. Western blotting of P-EGFR, NICD, and actin in the SVZ and DG of wild-type mice. These NSC niches were dissected from the right (R) and left (L) hemispheres as described previously (Walker, T. L. (2014) *J. Vis. Exp.* 84, e51225, Hagihara H. (2009) *J. Vis. Exp.* 33, e1543), minced, and then lysed after homogenization in lysis buffer for western blotting. All bands were clearly detected.
b. c. Western blotting of protease-treated cells from the SVZ and DG of wild-type mice. Several types of proteases (papain, collagenase/dispase [Sigma], and liberases [Merck]), indicated at the top of the panels, were tested. Dissected niches (the SVZ and the DG) in a mouse were incubated with proteases for 10 min (for papain in **b**) or 30 min (for the other proteases: collagenase/dispase, liberase DL, and liberase DH) at 37□ and then triturated in 0.5 mg/ml DNase solution. Cells were purified by centrifugation with Percoll, treated with red blood cell lysis buffer (Sigma) (all protocols from *Cell Reports* (2019) 26, 394–406.e5), and analyzed by western blotting. The same number of live cells (1 or 2 × 10^4) was loaded in each well. Bands of P-EGFR and NICD were faint and not detectable in some samples.

If injection itself causes such damage that the effect of BafA could not be assessed in vivo in the SGZ, might this eventually work in the SVZ?

We performed BafA injection into the lateral ventricle using a glass capillary. However, we could not detect any changes or regions affected by the injection due to the diffusion in the ventricle. Continuous pump delivery of BafA would cause severe damage to the whole region surrounding the ventricle.

It is fully appreciated that the authors attempted the best to address my concerns. The question remains if the present data are compelling enough to follow the conclusions of the authors.

Thank you for your kind comments. We believe that our current revision addresses all of your concerns.

Comment #2. nice result.

Comment #3. What is the evidence that the effect of Torin-1 or rapamycin on cyclin D1 is via lysosomal activation? Could one follow return to quiescence following Tfeb constitutively activate mutants' expression by live imaging?
Do these mutants increase the number of label retaining cells in the SGZ in vivo?
The comment in the rebuttal: "in the future...using the mutants" is a bit surprising. This effort should be included here. It could be the most compelling way to demonstrate the role of lysosomal activity in inducing quiescence.

Thank you for your comments. We agree with your opinion about the effect of chemicals except regarding activation of TFEB and lysosomes; this is the reason why we analyzed constitutively active (ca) mutants of TFEB to induce quiescence in aNSCs (Fig. 5). To address this issue, we conducted two experiments to activate lysosomes *in vitro* and *in vivo*: live imaging of aNSCs expressing caTFEB-GFP (new Supplemental

Fig. 5) and lentivirus injection into the SGZ of adult mice to induce caTFEB-GFP expression in NSCs (new Fig. 5f-h).
For the first experiment, we monitored cell division of aNSCs by live imaging for 24 hours (new Supplemental Fig. 5 and new Supplemental movies). NSCs expressing wtTFEB-GFP divided, and the number of cells expressing GFP increased. By contrast, NSCs expressing caTFEB-GFP did not divide during this observation.
Second, we performed stereotactic injection of the lentiviral vector encoding TFEB-GFP driven by the *Hes5* or *Gfap* promoter into the DG (new Fig. 5f). The results of IHC (new Fig. 5g) revealed that caTFEB expression significantly decreased the number of Ki67-positive aNSCs in the DG (new Fig. 5h).
These results clearly demonstrate that lysosomal activity contributes to induction of quiescence.

Comment #4. Ok

Comment #5. Please state this in the results.

We described this as follows in the figure legend of Fig. 2e.

EGFR on the cell surface was not detected by immunostaining following membrane permeabilization. This might be responsible for the differences between the results of immunostainings and western blotting.

Comment #6. OK

Comment #7. Great

Comment #8. This is useful information and should be mentioned in the supplemental materials and methods.

We demonstrate the specificity of the TFEB antibody by showing IHC results in the new Supplemental Materials and Methods.

Comment #9. Ok

Comment #10. Ok

Comment #11. Ok

Comment #12. It is a bit difficult to understand why the experiments didn't yield any difference given the conclusions by the authors. Could it be that the experiments argue against a role of TFEB in inducing quiescence?

The time point used in these experiments might have been too early to detect the clear differences in BrdU-incorporating cells. It is plausible that lysosomal dysfunction in qNSCs due to TFEB depletion might gradually progress along with unknown cellular compensation machinery, resulting in no difference in the results of these experiments.

REVIEWERS' COMMENTS:

Reviewer #2 (Remarks to the Author):

All remaining questions have been addressed adequately by the authors.

Review signed
Benedikt Berninger
